# Efficient Approach for the Synthesis of Aryl Vinyl Ketones and Its Synthetic Application to Mimosifoliol with DFT and Autodocking Studies

**DOI:** 10.3390/molecules28176214

**Published:** 2023-08-24

**Authors:** Tummuri Sudheer Reddy, Karreddula Raja, Kishore Reddy Mandapati, Srinivasa Reddy Goli, Manubolu Surya Surendra Babu

**Affiliations:** 1 Department of Chemistry, GITAM University Hyderabad Campus, Hyderabad 502329, Telangana, India; tummurisudheer1981@gmail.com; 2 Department of Chemistry, Rajeev Gandhi Memorial College of Engineering and Technology (Autonomous), Nandyal 518501, Andhra Pradesh, India; rajachem@rgmcet.edu.in; 3 Synaptics Labs Private Limited, Kurmannapalem, Matrusri Nagar, Gajuwaka, Visakhapatnam 530026, Andhra Pradesh, India

**Keywords:** mimosifoliol, quinolines, aspulvinone dimethylallyltransferase, molecular docking

## Abstract

An efficient and elegant method was developed for the preparation of substituted phenyl vinyl ketones using low-cost and commercially available ethyl chloroformate and diisopropylethylamine as reagents. This methodology was also applied to the synthesis of natural products such as mimosifoliol and quinolines. Frontier molecular orbital (FMO) studies on mimosifoliol were carried out to understand its chemical reactivity. Electron localization function (ELF) and localized orbital locator (LOL) analysis gave information about localized and delocalized electrons. Reduced density gradient (RDG) analysis gave information on steric, van der Waals, and hydrogen-bonding interactions. Molecular electrostatic potential (MEP) and Fukui functions gave information about nucleophilic and electrophilic attack. Nonlinear optical (NLO) analysis represented the mimosifoliol good NLO material. Molecular docking showed that the mimosifoliol compound had effectively inhibited the aspulvinone dimethylallyltransferase enzyme.

## 1. Introduction

The construction of a conjugated double bond to a carbonyl group has attracted continuous attention from synthetic chemists [1,2,3], and it is extensively studied because the compounds containing C=C bonds are widely present in many naturally occurring compounds [4,5,6,7]. Vinyl ketones have been widely used in synthesis as excellent Michael acceptors, dienophiles, and monomers [8]. In synthetic chemistry, the connection of two different molecules with a linker is a fundamental design principle of functional organic molecules such as organic devices [9]. These vinyl ketone systems have a wide range of applications in the synthesis of complex molecules via Michael addition [9], Robinson annulation [10], aldol reactions, and olefin cross-metathesis. The wide range of applications attracted us to develop a new and efficient methodology to get desired aryl vinyl ketones from β-amino-carbonyl compounds, also known as Mannich bases [11]. 

Aryl vinyl ketones are very useful in many applications, such as degradation thermoplastics [12], Diels order reactions [13], preparation of chalcones [14], and Heck reactions [15]. In recent years, α-methylenation of aromatic ketone for carbon sources has been achieved by utilizing methanol in the presence of magnesium oxide and magnesium phosphate [16], paraformaldehyde in the presence of diisopropylammonium trifluroacetate and catalytic acid or base [17], trifluroacetate and DMF in the presence of copper catalyst [18], selectfluor in the presence of gold catalyst [19], dimethyl sulfoxide under metal-free conditions [20], and methanol in the presence of Cu@g-N4 catalyst [21]. 

While the previously used methods mainly incorporated the use of metal catalysts, in order to overcome this restriction, we adopted the use of 1,2-dichloroethane (DCE) and N,N-diisopropylethylamine (DIPEA), both of which are readily available and economically feasible. This decision was carefully made, taking flexibility, environmental concerns, and practicality into account. As a result, we created a strategy that presents an effective and sustainable path for the efficient creation of C=C bonds. It will be of great importance in organic synthesis, and this method avoids the use of the expensive methyl iodide from Mannich bases. We synthesized a couple of substituted aryl vinyl ketones with good yields from their corresponding Mannich salts, which were easily accessed from aryl methyl ketones by using paraformaldehyde and dimethylamine in isopropyl alcohol as a solvent under reflux conditions. It is clearly demonstrated that this method has the capability and tolerance of different substitutions and different aromatic systems. Moreover, we also achieved the corresponding quinolines from 2-fluorophenyl vinyl ketone with methyl- and benzylamines in DMF solvent with decent yields.

## 2. Results

**Scheme 1:**
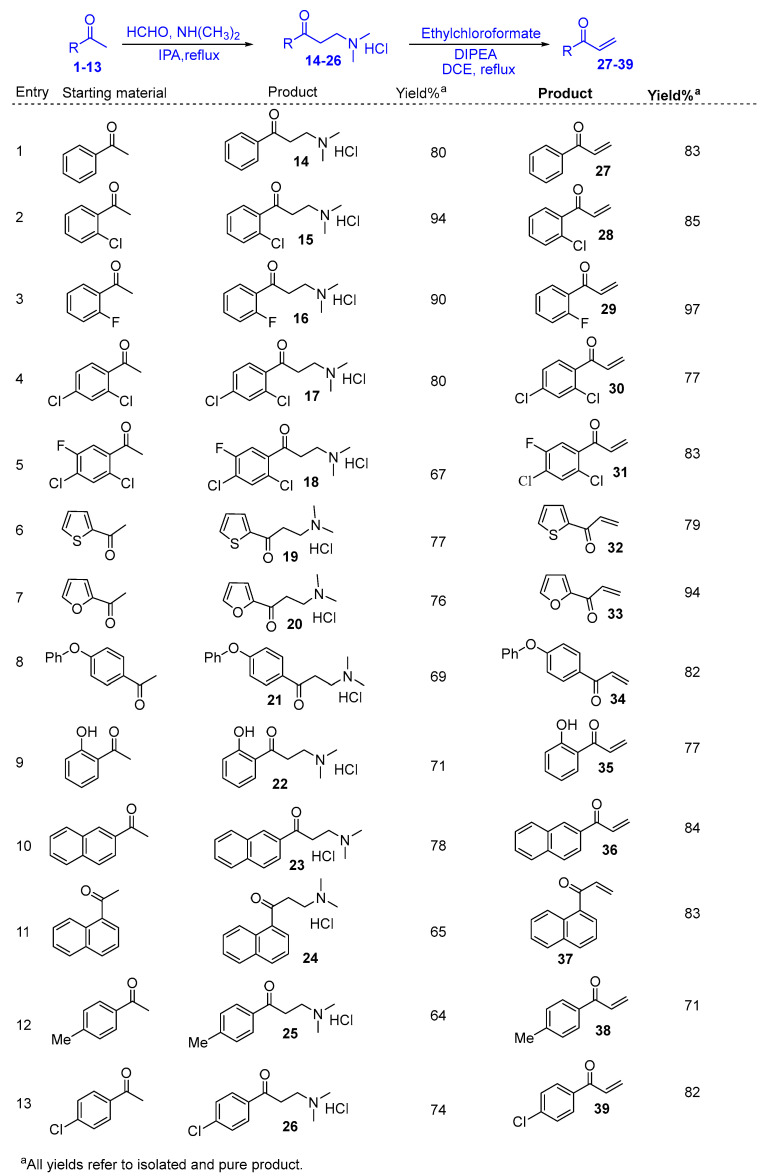
Synthesis of aryl vinyl ketones.

**Scheme 2:**
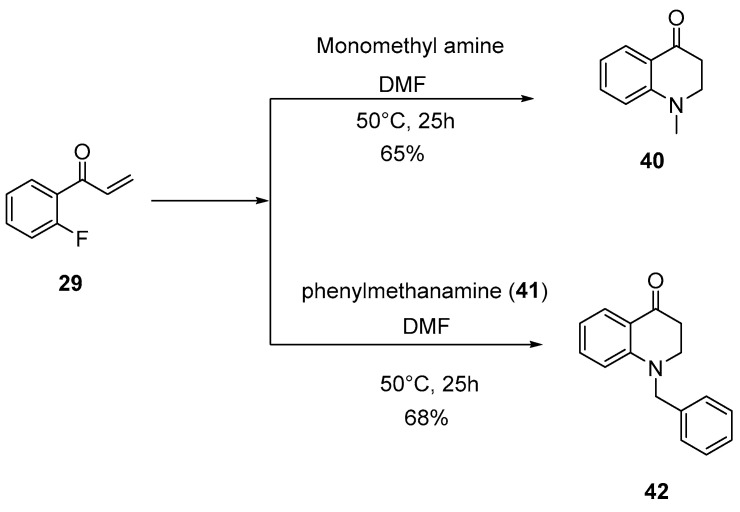
Synthesis of quinolines.

**Scheme 3:**
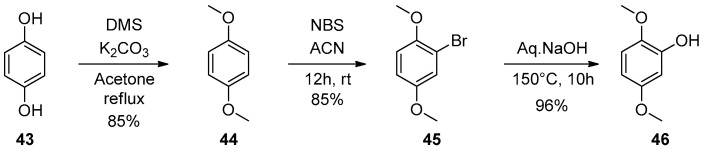
Synthesis of 2,5-dimethoxyphenol.

**Scheme 4:**
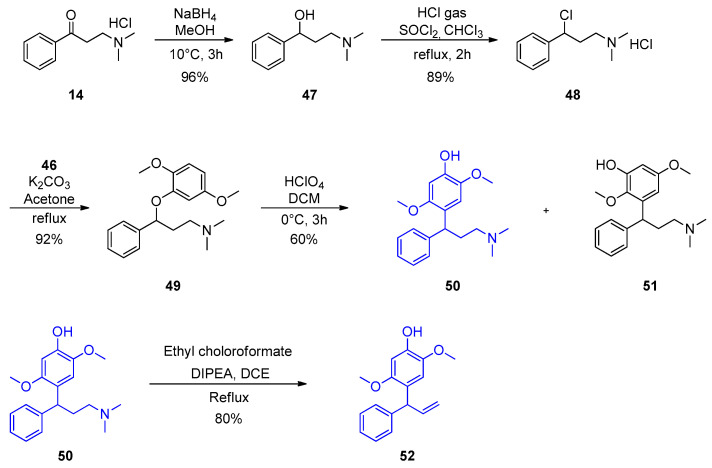
Synthesis of mimosifoliol.

Aryl methyl ketones (**1**–**13**) were reacted with dimethylamine hydrochloride and paraformaldehyde in the presence of a catalytic amount of concentrated hydrochloric acid in 2-propanol at reflux temperature to yield Mannich base hydrochlorides (**14**–**26**). It was further treated with ethyl chloroformate and diisopopylamine in 1,2-dichloroethane to get aryl vinyl ketones (**27**–**39**). Scheme 1 shows the synthesis in detail. Aryl vinyl ketones were reacted with aryl amine or alkyl amine in DMF to get quinolines (**40** and **42**) in good yields (Scheme 2). Intermediate (**46**) was prepared using hydroquinone (**43**), which was treated with dimethyl sulfate and potassium carbonate in acetone to get (**44**) in 85% yield. This 1,4-dimethoxy benzene (**44**) was treated with N-bromosuccinamide in acetone at room temperature to get (**45**) with 85% yield. This 2-bromo-1,4-dimethoxybenzene (**45**) compound was treated with aqueous sodium hydroxide to get 2,5-dimethoxyphenol (**46**) with 96% yield. The details are shown in Scheme 3. Mimosifoliol natural product is a neoflavonoid from the rootwood of *Aeschynomene mimosifolia.* We synthesized mimosifoliol starting from acetophenone, which was converted to 3-dimethylamino-1-phenylpropan-1-one hydrochloride (**14**) 80% yield. This dimethyl amine salt (**14**) was treated with sodium borohydride in methanol at 10 °C to get 3-dimethylamino-1-phenylpropan-1-ol (**47**) with 96% yield. This alcohol (**47**) was treated with hydrochloric acid and thionyl chloride in chloroform at reflux temperature for 2 h to get (3-chloro-3-phenylpropyl dimethyl amine (**48**) with 89% yield. We carried out a condensation reaction with (**46**) and (**48**) in acetone and potassium carbonate to get ether (**49**) with 92% yield. The subsequent rearrangement reaction with perchloric acid in dichloromethane at 0 °C to get regio alcohols (**50** and **51**) with 40% and 20% yields. 4-Hydroxy alcohol (**50**) was treated with ethyl chloroformate and DIPEA in ethylene chloride to get mimosifoliol (**52**) in 80% yield. The details are shown in Scheme 4. The analytical data attached in supporting information.

*Proposed reaction mechanism*: Step 1

The ammonium chloride salt is formed from the treatment of the amine with a beta hydrogen or quaternary ammonium with ethyl chloroformate, as shown below.


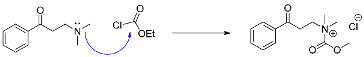


Step 2: 

The substitution of the chloride ion reaction with a base is heated to facilitate an elimination reaction and form the required product.


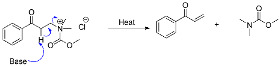


### 2.1. Spectroscopic Studies

Using KBr pellets, infrared spectral data of the title compound were recorded in the range of 4000–400 cm^−1^. Stretching vibrations of C–H bonds were assigned to aromatic, vinyl, or methyl C–H groups in the experimental spectrum and were shown to occur between the wavelengths of 3341 and 2895 cm^−1^ [22]. The stretching vibrations related to C=C bonds were found to vary between 1615 and 1300 cm^−1^ related to benzene rings, five-membered heterocyclic rings, naphthalene rings, or vinyl groups [22]. The links between carbonyl carbon and benzene rings, as well as between carbonyl carbon and vinyl group carbons, are shown by the vibration mode corresponding to C–C stretching. These vibrations were noted between 1243 and 1056 cm^−1^. The O–H stretching vibration mode, characterized by a large peak, was seen in the spectra of compound 35 and mimosifoliol at 3464 cm^−1^ and 3342 cm^−1^, respectively.

The title compound’s ^1^H- and ^13^C-NMR spectra were obtained (internal standard, TMS; solvent, DMSO-d6 or CDCl_3_). Identified in the ^1^H-NMR spectrum were distinct proton signals from various aromatic rings: the benzene ring protons resonated between 6.43 and 7.95 ppm, the five-membered ring (furan or thiophene) protons appeared between 6.26 and 7.78 ppm, and the naphthalene ring protons displayed signals between 7.26 and 8.36 ppm. At 2.39 ppm in compound **38**, the methyl (CH_3_) group signals were detected as a singlet [23]. At 12.52 ppm, compound **35** exhibited the singlet signal associated with the hydroxyl (OH) group, which was attributed to intramolecular hydrogen bonding between the O–H group and nearby O atom in the carbonyl (C=O) moiety [23]. 

Carbonyl carbon peaks, which correspond to phenyl vinyl ketones and naphthalene vinyl ketones, were found in the ^13^C-NMR spectra in the signal range of 189.34–195.91 ppm. Notably, these carbonyl carbon peaks were found at 182.31 and 177.43 ppm for compounds **32** and **33**, respectively. Carbon atoms connected to benzene or naphthalene rings showed peaks between 116.56 and 163.59 ppm, whereas carbon atoms connected to vinyl groups were seen between 128.86 and 136.68 ppm.

### 2.2. Geometry Optimization

**Figure 1:**
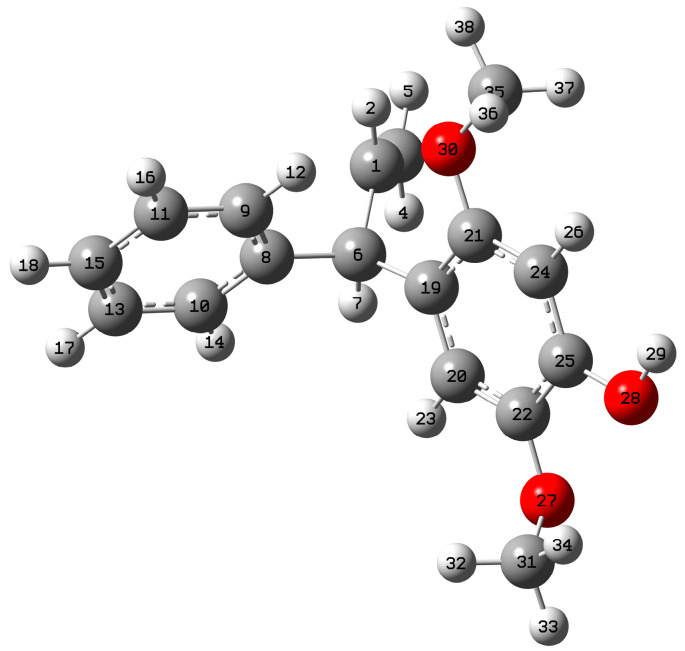
Optimized structure of mimosifoliol.

The mimosifoliol geometry was optimized using the Gaussian 09 program with the B3LYP/6-31++G (d, p) basis set, as shown in Figure 1. There was no imaginary frequency observed during vibrational analysis, representing a completely optimized structure.

### 2.3. Frontier Molecular Orbital (FMO) Studies 

**Figure 2:**
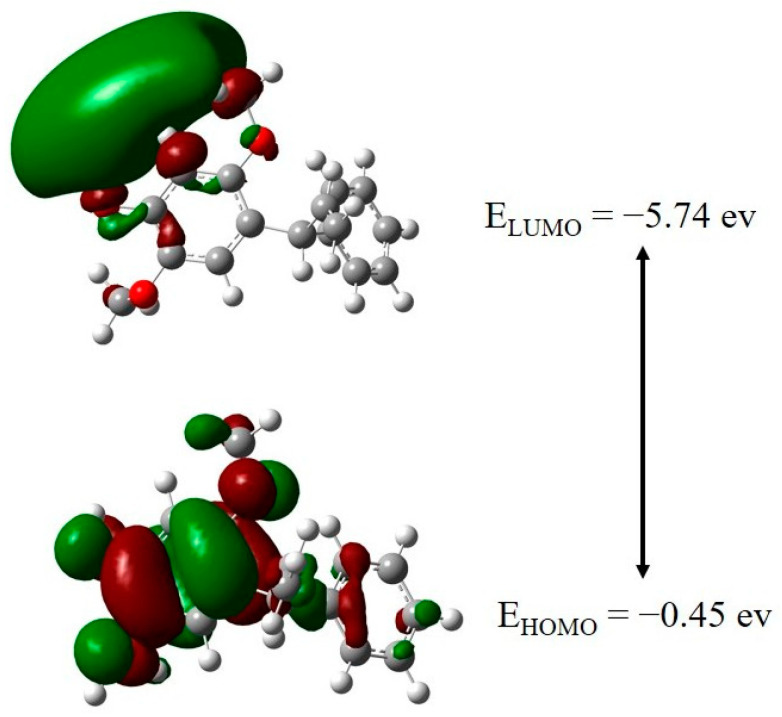
HOMO and LUMO FMO of mimosifoliol compound.

HOMO–LUMO analysis is very useful for analyzing the chemical reactivity and conductivity of a molecule [24,25]. The HOMO orbital represents electron-donating capability, while the LUMO orbital represents electron-acceptor capability. For the mimosifoliol compound, HOMO, LUMO, and band gaps were −5.74 eV, −0.45 eV, and 5.29 eV respectively. The band gap value of a molecule represents its stable and biologically active compound. The FMO of HOMO and LUMO for the mimosifoliol is represented in Figure 2. Various global parameters were calculated: chemical potential (μ) = −3.1857 eV, global hardness = η = 5.1132 eV, electrophilicity = ω = 0.9924 eV, electronegativity χ = −μ = 3.1857 eV, and softness S = 1/2 η = 0.0978 eV.

### 2.4. Molecular Electrostatic Potential (MEP) Analysis

**Figure 3:**
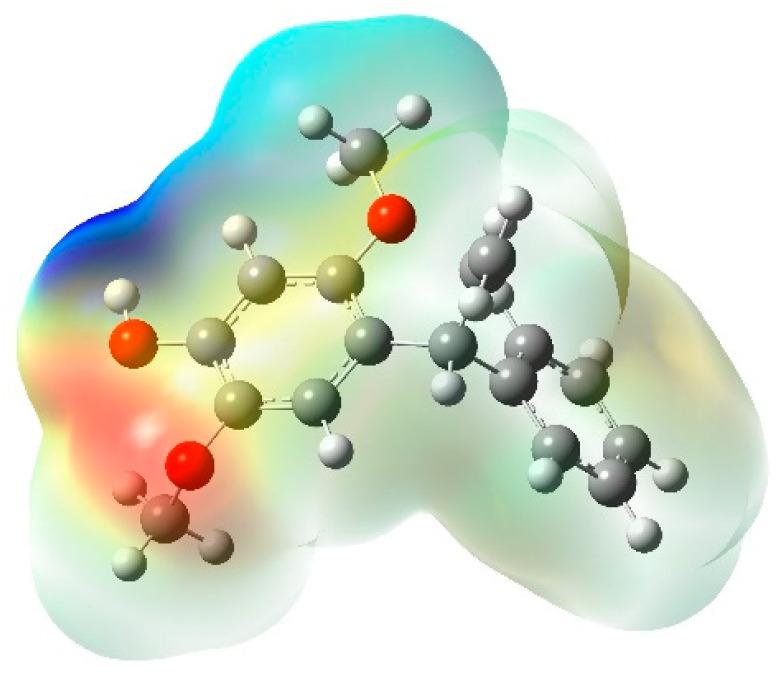
MEP map of mimosifoliol.

MEP analysis is very useful to learn about electrophilic and nucleophilic interactions in chemical reactions [26]. Figure 3 depicts the MEP map of the mimosifoliol compound. The MEP map is represented with different colors. Red, green, and blue colors represent −ve, 0, and +ve electrostatic potential values, respectively. The MEP map is plotted using color ranges from −4.825 × 10^−2^ to 4.825 × 10^−2^. In mimosifoliol compound, the red color at the O atom represents electrophilic attack and the blue color at the hydrogen of OH represents nucleophilic attack.

### 2.5. NBO Analysis

The stabilization energy is calculated as follows:

$$E(2)=∆E_{i,J}=\mathrm{qi}\frac{{\left(F_{i,j}\right)}^{2}}{E_{j}-E_{i}}$$

Stabilization energies were obtained using the second-order perturbation method for mimosifoliol and are presented in Table 1. More stabilization energy represents strong interaction between the donor and acceptor. For mimosifoliol, important interactions are π(C8–C10) to π*(C9–C11/C13–C15) (19.31/21.23 kcal/mol), π(C9–C11) to π*(C8–C10/C13–C15) (20.6/19.94 kcal/mol), π(C13–C15) to π*(C8–C10/C9–C11) (19.09/20.09 kcal/mol), π(C19–C20) to π*(C21–C24/C22–C25) (23.04/18.09 kcal/mol), π(C21–C24) to π*(C19–C20/C22–C25) (14.67/21.9 kcal/mol), and π(C22–C25) to π*(C19–C20/C21–C24) (22.56/17.01 kcal/mol). These transitions occur within the benzene and phenol rings of mimosifoliol. Another important transition is lone pair to π* such as LP(2) O28 to π*(C22–C25) and LP(2) O30 to π*(C21–C24), with stabilization energy of 25.77 and 28.58, respectively. Selected NBO calculations for mimosifoliol are mentioned in Table 1.

### 2.6. Electron Localization Function (ELF) Analysis

**Figure 4:**
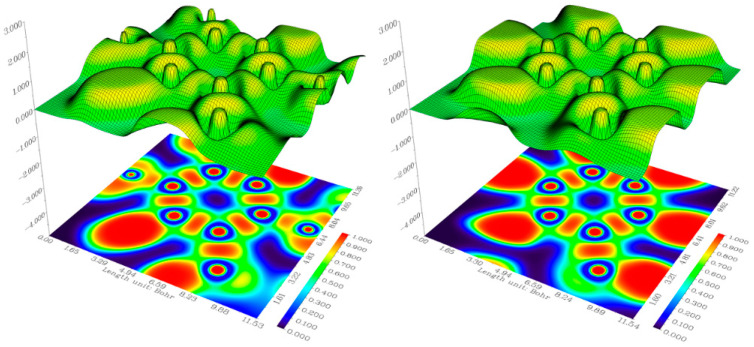
ELF for mimosifoliol.

ELF analysis is very useful for analyzing quantitative aromaticity [27,28]. The Multiwfn 3.8 program was used for ELF analyses via relief map for mimosifoliol compound, as shown in Figure 4. ELF analysis depends on Pauli repulsion. If Pauli repulsion is maximal, it gives a high ELF value, which is represented with a red color, whereas, if Pauli repulsion is low or near zero, it gives a low ELF value, which is represented with a blue color. The red color represents localized electrons, whereas the blue represents delocalized. Localized orbitals correspond to lone pair electrons, chemical bonds, and atomic shells. In mimosifoliol compound, red color is observed around hydrogen due to a single electron with high Pauli repulsion, while blue color is observed around C (benzene and phenol rings) and O due to close-by same-sign electrons [29]. The red color indicates the formation of a bond between two atoms (C–C and C–O).

### 2.7. Localized Orbital Locator (LOL) Analysis

**Figure 5:**
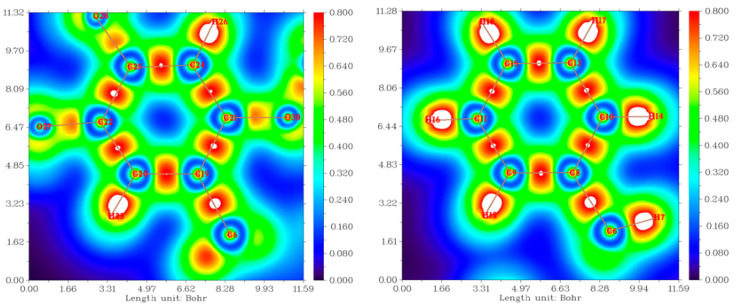
LOL of mimosifoliol.

The Multiwfn 3.8 program was used for LOL analyses via a colored filled map for the mimosifoliol compound, as shown in Figure 5. LOL studies are very useful for knowing about localized, delocalized orbitals, and chemical bonding in molecules [30,31]. In the LOL map, the area around the hydrogen white color spot represents more electron density than the color scale limits. Red or orange color represents **covalent** bonds formed between C–C and C–O. The blue circle around C (benzene and phenol ring) and O atoms represents the electron depletion area between the valance and inner shells.

### 2.8. Reduced Density Gradient (RDG) Analysis

RDG analysis is a very useful tool to explain intra- and inter-molecular interactions. RDG analysis for mimosifoliol was carried out by the Multiwfn 3.8 program. RDG analysis was obtained for electron density and its gradient.

$$\mathrm{RDG}(r)=\frac{1}{{2\left(3\pi^{2}\right)}^{1/3}}\frac{\left|\nabla \rho \left(r\right)\right|}{{\rho (r)}^{4/3}}$$

**Figure 6:**
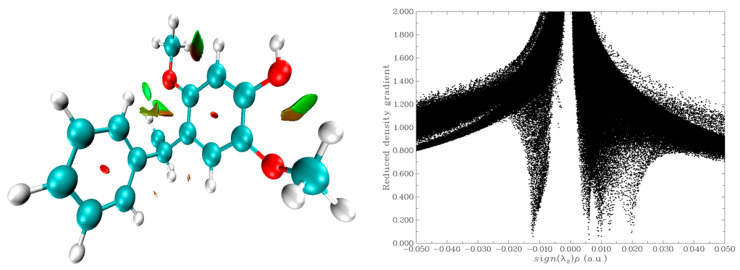
RDG iso surface and scatter graph of mimosifoliol.

The (λ2) *p* values explain the type of interactions, where (λ2) *p* > 0 denotes repulsion, (λ2) *p* < 0 denotes hydrogen bonds, and (λ2) *p* ≈ 0 denotes Vdw interactions. The 3D RDG isosurface density map was generated by the VMD program. The 2D scatter graph and 3D RDG of mimosifoliol are shown in Figure 6. In the RDG graph, red represents the steric interactions of ring molecules, green represents weak van der Waals interactions, and blue represents strong hydrogen-bonding interactions.

### 2.9. Fukui Functions

Fukui indices were obtained from FMO rather than NPA [32]. Fukui function analysis is very useful to determine which atoms loss or gain electrons in a molecule [33]. At the r-th atomic site, the condensed or atomic function is calculated as follows:

$$f^{+}(r)\;=\;\rho_{N+1}(r)\;-\;\rho_{N}(r)$$

$$f^{-}(r)=\rho_{N}(r)\;-\;\rho_{N-1}(r)$$

$$f^{0}(r)\;=\;\frac{1}{2}\;[f^{+}\left(r\right)+f^{-}(r)]$$

$$∆f=f^{+}\left(r\right)-f^{-}(r)$$

∆*f*(*r*) gives useful information to distinguish between nucleophilic and electrophilic sites in a specific region. Positive values (∆*f* > 0) prefer nucleophilic attacks, whereas negative values (∆*f* > 0) prefer electrophilic attacks. The atom-condensed Fukui function for mimosifoliol is presented in Table 2. The analysis dual descriptor of mimosifoliol compound favored nucleophilic attack at 29H > 26H > 36H > 24C > 37C > 20C > 38H > 34H and electrophilic attack at 25C > 35C > 22C > 21C > 19C > 8C > 28O > 37O > 27O.

### 2.10. NLO Analysis

NLO analysis was carried out at the B3LYP/6-31++G (d, p) level of theory for mimosifoliol. Mimosifoliol showed a dipole moment, polarizability, and first-order hyperpolarizability calculated as follows:

$$\mu ={{(\mu }_{x}^{2}+\mu_{y}^{2}+\mu_{z}^{2})}^{1/2}$$

$$\alpha_{total}=\frac{\alpha_{xx}+\alpha_{yy}+\alpha_{zz}}{3}$$

$$\beta_{total}={(\beta }_{x}^{2}+\beta_{y}^{2}+\beta_{z}^{2}){}^{1/2}$$

The dipole moment of mimosifoliol was 1.39 D, and its polarizability (*α*) was 32.04 × 10^−24^ esu. Urea was considered the reference for the NLO analysis for comparison. The hyperpolarizability (*β*) of mimosifoliol was 3.4 × 10^−30^ esu, which is 10 times greater than that of urea (0.372 × 10^−30^) [34]. These values confirm that the mimosifoliol showed nonlinear optical properties.

### 2.11. Drug Likeness and ADMET Analysis

The Swiss ADMET online tools [35] were used to calculate drug-likeness properties for mimosifoliol, as mentioned in Table 3. Mimosifoliol obeyed the Lipinski rule of five, as well as the Veber, Ghose, Egan, and Muegge rules. Pharmaco-kinetics were calculated using the pre-ADMET tool for mimosifoliol, as mentioned in Table 4. Mimosifoliol showed excellent human intestinal absorption (>70), medium Caco-2 permeability (10–100 nm/s), good skin permeability, moderate absorption of the central nervous system, and excellent binding with plasma protein. The bioactivity of mimosifoliol was calculated using the Molinspiration online tool and is mentioned in Table 5. A bioactivity score > 0 represents a more biologically active molecule, a score between −0.5 and 0 represents a moderately active molecule, and a score < 0.5 represents an inactive drug. On the basis of its bioactivity score, mimosifoliol is biologically active and interacts with GPCR ligands, kinase inhibitors, nuclear receptors, and other enzymes.

### 2.12. Docking Analysis

**Figure 7:**
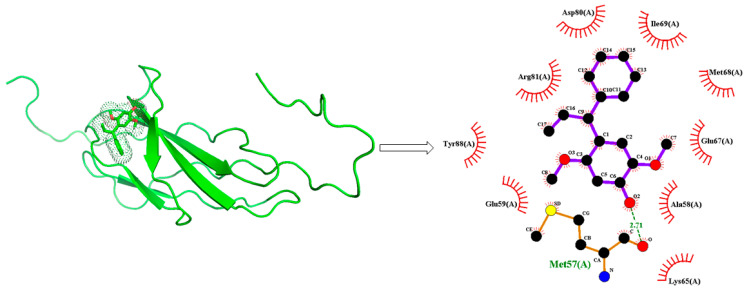
Docking results of mimosifoliol with 2CRM (green Color dotted line-Hydrogen bond).

In the drug discovery process, molecular docking is a very effective technique to analyze target (protein) and drug interaction [36]. Molecular docking (rigid docking) was carried out for the mimosifoliol compound utilizing Autodock 4.2. [37] According to the pass online program [38], mimosifoliol compound showed aspulvinone dimethylallyltransferase inhibition along with association and dissociation values of 0.893 and 0.009, respectively. The Forth FNIII domain of the human (PDB ID: 2CRM) target receptor was obtained from RCSB PDB (https://www.rcsb.org, accessed on 5 July 2022). The unwanted chain, hetero atom, and water molecules were removed, and polar hydrogens and Kollman charges were added for preparation of the protein in Autodock4. The best docking binding energy between ligand and receptor was −5.62 kcal/mol. The receptor–ligand interaction showed one hydrogen bond (Met57) with a bond length of 2.71 Å. The best docking image for the ligand–receptor interaction is shown in Figure 7. These studies represent that mimosifoliol compound effectively inhibits the aspulvinone dimethylallyltransferase enzyme.

## 3. Materials and Methods

All the chemicals employed in the present investigation were purchased from commercial sources and used without any further purification. Analytical thin-layer chromatography was carried out using E-Merck 60F254 aluminum-backed plates of silica gel (0.2 mm) purchased from Chemtech International, Gujarat, India. Developed plates were visualized using UV light or potassium permanganate solution. Column chromatography was performed on silica gel (100–200 mesh). IR spectra were recorded in the range 4000–400 cm^−1^ with a total of 256 scans on a Perkin Elmer 100 FT-IR spectrometer with a DTGS detector using a KBr pellet. ^1^H- and ^13^C-NMR spectra were obtained in CDCl3 or DMSO on a Bruker AV 400 MHz spectrometer. Chemical shifts (δ) were reported in parts per million (ppm) utilizing TMS as an internal reference and coupling constants (J) in hertz (Hz). Splitting patterns of the NMR signals were described as br—broad, s—singlet, d—doublet, t—triplet, q—quartet, and m—multiplet. Mass spectra (MS) were recorded on an API 2000 LCMS/MS AB Sciex spectrometer.

### 3.1. 3-Dimethylamino-1-phenylpropan-1-one hydrochloride (***14***)

To a stirred solution of acetophenone (**1**) (10 g, 83.23 mmol) in 2-propanol (30 mL) was added N,N-dimethylamine hydrochloride (8.55 g, 104.86 mmol), paraformaldehyde (3.75 g,124.8 mmol), and 50% aqueous hydrochloride (5 mL) at room temperature, and the reaction mixture was stirred at 85 °C for 25 h and monitored by TLC. The reaction mixture was cooled to room temperature and then to 5–10 °C for 1 h; the solid was filtered and then washed with chilled 2-propanol. The solid was then dried at 60 °C for 5 h to give compound **14 [39]** as a white solid (14 g, 80%), mp 116.2–118.8 °C: ^1^H-NMR (400 MHz, DMSO-*d_6_*) δ 11.13 (br s, 1H), 8.04 (d, *J* = 7.6 Hz, 2H), 7.71 (t, *J* = 7.2 Hz, 1H), 7.59 (t, *J* = 7.6 Hz, 2H), 3.70 (t, *J* = 7.4 Hz, 2H)3.44 (t, *J* = 8.6 Hz, 2H), 2.81 (s, 6H); ^13^C-NMR (100 MHz, DMSO-*d_6_*) *δ* 197.20, 136.39, 134.17, 129.27, 128.48, 52.13, 42.55, 33.64; FT-IR (KBr) ν_max_3433.92, 2643.09, 1679.74, 960.32, 754.30 cm^−1^; MS: *m*/*z* 178.1 (M + H).

Experimental data compound (**15**). (See Supplementary Materials).

### 3.2. 3-Dimethylamino-1-(2-fluorophenyl)propan-1-one hydrochloride (***16***)

Compound **16** as a white solid (90%), mp 177.4–1179.2 °C: ^1^H-NMR (400 MHz, DMSO-*d_6_*) δ 11.15 (s, 1H), 7.92 (t, *J* = 7.2 Hz, 1H), 7.74 (q, *J* = 6.5 Hz, 1H), 7.42–7.36 (m, 2H), 3.61 (t, *J* = 6.4 Hz, 2H), 3.42 (t, *J* = 7.6 Hz, 2H), 2.78 (s, 6H); ^13^C-NMR (100 MHz, DMSO-*d_6_*) *δ* 195.00, 194.97, 162.85, 160.31, 136.11, 136.02, 130.81, 130.80, 125.34, 125.31, 125.09, 124.97, 117.54, 117.34, 51.86, 42.64, 37.62; FT-IR (KBr) ν_max_3128.26, 1693.24, 1391.51, 770.21 cm^−1^; MS: *m*/*z* 196.1 (M + H).

Experimental data compound (**17**–**26**). (See Supplementary Materials).

### 3.3. 1-Phenyl-propenone (***27***)

To a stirred solution of 3-dimethylamino-1-phenylpropan-1-one hydrochloride (**14**) (8 g, 37.48 mmol) in ethylene dichloride (60 mL) was added diisopropylethylamine (7.27 g, 56.22 mmol), followed by ethyl chloroformate (6.1 g, 56.22 mmol), and the resulting mixture was refluxed for 5 h under nitrogen atmosphere. After cooling to room temperature, the solvent was evaporated. Purification of the residue by column chromatography (silica gel, only hexane) provided **27 [25]** as a colorless syrup (4.08 g, 83%): ^1^H-NMR (400 MHz, CDCl_3_) *δ* 7.95 (d, *J* = 7.6 Hz, 2H), 7.59 (t, *J* = 7.4 Hz, 1H), 7.49 (t, *J* = 7.6 Hz, 2H), 7.19 (dd, *J* = 17.2, 10.8Hz, 1H), 6.46 (dd, *J* = 16.8, 0.8 Hz, 1H), 5.94 (d, *J* = 10.8, 1.2 Hz,1H); ^13^C-NMR (100 MHz, CDCl_3_) *δ* 190.87, 137.23, 132.98, 132.34, 130.06, 128.65, 128.61; FT-IR (Neat) ν_max_1677.64, 1588.27, 1257.55, 779.20 cm^−1^; MS: *m*/*z* 132.9 (M + H).

### 3.4. 1-(2-Chloro-phenyl)propenone (***28***)

Compound **28 [18]** as a colorless syrup (85%): ^1^H-NMR (400 MHz, CDCl_3_) *δ* 7.42–7.35 (m, 3H), 7.34–7.28 (m, 1H), 6.79 (dd, *J* = 17.6, 10.4 Hz, 1H), 6.15 (d, *J* = 17.6 Hz, 1H), 6.03 (d, *J* = 10.4 Hz, 1H); ^13^C-NMR (100 MHz, CDCl_3_) *δ* 194.24, 138.15, 136.16, 132.01, 131.53, 131.30, 130.27, 129.30, 126.73; FT-IR (Neat) ν_max_3419.95, 1649.13, 1268.98, 969.58, 752.32 cm^−1^; MS: *m*/*z* 167.0 (M + H).

Experimental data of compound (**29**–**31**). (See Supplementary Materials).

### 3.5. 1-Thiophen-2-yl-propenone (***32***)

Compound **32 [21]** as a colorless syrup (79%): ^1^H-NMR (400 MHz, CDCl_3_) *δ* 7.78 (d, *J* = 3.6 Hz, 1H), 7.68 (d, *J* = 4.8 Hz, 1H), 7.17 (t, *J* = 4.2 Hz, 1H), 7.11 (dd, *J* = 17.2, 10.8 Hz, 1H), 6.52 (d, *J* = 16.8 Hz, 1H), 5.89 (d, *J* = 10.0 Hz, 1H); ^13^C-NMR (100 MHz, CDCl_3_) *δ* 182.31, 144.53, 134.41, 132.54, 131.80, 129.32, 128.34; FT-IR (Neat) ν_max_1710.52, 1008.85, 780.54 cm^−1^; MS: *m*/*z* 138.9 (M + H).

Experimental data of compound (**33**–**35**). (See Supplementary Materials).

### 3.6. 1-Naphthalen-2-yl-propenone (***36***)

Compound **36 [19]** as a colorless syrup (84%): ^1^H-NMR (400 MHz, CDCl_3_) *δ* 8.36 (s, 1H), 7.98 (d, *J* = 8.8 Hz, 1H), 7.86–7.75 (m, 3H), 7.52–7.43 (m, 2H), 7.27 (dd, *J* = 17.2, 10.8 Hz, 1H), 6.48 (dd, *J* = 17.2, 1.2 Hz, 1H), 5.89 (dd, *J* = 10.4, 0.8 Hz, 1H); ^13^C-NMR (100 MHz, CDCl_3_) *δ* 190.61, 135.54, 134.58, 132.48, 132.31, 130.41, 129.97, 129.56, 128.60, 128.53, 127.81, 126.82, 124.40; FT-IR (Neat) ν_max_ 2971.27, 1667.97, 1402.01, 792.34 cm^−1^; MS: *m*/*z* 183.1 (M + H).

Experimental data of compound (**37**–**39**). (See Supplementary Materials).

### 3.7. Preparation of Quinolins Using Phenyl Vinyl Ketone

#### 3.7.1. 1-Methyl-2,3-dihydro-1H-quinolin-4-one (**40**)

A mixture of 2′-fluorophenyl vinyl ketone (0.5 g, 3.35 mmol) and methyl amine (0.2 g, 6.71 mmol) in DMF (10 mL) was stirred at 50 °C for 9 h and monitored by TLC. The reaction mixture was diluted with ethyl acetate (50 mL), and the organic layer was washed with 20% aq. sodium chloride (50 mL), dried, and concentrated under reduced pressure. The residue was purified by column chromatography to get **40 [40]** as a light-brown liquid (0.35 g, 65%): ^1^H-NMR (400 MHz, CDCl_3_) *δ* 7.91 (d, *J* = 8.0 Hz, 1H), 7.41 (t, *J* = 7.8 Hz, 1H), 6.76–6.69 (m, 2H), 3.47 (t, *J* = 7.0 Hz, 2H), 3.00 (s, 3H), 2.74 (t, *J* = 7.0 Hz, 2H); ^13^C-NMR (100 MHz, CDCl_3_) *δ* 193.77, 152.76, 135.47, 127.94, 119.83, 117.03, 113.31, 51.35, 39.28, 38.21; FT-IR (Neat) ν_max_2895.45, 1687.52, 821.69cm^−1^; MS: *m*/*z* 162.1 (M + H).

#### 3.7.2. 1-Benzyl-2,3-dihydro-1H-quinolin-4-one (**42**)

A mixture of 2′-fluorophenyl vinyl ketone (0.6 g, 4.03 mmol) and benzylamine (0.58 g, 4.43 mmol) in DMF (15 mL) was stirred at 50 °C for 22 h and monitored by TLC. The reaction mixture was diluted with ethyl acetate (50 mL), and the organic layer was washed with 20% aq. sodium chloride (50 mL), dried, and concentrated under reduced pressure. The residue was purified by column chromatography to get **42** as a white solid (0.65 g, 68%) mp 116–119 °C: ^1^H-NMR (400 MHz, CDCl_3_) *δ* 7.94 (d, *J* = 7.2 Hz, 1H), 7.37–7.25 (m, 6H), 6.74–6.69 (m, 2H), 4.56 (s, 2H), 3.61 (t, *J* = 7.0 Hz, 2H), 2.77 (t, *J* = 7.0 Hz, 2H); ^13^C-NMR (100 MHz, CDCl_3_) *δ* 193.48, 151.74, 137.29, 135.47, 128.86, 128.28, 127.44, 126.80, 119.84, 117.02, 113.44, 55.25, 49.44, 38.07; FT-IR (KBr) ν_max_2902.85, 1663.08, 1501.28, 751.41cm^−1^; MS: *m*/*z* 238.1 (M + H).

### 3.8. Synthesis of Mimosifoliol

#### 3.8.1. 2-Bromo-1,4-dimethoxybenzene (**45**)

To a solution of 1,4-dimethoxybenzene (56 g, 0.47 mol) in acetonitrile (500 mL) was added NBS (72.2 g, 0.47 mmol) at 10 °C, which was then stirred for 12 h at room temperature. The reaction mixture was monitored by TLC, and concentrated under reduced pressure. A crude product was dissolved in methylene chloride (500 mL), washed with water (250 mL × 2), and then dried and concentrated under reduced pressure to get **45** as a light brown syrup (75 g, 85%): ^1^H-NMR (400 MHz, CDCl_3_) *δ* 7.11 (s, 1H), 6.88–6.80 (m, 2H), 3.82 (s, 3H), 3.75 (s, 3H); ^13^C-NMR (100 MHz, CDCl_3_) *δ* 154.06, 150.56, 119.03, 114.66, 113.66, 111.98, 56.85, 55.90; FT-IR (Neat) ν_max_3366.12, 2941.00, 1020.06 cm^−1^; MS: *m*/*z* 218.8 (M + H).

#### 3.8.2. 2,5-Dimethoxy-phenol (**46**)

To a stirred solution of 2-bromo-1,4-dimethoxybenzene (8 g, 36.86 mmol) in water (200 mL) was added cuprous oxide (0.52 g, 3.68 mmol) and sodium hydroxide (48.6 g, 1.2 mol) and stirred for 10 h at 150 °C under hydrogenation. The reaction was monitored by TLC, and the pH was adjusted to 3 with con. HCl, followed by ethyl acetate (150 mL) extraction to get **46 [41]** as a colorless syrup (5.4g, 96%): ^1^H-NMR (400 MHz, CDCl_3_) *δ* 6.77 (d, *J* = 8.8 Hz, 1H), 6.56 (d, *J* = 2.8 Hz, 1H), 6.38 (dd, *J* = 8.8, 2.8 Hz, 1H), 5.72 (s, 1H), 3.83 (s, 3H), 3.73 (s, 3H); ^13^C-NMR (100 MHz, CDCl_3_) *δ* 154.59, 146.51, 141.08, 111.64, 104.28, 101.90, 56.59, 55.64; FT-IR (Neat) ν_max_3430.25, 2974.58, 1114.08 cm^−1^; MS: *m*/*z* 155.2 (M + H).

#### 3.8.3. 3-Dimethylamino-1-phenyl-propan-1-ol (**47**)

To a solution of 3-dimethylamino-1-phenylpropan-1-one (60 g, 0.282 mol) in methanol (300 mL) was added sodium borohydride (10.7 g, 0.282 mol) in 10% aq. NaOH (120 mL) at 10 °C, and the resulting solution was stirred at 10 °C for 3 h and monitored by TLC. The reaction mixture was concentrated under reduced pressure and diluted with ethyl acetate (1.0 Lt). The organic layer was washed with water (0.3 L × 3), and then dried and concentrated under reduced pressure to get (**47**) as a colorless syrup (48 g, 96%): ^1^H-NMR (400 MHz, CDCl_3_) *δ* 7.37–7.29 (m, 4H), 7.23 (t, *J* = 7.0 Hz, 1H), 6.49 (br s, 1H), 4.90 (q, *J* = 3.8 Hz, 1H),2.63–2.57 (m, 1H), 2.46–2.41 (m, 1H), 2.26 (s, 6H), 1.82–1.76 (m, 2H); ^13^C-NMR (100 MHz, CDCl_3_) *δ* 145.19, 128.18, 126.86, 125.60, 75.54, 58.32, 45.31, 34.70; FT-IR (Neat) ν_max_3425.25, 2878.54, 956.25 cm^−1^; MS: *m*/*z* 179.9 (M + H).

#### 3.8.4. (3-Chloro-3-phenyl-propyl)-dimethylamine (**48**)

A solution of 3-dimethylamino-1-phenylpropan-1-ol (5.6 g, 31.3 mmol) in chloroform (100 mL) was purged with HCl until to become acidic at 10 °C. Then, thionyl chloride (8g, 67.5 mmol) was added at 10 °C. The reaction mass temperature was raised to reflux and was stirred for 2 h. The reaction mixture was monitored by TLC and concentrated under reduced pressure. The residue was purified using an acetone (50 mL) slurry to get **48 [42,43]** as a white solid (6.5 g, 89%), DSC. 171.96 °C: ^1^H-NMR (400 MHz, CDCl_3_) *δ* 11.24 (s, 1H), 7.51 (d, *J* = 7.2 Hz, 2H), 7.43–7.34 (m, 3H), 5.33 (q, *J* = 4.6 Hz, 1H), 3.22–3.16 (m, 1H), 3.08–3.02 (m, 1H), 2.73 (s, 6H), 2.61–2.46 (m, 2H); ^13^C-NMR (100 MHz, CDCl_3_) *δ* 141.03, 129.25, 129.17, 127.51, 61.14, 54.75, 42.65, 33.60; FT-IR (KBr) ν_max_3425.25, 2878.54, 956.25 cm^−1^; MS: *m*/*z* 179.9 (M + H).

#### 3.8.5. [3-(2,5-Dimethoxyphenoxy)-3-phenylpropyl]dimethylamine (**49**)

To a stirred solution of (3-chloro-3-phenylpropyl)dimethylamine (**48**) (5 g, 21.4 mmol) and 2,5-dimethoxyphenol (**46**) (3.3 g, 21.4 mmol) in acetone (100 mL) was added K_2_CO_3_ (7.37 g, 53.4 mmol) and stirred for 7 h at reflux temperature. The reaction was monitored by TLC. Salts were removed by filtration and concentrated under reduced pressure to get crude product. The residue was purified by column (only ethyl acetate as eluent) to get **49 [44]** as a white solid (6.2 g, 92%), m.p. 51–53 °C: ^1^H-NMR (400 MHz, CDCl_3_) *δ* 7.39 (d, *J* = 7.6 Hz, 2H), 7.32 (t, *J* = 7.6 Hz, 2H), 7.24 (t, *J* = 7.2 Hz, 1H), 6.77 (d, *J* = 8.4 Hz, 1H), 6.36–6.31 (m, 2H), 5.21 (q, *J* = 3.2 Hz, 1H), 3.82 (s, 3H), 3.60 (s, 3H), 2.43 (t, *J* = 7.2 Hz, 2H), 2.29–2.19 (m, 7H), 2.00–1.95 (m, 1H); ^13^C-NMR (100 MHz, CDCl_3_) *δ* 154.09, 148.85, 144.49, 141.86, 128.51, 127.55, 126.16, 113.54, 104.44, 104.37, 79.93, 57.12, 55.88, 55.47, 45.52, 36.54; FT-IR (KBr) ν_max_ 3428.47, 2935.49, 1509.46, 1230.53 cm^−1^; MS: *m*/*z* 315.9 (M + H).

#### 3.8.6. 2-(3-Dimethylamino-1-phenylpropyl)-3,6-dimethoxyphenol (**50**) and 4-(3-Dimethylamino-1-phenylpropyl)-2,5-dimethoxyphenol (**51**)

To a stirred solution of [3-(2,5-dimethoxyphenoxy)-3-phenylpropyl]dimethylamine (**49**) (7.7 g, 24.4 mmol) in dichloromethane (80 mL) was added perchloric acid 70% (21 g, 146.5 mmol) at 0 °C and maintained for 3 h at the same temperature. Reaction was monitored by TLC. Cold water (150 mL) was added, and the pH was adjusted to 8 with 10% aq. NH_3_ (20 mL). The layers were separated, and the organic layer was dried and concentrated under reduced pressure. The residue was purified by column (only ethyl acetate as eluent) to get **50** & **51 [45]** as white solids (3.04 g and 1.52 g, 60%).

*4-(3-Dimethylamino-1-phenyl-propyl)-2,5-dimethoxy-phenol* (**50**). m.p. 100–101 °C: ^1^H-NMR (400 MHz, CDCl_3_) *δ* 7.25–7.12 (m, 5H), 6.71 (s, 1H), 6.49 (s, 1H), 4.35 (t, *J* = 7.4 Hz, 1H), 3.76 (s, 3H), 3.68 (s, 3H), 2.26–2.13 (m, 10H); ^13^C-NMR (100 MHz, CDCl_3_) *δ* 151.57, 145.07, 144.82, 140.73, 128.20, 127.88, 125.80, 123.87, 111.18, 100.03, 58.27, 56.60, 56.13, 45.42, 41.03, 32.81; FT-IR (KBr) ν_max_3952.54, 2898.58, 1210.58 cm^−1^; HRMS (M)^+^ calculated for C_19_H_25_NO_3_ 315.1834 found 315.1840.

#### 3.8.7. Mimosifoliol (**52**)

To a stirred solution of 4-(3-dimethylamino-1-phenylpropyl)-2,5-dimethoxyphenol (**50**) (1.5 g, 4.75 mmol) in ethylene dichloride (20 mL) was added diisopropylethylamine (0.77 g, 7.13 mmol) followed by ethyl chloroformate (0.92 g, 7.13 mmol) at rt under nitrogen atmosphere. RM was refluxed for 1 h and the reaction was monitored by TLC. The RM was concentrated under reduced pressure. The residue was purified by column (5% ethyl acetate in hexane as eluent) to get mimosifoliol (**52**) as a white solid (1.02 g, 80%), m.p. 73–74 °C: ^1^H-NMR (400 MHz, CDCl_3_) *δ* 7.31–7.20 (m, 5H), 6.59 (s, 1H), 6.45 (s, 1H), 6.37 (t, *J* = 6.0 Hz, 1H), 5.37 (s, 1H), 5.15 (s, 1H), 4.85 (d, *J* = 6.0 Hz, 1H), 4.55 (s, 1H), 3.78 (s, 3H), 3.69 (s, 3H); ^13^C-NMR (100 MHz, CDCl_3_) *δ* 152.2, 144.9, 143.1, 141.0, 140.2, 128.9, 128.8, 126.5, 122.4, 116.6, 113.5, 100.1, 57.4, 56.4, 47.5; FT-IR (KBr) ν_max_3341.93, 2984.56, 1606.55, 1034.03 cm^−1^; HRMS [M]^+^ calculated for C_17_H_18_O_3_: 270.1248 found 270.1251.

### 3.9. Computational Methods

DFT calculations were performed using the B3LYP method, which is capable of predicting molecular structure and properties accurately [46] using the Gaussian 09 program with the B3LYP/6-31++G (d, p) basis set; these are a better choice for small to medium molecules [47]. HOMO, LUMO, and MEP analyses were performed using GaussView v.5.0. RDG, ELF, and LOL analyses were carried out using Multiwfn 3.8 program [48]. The bioactivity of mimosifoliol was calculated using the Molinspiration online tool. Molecular docking calculations were carried out on the aspulvinone dimethylallyltransferase inhibitor by Autodock 4.2 [49].

## 4. Conclusions

In summary, we developed a simple, convenient, and straightforward new synthetic approach for the synthesis of aryl vinyl ketones and mimosifoliol. Compared with procedures that were reported earlier, the present synthetic protocol has several advantages, such as using commercially available and inexpensive materials, with simple and convenient operation. Molecular docking results showed that the binding affinity between ligand and receptor was −5.62 kcal/mol. These values represent an effective inhibition of the aspulvinone dimethylallyltransferase enzyme by the mimosifoliol compound. Furthermore, DFT and ADME predictions for the pharmacodynamics and pharmacokinetic properties revealed the medicinal potential of these molecules for further investigations to obtain some hybrid leads.

## Figures and Tables

**Table 1:** NBO calculations for mimosifoliol.

Donor	Acceptor	E(2)	E_j_ − E_i_	F_(i,j)_
π(C8–C10)	π*(C9–C11)	19.31	0.28	0.066
	π*(C13–C15)	21.23	0.28	0.068
π(C9–C11)	π*(C8–C10)	20.6	0.28	0.068
	π*(C13–C15)	19.94	0.28	0.066
π(C13–C15)	π* (C8–C10)	19.09	0.29	0.066
	π*(C9–C11)	20.09	0.28	0.067
π(C19–C20)	π*(C21–C24)	23.4	0.26	0.072
	π*(C22–C25)	18.09	0.27	0.064
π(C21–C24)	π*(C19–C20)	14.67	0.3	0.061
	π*(C22–C25)	21.9	0.29	0.073
π(C22–C25)	π*(C19–C20)	22.56	0.3	0.073
	π*(C21–C24)	17.01	0.28	0.062
LP(2) O28	π*(C22–C25)	25.77	0.36	0.093
LP(2) O30	π*(C21–C24)	28.58	0.34	0.094

**Table 2:** Atom-condensed Fukui function.

	*f*(−)	*f*(+)	*f*(0)	∆*f* = *f*(+) − *f*(−)
1 C	0.0019	−0.0268	−0.0124	−0.0287
2 H	0.0001	0.0002	0.0002	0.0001
3 C	0.0085	0.0076	0.0081	−0.0009
4 H	0.0004	0	0.0002	−0.0004
5 H	0.0006	0.0077	0.0041	0.0071
6 C	−0.0076	0.0146	0.0035	0.0222
7 H	0.0012	0.0067	0.004	0.0055
8 C	0.0117	−0.084	−0.0362	−0.0957
9 C	0.0084	0.0228	0.0156	0.0144
10 C	0.0083	−0.0139	−0.0028	−0.0222
11 C	0.0073	0.0441	0.0257	0.0368
12 H	0	−0.0268	−0.0134	−0.0268
13 C	0.0014	0.0043	0.0029	0.0029
14 H	0.0004	0	0.0002	−0.0004
15 C	0.0087	0.0071	0.0079	−0.0016
16 H	0	0.0385	0.0193	0.0385
17 H	0.0002	0.0014	0.0008	0.0012
18 H	0.0001	−0.0006	−0.0002	−0.0007
19 C	0.1855	−0.0065	0.0895	−0.192
20 C	0.0056	0.2489	0.1273	0.2433
21 C	0.1321	−0.131	0.0005	−0.2631
22 C	0.2283	−0.081	0.0736	−0.3093
23 H	0.0009	−0.0067	−0.0029	−0.0076
24 C	0.0098	0.3135	0.1617	0.3037
25 C	0.1312	−1.0164	−0.4426	−1.1476
26 H	0	0.5762	0.2881	0.5762
27 O	0.0643	−0.0045	0.0299	−0.0688
28 O	0.0732	−0.0136	0.0298	−0.0868
29 H	0.0007	0.7706	0.3856	0.7699
30 O	0.0973	0.0187	0.058	−0.0786
31 C	−0.0006	−0.0642	−0.0324	−0.0636
32 H	0.0049	0.0169	0.0109	0.012
33 H	0.0007	0.0226	0.0116	0.0219
34 H	−0.0016	0.0617	0.03	0.0633
35 C	0.0025	−0.5114	−0.2544	−0.5139
36 H	0.0065	0.3576	0.182	0.3511
37 H	0.0069	0.2829	0.1449	0.276
38 H	0	0.1628	0.0814	0.1628

**Table 3:** Drug likeness for mimosifoliol.

Compound	MR	HA	TPSA	RB	MW	MlogP	HBA	HBD	Lipinski Violation	Drug Likeness
Mimosifoliol	80.04	20	38.69	5	270.32	3.01	3	1	No	0.55

**Table 4:** Pharmaco-kinetic properties for mimosifoliol through pre-ADMET.

S.No	Compound	BBB	HIA	Caco2	SP	MDCK	PPB
1	Mimosifoliol	1.941	95.842	47.548	−2.136	205.59	96.802

**Table 5:** The bioactivity of mimosifoliol online tool.

S.No	Compound	GPCR	ICM	KI	NR	PI	EI
1	Mimosifoliol	−0.10	−0.16	−0.35	−0.03	−0.40	−0.08

## Data Availability

The presented data are available in this article.

## Supplementary Materials

The following supporting information can be downloaded at: https://www.mdpi.com/article/10.3390/molecules28176214/s1.
