# Supplementary material for: Efficient Approach for the Synthesis of Aryl Vinyl Ketones and Its Synthetic Application to Mimosifoliol with DFT and Autodocking Studies
Source: Molecules. 2023 Aug 24;28(17):6214. doi: 10.3390/molecules28176214 (PMC10488981; doi:10.3390/molecules28176214)
Supplement: Supplementary file 1 [file molecules-28-06214-s001.zip › molecules-2549651-supplementary.pdf]

Supporting information

Efficient Approach for the Synthesis of Aryl Vinyl Ketones and Its Synthetic Application to Mimosifoliol with DFT and Auto-docking Studies

Table of contents

Experimental Procedures and characterization data …………………. S2-S42

1. Experimental Data of **15 to 39** …………………………..………S2-S7
2. ^{1}H and ^{13}C NMR spectra and HRMS data…………………………S9-S47
3. Tables S1-S6 ^{1}H NMR and ^{13}C NMR spectral data: Comparison of predicted and Experimental data...S48-S50

General procedure for synthesis of Mannich base:

To a stirred solution of aromatic methyl ketone (1-13) (1.0 eq) in 2-propanol (3 vol) was added N,N-dimethylamine hydrochloride (1.25 eq), paraformaldehyde (1.5 eq) and 50% aqueous hydrochloride (5 mL) at room temperature. The reaction mixture was stirred at 85°C for 25 hours. After completion of the starting material by TLC, reaction mixture was cooled to 5-10°C and stirred for 1 hour. The observed solid was filtered and washed with chilled 2-propanol to obtained the Mannich base compound (15-26)

1-(2-Chlorophenyl)-3-dimethylaminopropan-1-one hydrochloride **(15).**

15 as a white solid (94%), mp 162.2–164.7 °C:^{1}H NMR (400 MHz, MeOD)δ7.79 (d, J = 7.6 Hz, 1H), 7.75 (t, J = 5.0 Hz, 2H), 7.49-7.45 (m, 1H), 3.64-3.58 (m, 4H), 2.98 (s, 6H); ^{13}C NMR (100 MHz, MeOD)δ198.78, 137.16, 132.66, 130.89, 130.61, 129.54, 127.10, 52.59, 42.55, 36.88; FT-IR (KBr) ν_{max}3403.20, 1711.65, 1326.82, 771.22 cm^{-1}; MS: m/z 212.0 (M + H).

1-(2,4-Dichlorophenyl)-3-dimethylaminopropan-1-one hydrochloride **(17).**

17 as a white solid (80%), mp 133.6–136.2 °C:^{1}H NMR (400 MHz, DMSO-d_6)δ10.85 (br s, 1H), 7.90 (d, J = 8.4 Hz, 1H), 7.78 (s, 1H), 7.64(d, J = 6.8 Hz, 1H), 3.58 (t, J = 7.2 Hz, 2H), 3.40 (t, J = 9.0 Hz, 2H), 2.77 (s, 6H); ^{13}C NMR (100 MHz, DMSO-d_6)δ 197.99, 137.24, 136.27, 131.83, 131.79, 130.73, 128.13, 51.71, 42.62, 37.26; FT-IR (KBr) ν_{max}3433.12, 2676.69, 1697.37, 1582.47, 964.22 cm^{-1}; MS: m/z 246.0 (M + H).

1-(2,4-Dichloro-5-fluoro-phenyl)-3-dimethylamino-propan-1-one hydrochloride **(18).**

18 as a white solid (67%), mp 145.2–148.4 °C:^{1}H NMR (400 MHz, DMSO-d_6)δ11.19 (br s, 1H), 8.08 (d, J = 9.6 Hz, 1H), 7.96 (d, J = 6.4 Hz, 1H), 3.61 (t, J = 7.2 Hz, 2H), 3.38 (t, J = 8.4 Hz,

2H), 2.77 (s, 6H); ^{13}C NMR (100 MHz, DMSO-d_6) δ 196.96, 157.60, 155.13, 137.59, 137.53, 132.76, 126.62, 126.58, 124.16, 123.97, 118.63, 118.38, 51.50, 42.58, 37.25; FT-IR (KBr) ν_{max} 1670.00, 1471.32, 1041.09, 717.88 cm^{-1}; MS: m/z 264.0 (M + H).

3-Dimethylamino-1-thiophen-2-yl-propan-1-one hydrochloride **(19).**

19 as a white solid (77%), mp 172.8–175.1 °C: ^{1}H NMR (400 MHz, MeOD) δ 8.03 (d, J = 3.6 Hz, 1H), 7.94 (d, J = 4.8 Hz, 1H), 7.27 (t, J = 4.2 Hz, 1H), 3.63-3.55 (m,4H), 2.97 (s, 6H); ^{13}C NMR (100 MHz, MeOD) δ 189.72, 142.49, 134.91, 133.66, 128.33, 52.76, 42.51, 33.17; FT-IR (KBr) ν_{max} 1678.32, 1487.52, 1058.54, 725.25 cm^{-1}; MS: m/z 184.0 (M + H).

3-Dimethylamino-1-furan-2-yl-propan-1-one hydrochloride **(20).**

20 as a white solid (76%), mp 178.2–180.8 °C: ^{1}H NMR (400 MHz, MeOD) δ 7.86 (s, 1H), 7.49 (d, J = 3.6 Hz, 1H), 6.72 (t, J = 1.6 Hz, 1H), 3.58 (t, J = 6.2 Hz,2H), 3.50 (t, J = 6.2 Hz, 2H), 2.96 (s, 6H); ^{13}C NMR (100 MHz, MeOD) δ 185.25, 151.64, 147.82, 118.62, 112.39, 52.37, 42.49, 32.48; FT-IR (KBr) ν_{max} 1660.60, 1473.13, 962.91, 797.24 cm^{-1}; MS: m/z 168.0 (M + H).

3-Dimethylamino-1-(4-phenoxyphenyl)-propan-1-one hydrochloride **(21).**

21 as a white solid (69%), mp 134.2–137.7 °C: ^{1}H NMR (400 MHz, MeOD) δ 8.09 (d, J = 8.8 Hz, 2H), 7.47 (t, J = 7.8 Hz, 2H), 7.27 (t, J = 7.4 Hz,1H), 7.11 (d, J = 8.0 Hz, 2H), 7.06 (d, J = 8.4 Hz, 2H), 3.63-3.56 (m, 4H), 2.97 (s, 6H); ^{13}C NMR (100 MHz, MeOD) δ 195.52, 162.81, 155.28, 130.48, 130.40, 129.95, 124.68, 120.02, 116.85, 53.07, 42.52, 32.62; FT-IR (KBr) ν_{max} 1715.25, 1458.87, 985.65, 787.58 cm^{-1}; MS: m/z 270.1 (M + H).

3-Dimethylamino-1-(2-hydroxy-phenyl)-propan-1-one hydrochloride **(22).**

22 as a white solid (71%), mp 174.8–177.3 °C:^{1}H NMR (400 MHz, DMSO-d_6) δ11.50 (s, 1H), 10.99 (br s, 1H), 7.88 (d, J = 8.0 Hz, 1H), 7.54 (t, J = 7.4 Hz, 1H), 7.08 (d,J = 8.0 Hz,1H), 6.99 (t, J = 7.4 Hz, 1H), 3.69 (t, J = 7.2 Hz, 2H), 3.42 (t, J = 7.2 Hz, 2H), 2.79 (s, 6H); ^{13}C NMR (100 MHz, DMSO-d_6)δ201.33, 160.46, 136.40, 130.89, 121.43, 119.66, 118.18, 51.99, 42.63, 35.51; FT-IR (KBr) ν_{max}3419.95, 2964.51, 1649.13, 752.32 cm^{-1}; MS: m/z 194.1 (M + H).

3-Dimethylamino-1-naphthalen-2-yl-propan-1-one hydrochloride **(23).**

23 as a white solid (82%), mp 158.5–162.8 °C:^{1}H NMR (400 MHz, DMSO-d_6) δ11.17 (br s, 1H), 8.79 (s, 1H), 8.17 (d, J = 8.0 Hz, 1H), 8.07–8.01 (m, 3H), 7.72–7.64 (m, 2H), 3.84 (t, J = 7.4 Hz, 2H), 3.50 (t, J = 7.4 Hz, 2H), 2.86 (s, 6H); ^{13}C NMR (100 MHz, DMSO-d_6)δ197.08, 135.70, 133.69, 132.58, 130.66, 130.04, 129.34, 128.86, 128.20, 127.57, 123.82, 52.25, 42.60, 33.71; FT-IR (KBr) ν_{max}3485.68, 2705.17, 1675.57, 1468.94, 798.54 cm^{-1}; MS: m/z 228.1 (M + H).

3-Dimethylamino-1-naphthalen-1-yl-propan-1-one hydrochloride **(24).**

24 as a white solid (65%), mp 155.3–157.8 °C:^{1}H NMR (400 MHz, DMSO-d_6) δ11.19 (br s, 1H), 8.61 (d, J = 8.4 Hz, 1H), 8.26 (d, J = 6.8 Hz, 1H), 8.21 (d, J = 8.0 Hz, 1H), 8.05 (d, J = 7.6Hz, 1H), 7.68–7.59 (m, 3H), 3.81 (t, J = 7.4 Hz, 2H), 3.50 (t, J = 7.0 Hz, 2H), 2.84 (s, 6H); ^{13}C NMR (100 MHz, DMSO-d_6)δ200.81, 134.50, 133.96, 133.71, 129.83, 129.50, 129.06, 128.50, 126.99, 125.79, 125.27, 52.37, 42.64, 36.52; FT-IR (KBr) ν_{max}1682.57, 1322.38, 777.98 cm^{-1}; MS: m/z 228.1 (M + H).

3-(dimethylamino)-1-(p-tolyl)propan-1-one hydrochloride **(25).**

25 as a white solid (64%), mp 151.3–154.9°C:^{1}H NMR (400 MHz, DMSO-d_6) δ10.74 (br s, 1H), 7.93 (d, J = 8.0 Hz, 2H), 7.38 (d, J = 8.0 Hz, 2H), 3.61 (t, J = 7.2 Hz, 2H), 3.40 (t, J = 7.2 Hz, 2H), 2.79 (s, 6H), 2.39 (s, 3H); ^{13}C NMR (100 MHz, DMSO-d_6)δ196.68, 144.62, 133.98, 129.79, 128.60, 52.21, 42.55, 33.47, 21.65; FT-IR (KBr) ν_{max}1675.37, 963.44, 787.21 cm^{-1}; MS: m/z 192.2 (M + H).

1-(4-Chloro-phenyl)-3-dimethylamino-propan-1-one hydrochloride **(26).**

26 as a white solid (74%), mp 168–171°C:^{1}H NMR (400 MHz, DMSO-d_6) δ11.13 (br s, 1H), 8.04 (d, J = 8.4 Hz, 2H), 7.65 (d, J = 8.0 Hz, 2H), 3.68 (t, J = 7.4 Hz, 2H), 3.40 (s,3H), 2.80 (s, 6H); ^{13}C NMR (100 MHz, DMSO-d_6)δ196.21, 139.04, 135.11, 130.41, 129.38, 52.01, 42.56, 33.70; FT-IR (KBr) ν_{max}1684.76, 1220.54, 967.47, 796.17cm^{-1}; MS: m/z 212.1 (M + H).

General procedure for synthesis of Aryl Vinyl Ketones:

To a stirred solution of Mannich base (14-26) (1.0 eq) in 1,2-Dichloro ethane (8 vol) was added diisopropylethylamine (1.5 eq) followed by ethyl chloroformate (1.5 eq) at room temperature. The resulting reaction mixture was refluxed for 5 hours under a nitrogen atmosphere. After cooling to room temperature, the solvent was evaporated. Purification of the residue by column chromatography (silica gel, only hexane) provided aryl vinyl ketones (27-39)

1-(2-Fluoro-phenyl)-propenone **(29).**

29 as a color less syrup (97%): ^{1}H NMR (400 MHz, $CDCl_3$) δ7.74-7.70 (m, 1H), 7.51-7.46 (m, 1H), 7.22 (t, J = 7.4 Hz, 1H), 7.13 (t, J = 9.4 Hz, 1H), 7.03-6.96 (m, 1H), 6.38 (d,J = 17.2 Hz, 1H), 5.90 (d,J = 10.4 Hz, 1H); ^{13}C NMR (100MHz, $CDCl_3$) δ189.37,189.34, 162.44, 159.92, 135.47, 135.41, 134.14, 134.05, 130.84, 130.81, 130.04, 126.25, 126.12, 124.42, 124.39, 116.56, 116.33; FT-IR (Neat) ν_{max}1671.37, 1611.79, 1401.33, 999.64, 759.36 cm^{-1}; MS: m/z 151.1 (M + H).

1-(2,4-Dichlorophenyl)propenone **(30).**

30 as a color less syrup (77%): ^{1}H NMR (400 MHz, $CDCl_3$) δ7.44 (s, 1H), 7.38 (d, J = 8.0 Hz, 1H), 7.33(d, J = 7.2 Hz, 1H), 6.78 (dd, J = 17.2, 10.4 Hz, 1H), 6.17 (d,J = 17.2 Hz, 1H), 6.07 (d,J = 10.4 Hz, 1H); ^{13}C NMR (100MHz, $CDCl_3$) δ192.85, 137.08, 136.47, 135.79, 132.41, 132.15, 130.41, 130.18, 127.18; FT-IR (Neat) ν_{max}1673.80, 1404.18, 995.89, 815.89 cm^{-1}; MS: m/z 202.9 (M + H).

1-(2,4-Dichloro-5-fluorophenyl)propenone **(31).**

31 as a color less syrup (83%): ^{1}H NMR (400 MHz, $CDCl_3$) δ7.51 (d, J = 6.4 Hz, 1H), 7.24 (d, J = 8.4 Hz, 1H), 6.79 (dd, J = 17.6, 10.4 Hz, 1H), 6.21 (d,J = 17.6 Hz, 1H), 6.11 (d,J = 10.4 Hz, 1H); ^{13}C NMR (100MHz, $CDCl_3$) δ191.51, 157.91, 155.40, 137.79, 137.73, 135.32, 132.70, 132.03, 126.91, 126.87, 124.36, 124.17, 117.41, 117.18 ; FT-IR (Neat) ν_{max}1684.20, 985.28, 779.54 cm^{-1}; MS: m/z 219.1 (M +H).

1-Furan-2-yl-propenone **(33).**

33 as a color less syrup (94%): ^{1}H NMR (400 MHz, $CDCl_3$) δ7.38 (s, 1H), 7.00 (d, J = 3.6 Hz, 1H), 6.80 (dd, J = 17.2, 10.8 Hz, 1H), 6.26 (d,J = 2.0 Hz, 1H), 6.21 (d,J = 16.4 Hz, 1H), 5.54 (d,J = 10.0 Hz, 1H); ^{13}C NMR (100MHz, $CDCl_3$) δ177.43, 152.61, 146.95, 131.17, 128.86, 118.25, 112.29; FT-IR (Neat) ν_{max}3130.25, 1671.49, 1465.11, 771.77cm^{-1}; MS: m/z 123.0 (M +H).

1-(4-Phenoxy-phenyl)-propenone **(34).**

34 as a color less syrup (82%): ^{1}H NMR (400 MHz, $CDCl_3$) δ7.94 (d, J = 8.8 Hz, 2H), 7.38 (t, J = 7.8 Hz, 2H), 7.19-7.7.09 (m, 2H), 7.06 (d, J = 8.0 Hz, 2H), 7.00 (d,J = 8.8 Hz, 2H), 6.44 (d,J =

17.2, 1.2 Hz, 1H), 5.86 (d,J = 10.4, 0.8 Hz, 1H); ^{13}C NMR (100MHz, $CDCl_3$) δ189.17, 162.03, 155.45, 132.08, 131.85, 131.03, 130.10, 129.65, 124.69, 120.22, 117.40; FT-IR (Neat) ν_{max}1651.52, 1413.94, 1243.92, 764.02 cm^{-1}; MS: m/z 225.0 (M +H).

1-(p-tolyl)prop-2-en-1-one **(35)**

35 as a color less syrup (77%): ^1H NMR (400 MHz, $CDCl_3$) δ12.52 (s, 1H), 7.79 (d, J = 8.0 Hz, 1H), 7.49 (t, J = 7.6 Hz, 1H), 7.33 (dd, J = 16.8, 10.4 Hz, 1H), 7.00 (d,J = 8.4 Hz, 1H), 6.92 (t,J = 7.6 Hz, 1H), 6.57 (dd, J = 16.8,1668 0.8 Hz, 1H), 5.96 (dd,J = 10.4, 0.8 Hz, 1H); ^{13}C NMR (100MHz, $CDCl_3$) δ194.38, 163.59, 136.67, 130.78, 130.60, 130.08, 119.38, 118.96, 118.56; FT-IR (Neat) ν_{max}3280.58, 1780.52, 1387.24, 810.58cm^{-1}; MS: m/z 149.0 (M +H).

1-Naphthalen-1-yl-propenone **(37).**

37 as a color less syrup (83%): ^1H NMR (400 MHz, $CDCl_3$)δ8.34 (d, J = 8.4 Hz, 1H), 7.98 (d, J = 8.4 Hz, 1H), 7.89 (d, J = 8.0 Hz, 1H), 7.72 (d, J = 7.2 Hz, 1H), 7.57-7.47 (m, 3H), 6.97 (dd,J = 17.6, 10.8 Hz, 1H), 6.27 (d, J = 17.2 Hz, 1H), 6.04 (d,J = 10.4 Hz, 1H); ^{13}C NMR (100MHz, $CDCl_3$) δ195.91, 136.94, 135.76, 133.86, 132.01, 131.42, 130.56, 128.45, 127.79, 127.56, 126.51, 125.63, 124.36; FT-IR (Neat) ν_{max}3334.22, 1668.66, 1404.41, 1026.72, 786.56 cm^{-1}; MS: m/z 183.1 (M +H).

1-Naphthalen-1-yl-propenone **(38).**

38 as a color less syrup (71%): ^1H NMR (400 MHz, $CDCl_3$)δ7.85 (d, J = 8.0 Hz, 2H), 7.26 (d, J = 8.0 Hz, 2H), 7.15 (dd,J = 17.2, 10.4 Hz, 1H), 6.43 (d, J = 16.8Hz, 1H), 5.88 (d,J = 10.0 Hz, 1H), 2.39 (s, 3H); ^{13}C NMR (100MHz, $CDCl_3$) δ190.42, 143.83, 134.75, 132.36, 129.32, 128.83,

21.63; FT-IR (Neat) ν_{max}3031.97, 2923.33, 1667.81, 1606.39, 1002.25, 771.34cm^{-1}; MS: m/z 147.1 (M +H).

1-(4-Chloro-phenyl)-propenone **(39).**

39 as a color less syrup (82%): ^{1}H NMR (400 MHz, $CDCl_3$)δ 7.88 (d, *J* = 8.4 Hz, 2H), 7.44 (d, *J* = 8.8 Hz, 2H), 7.14 (dd,*J* = 17.2, 10.4 Hz, 1H), 6.43 (d, *J* = 16.8 Hz, 1H), 5.94 (d,*J* = 11.2 Hz, 1H); ^{13}C NMR (100MHz, $CDCl_3$) δ189.51, 148.48, 139.37, 135.53, 131.87, 130.48, 130.04, 128.89; FT-IR (Neat) ν_{max}2985.65, 1672.56, 958.47cm^{-1}; MS: m/z 167.1 (M +H).

3-Dimethylamino-1-phenylpropan-1-one hydrochloride (**14**)

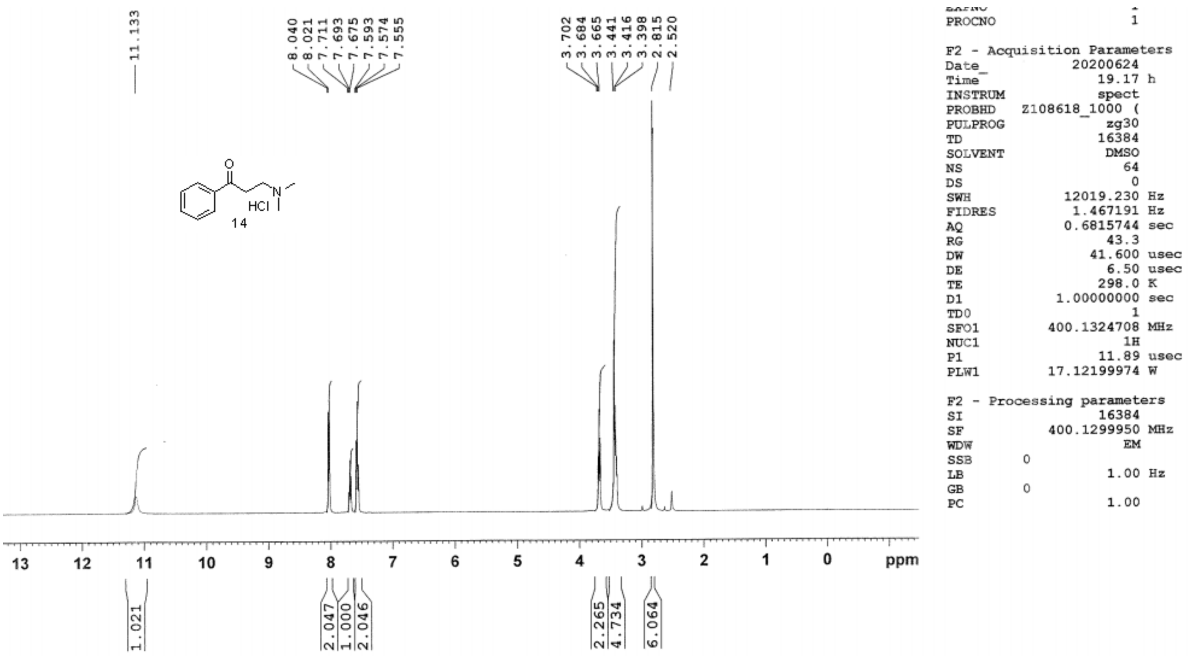

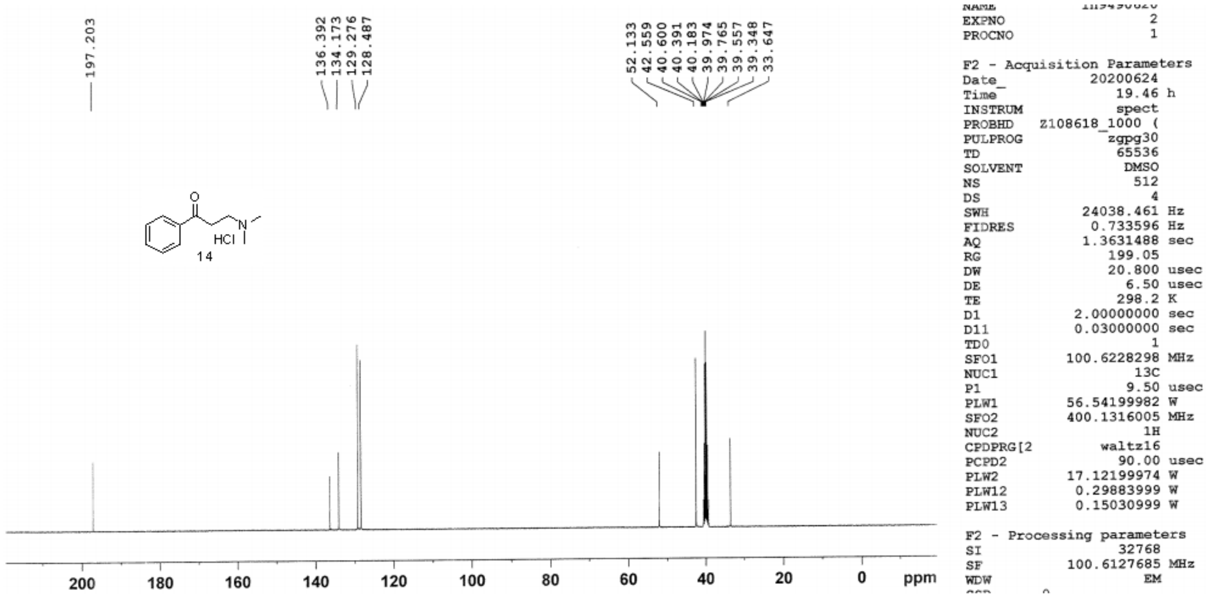

1-Phenyl-propenone **(27).**

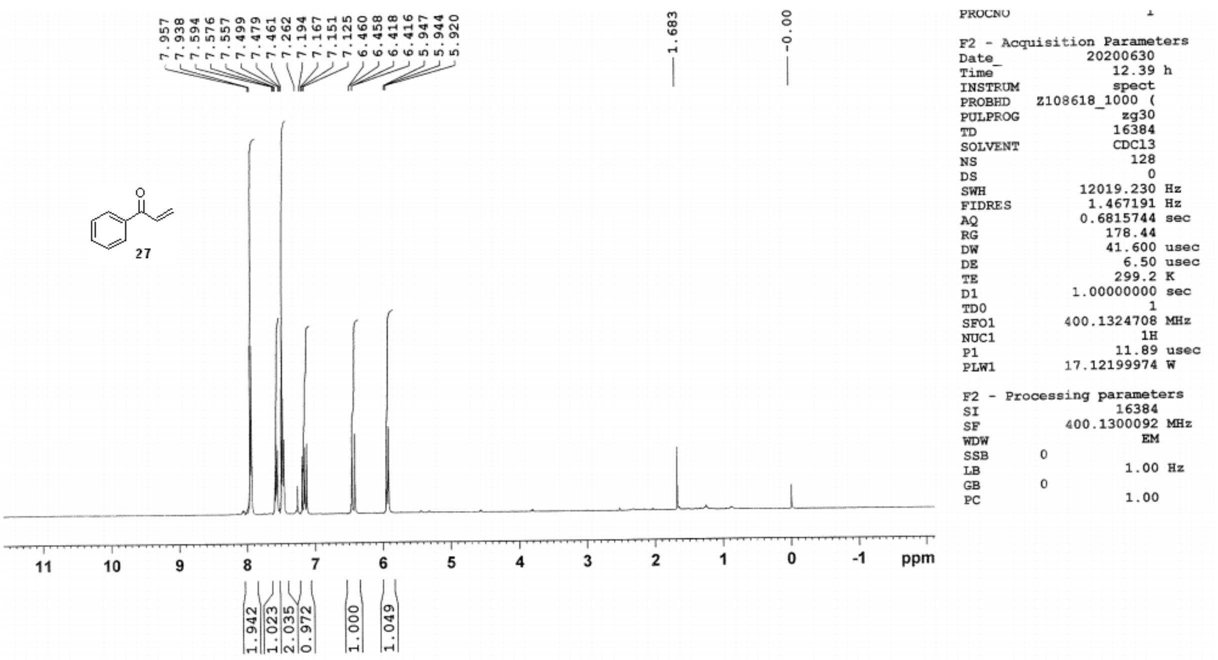

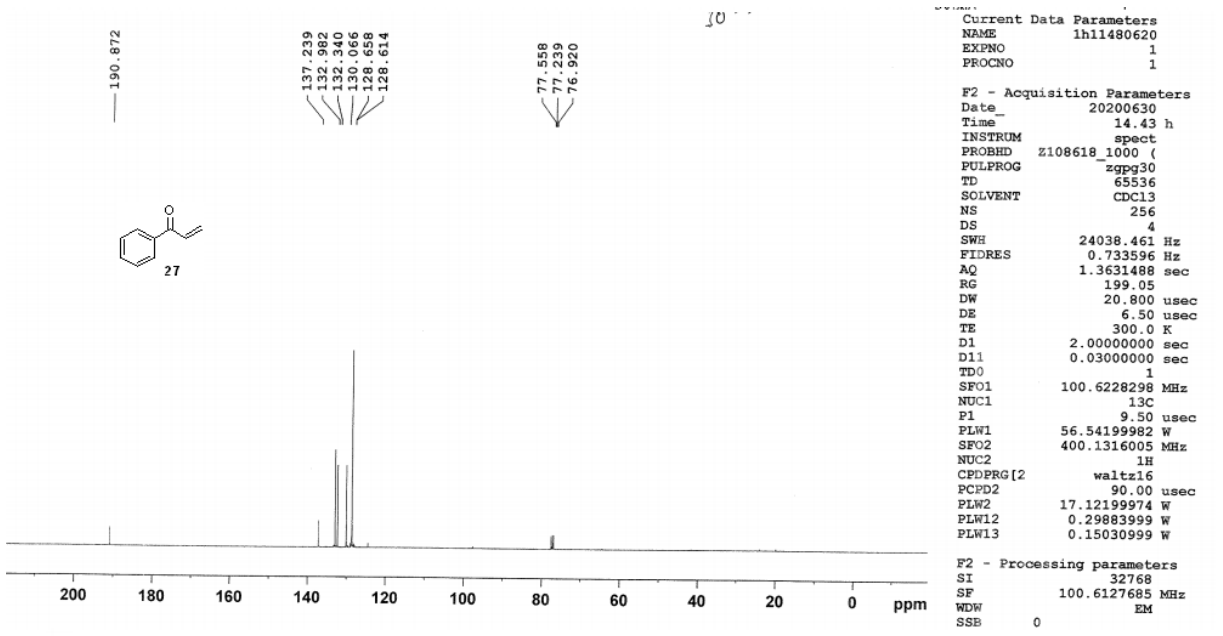

1-(2-Chlorophenyl)-3-dimethylaminopropan-1-one hydrochloride **(15)**

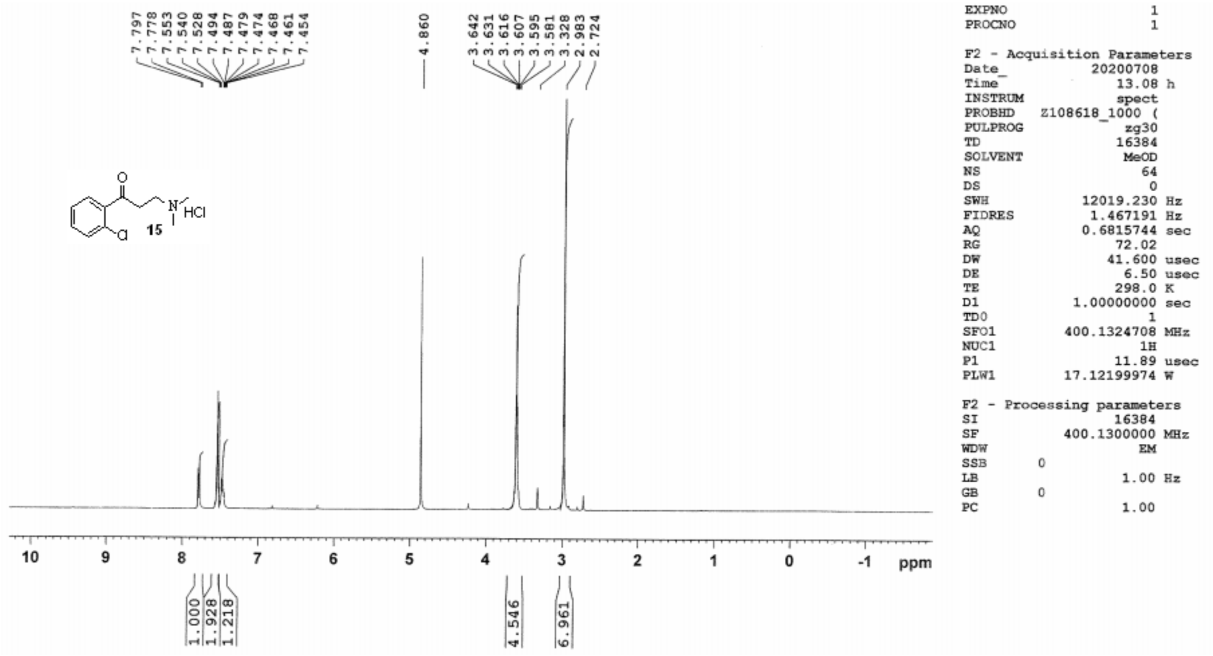

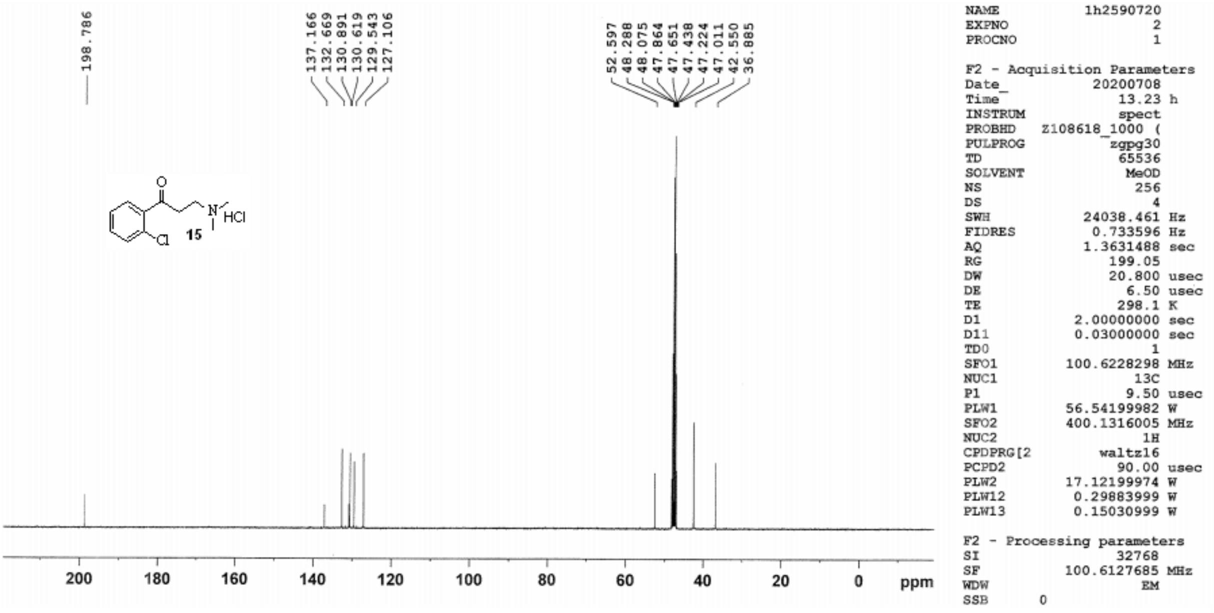

1-(2-Chloro-phenyl)propenone **(28).**

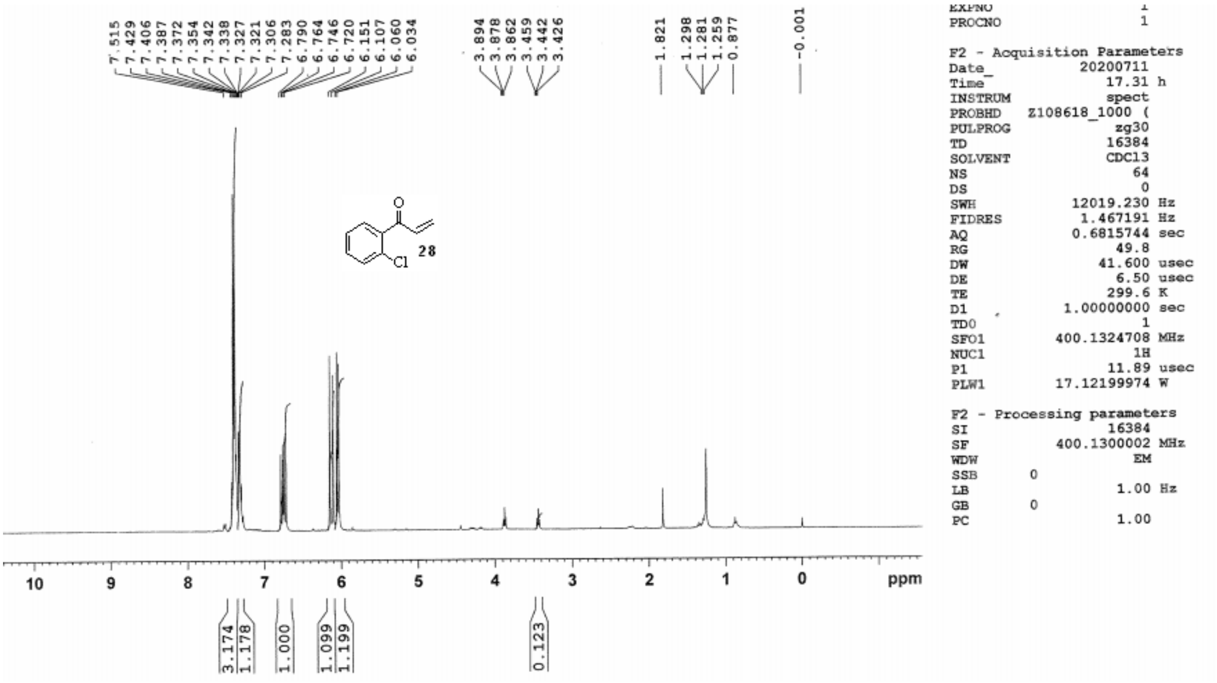

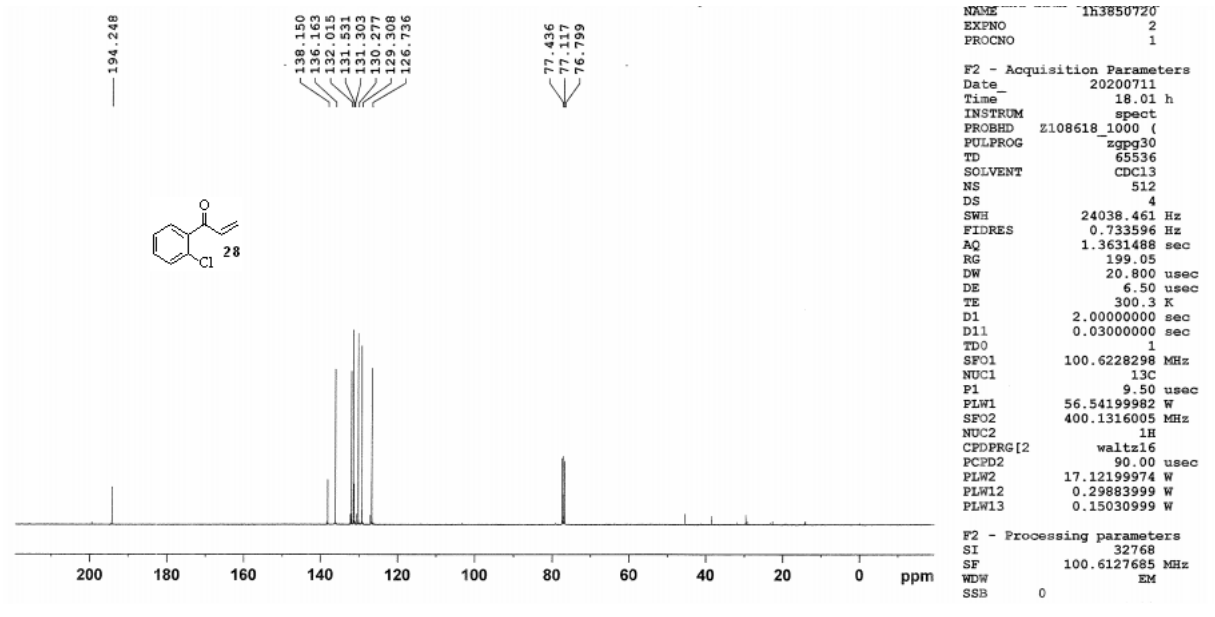

3-Dimethylamino-1-(2-fluorophenyl)propan-1-one hydrochloride **(16)**

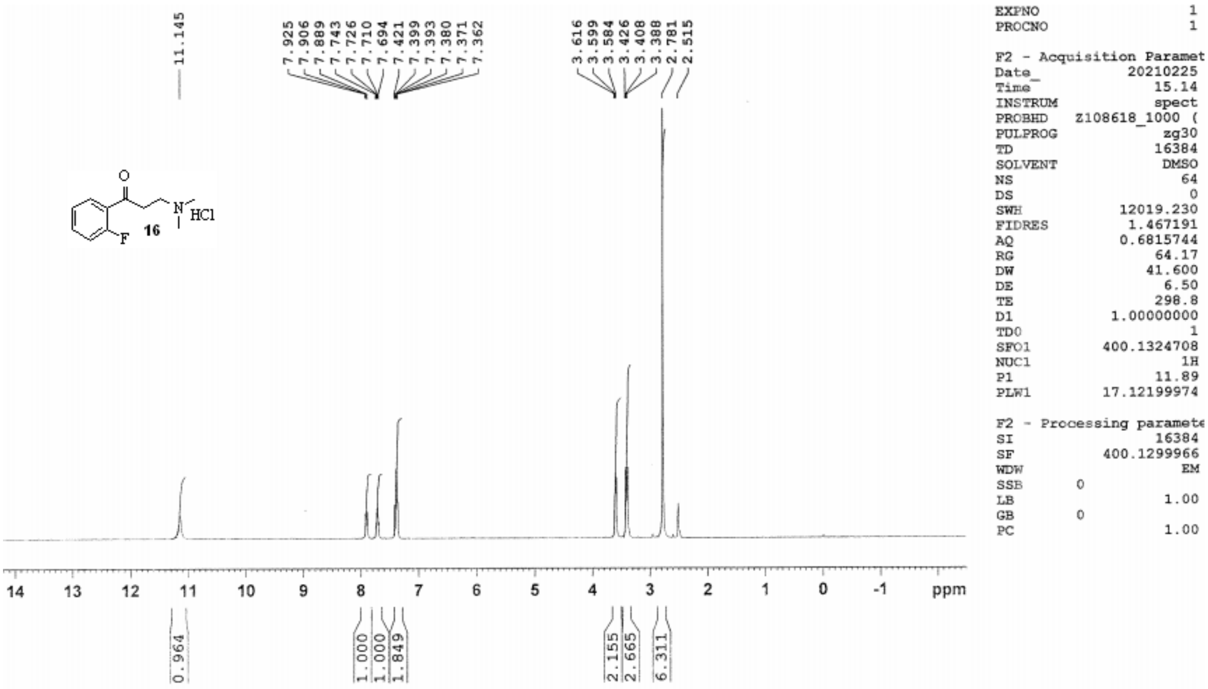

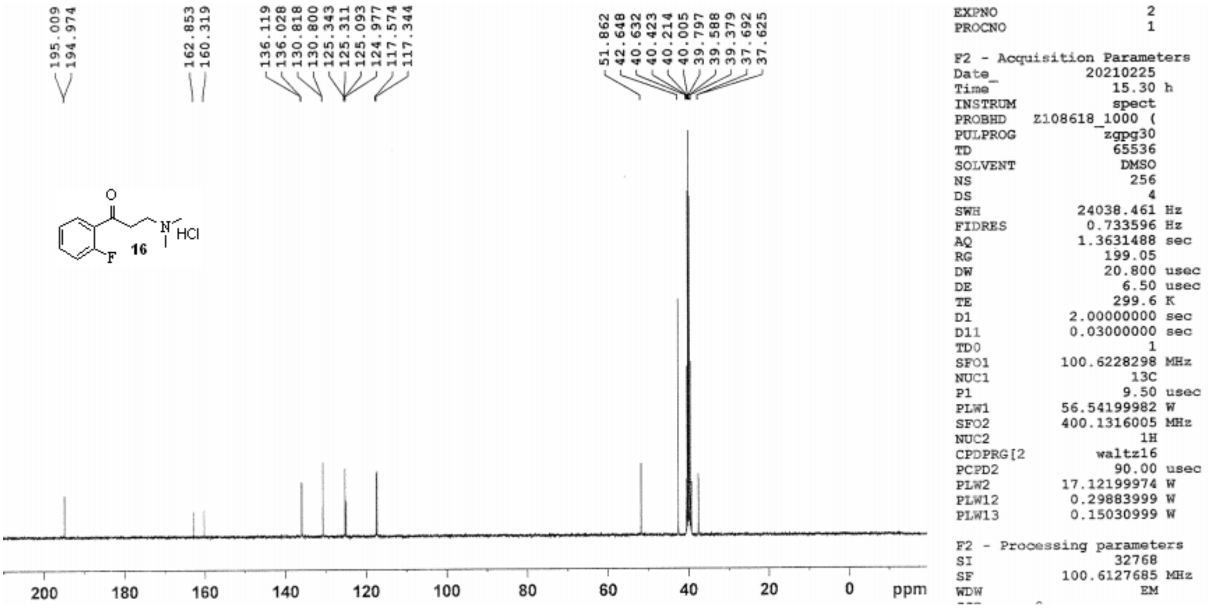

1-(2-Fluoro-phenyl)-propenone **(29)**

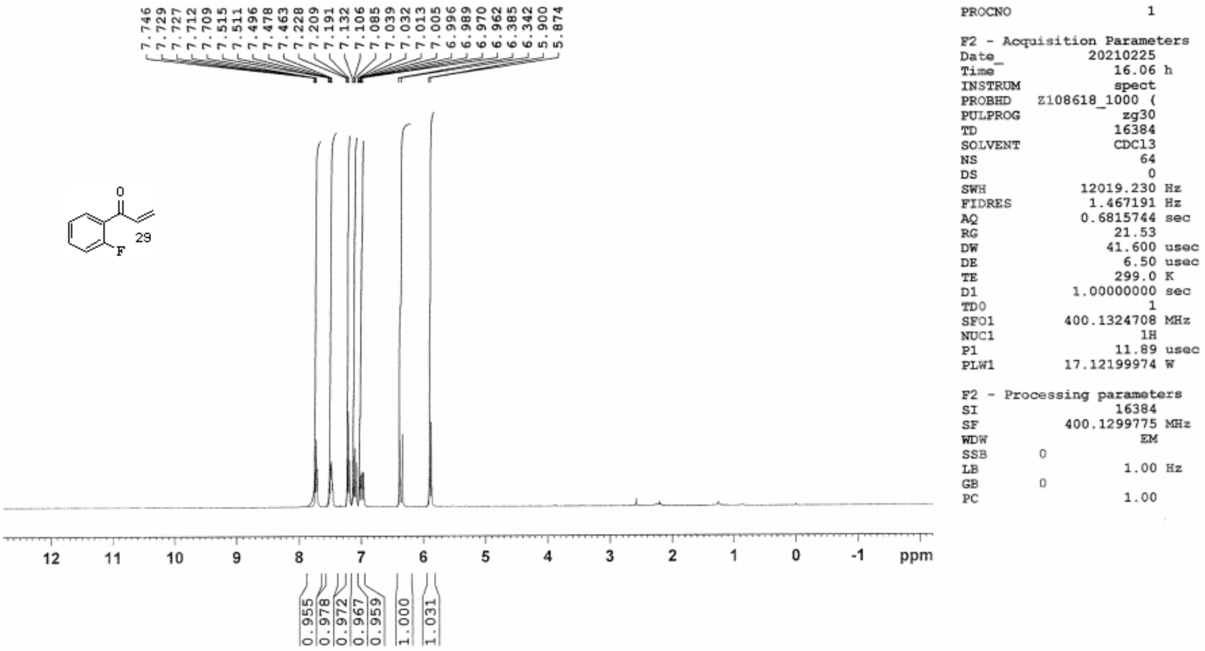

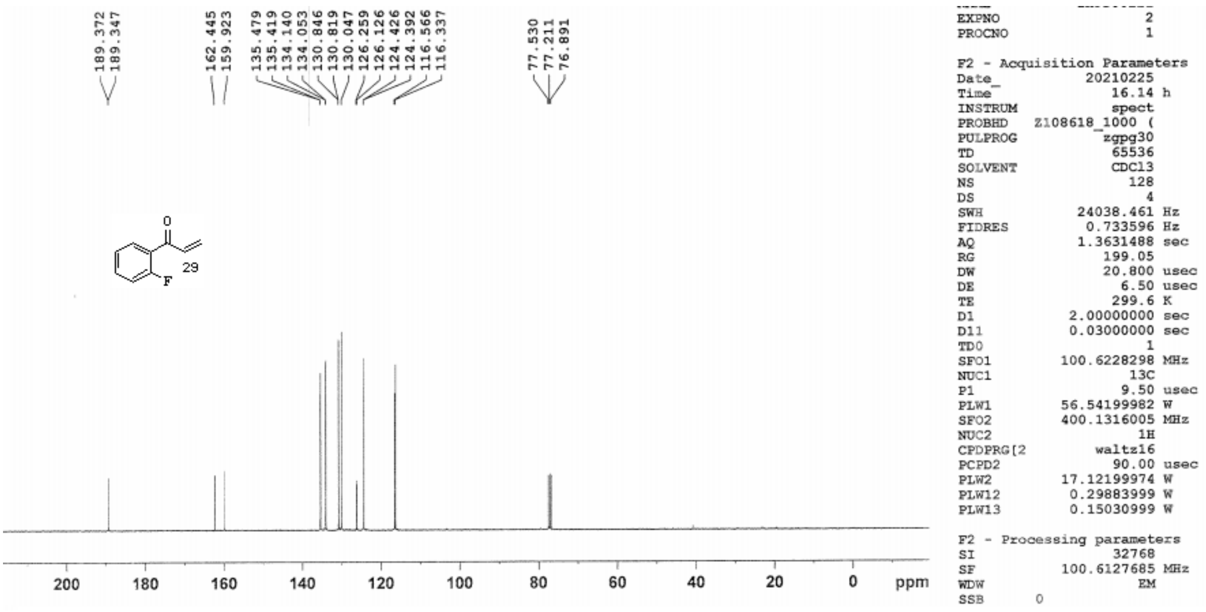

1-(2,4-Dichlorophenyl)-3-dimethylaminopropan-1-one hydrochloride **(17)**

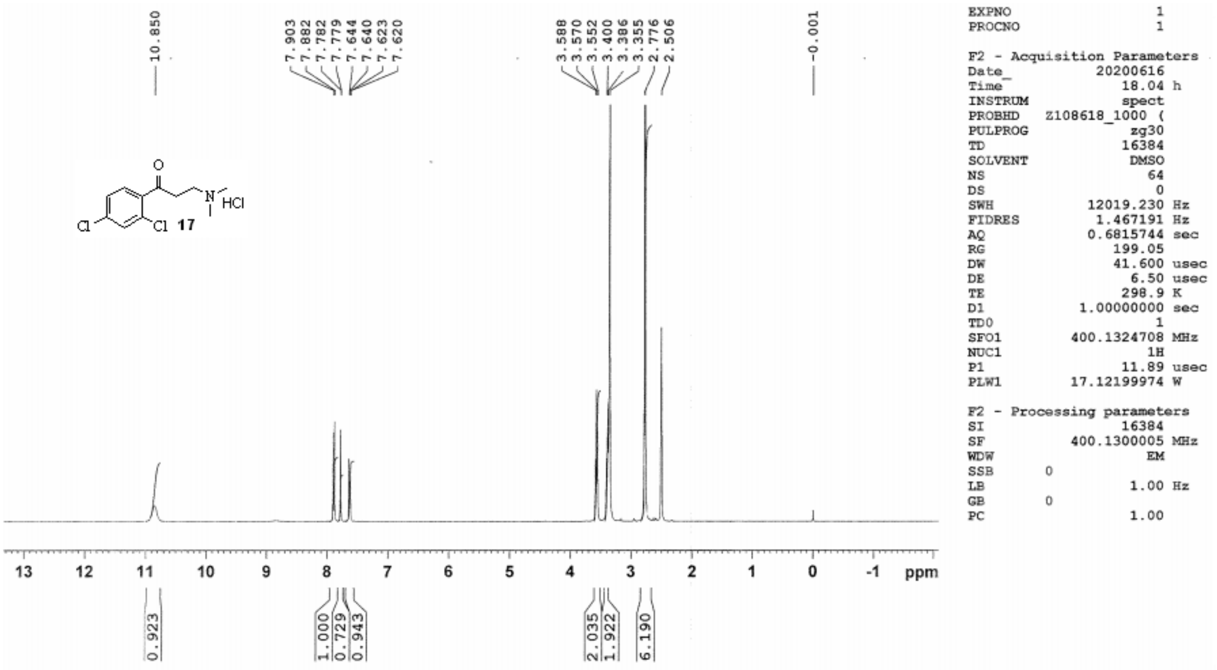

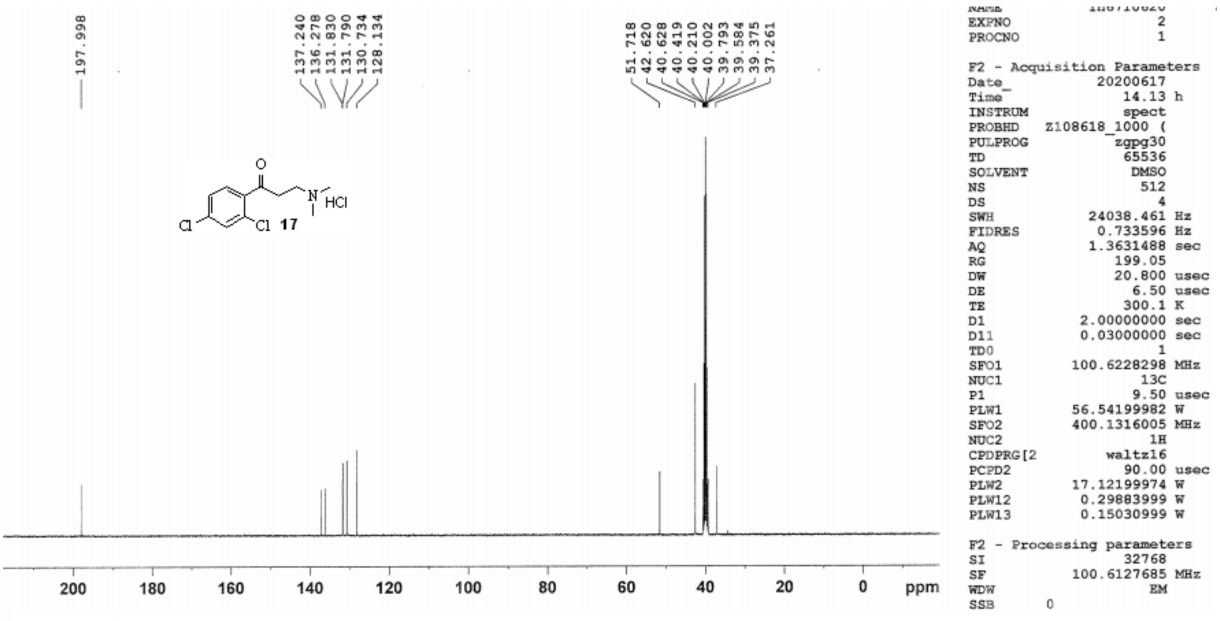

1-(2,4-Dichlorophenyl)propenone **(30)**

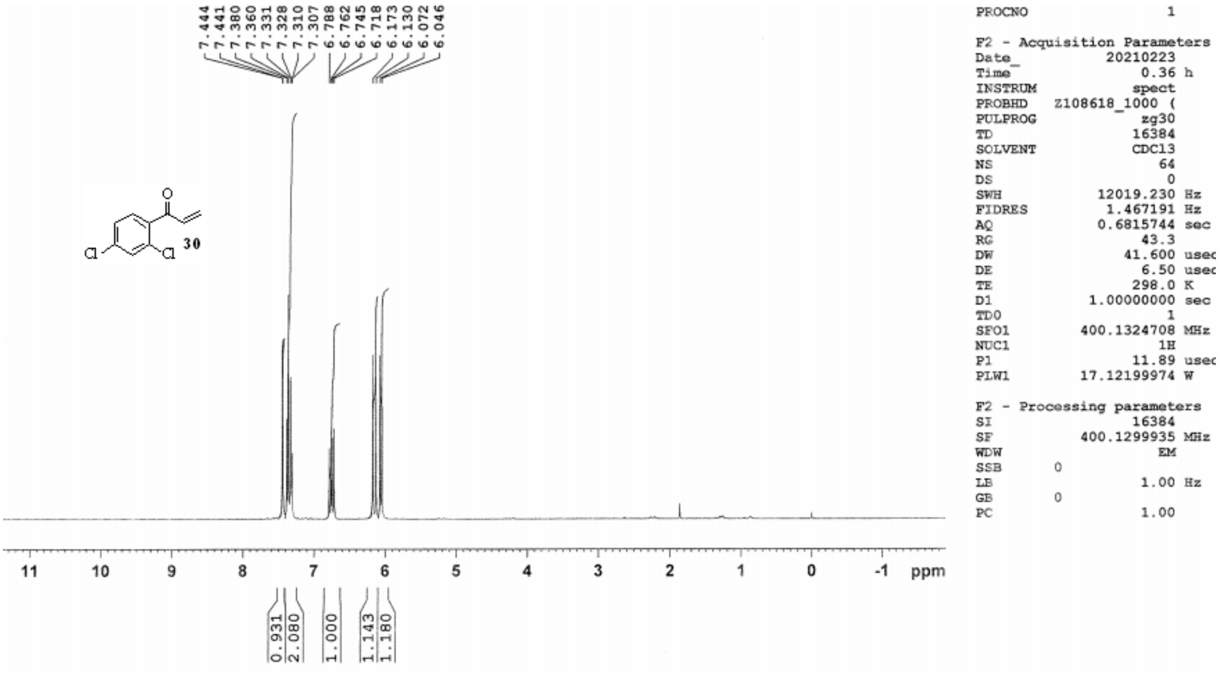

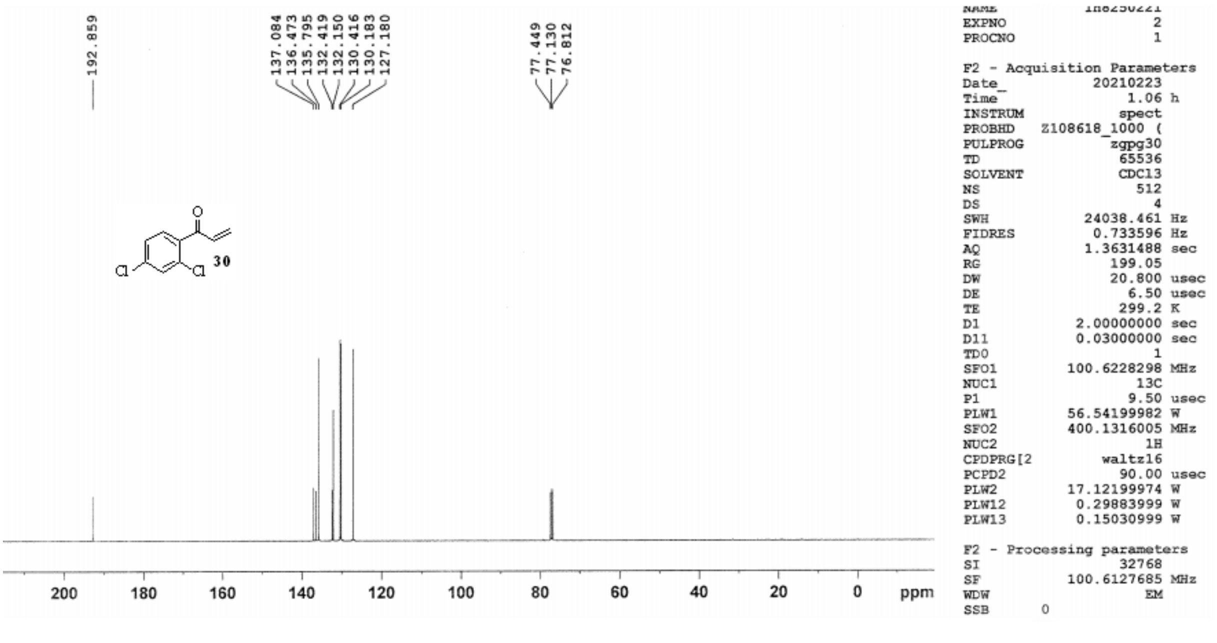

1-(2,4-Dichloro-5-fluoro-phenyl)-3-dimethylamino-propan-1-one hydrochloride **(18)**

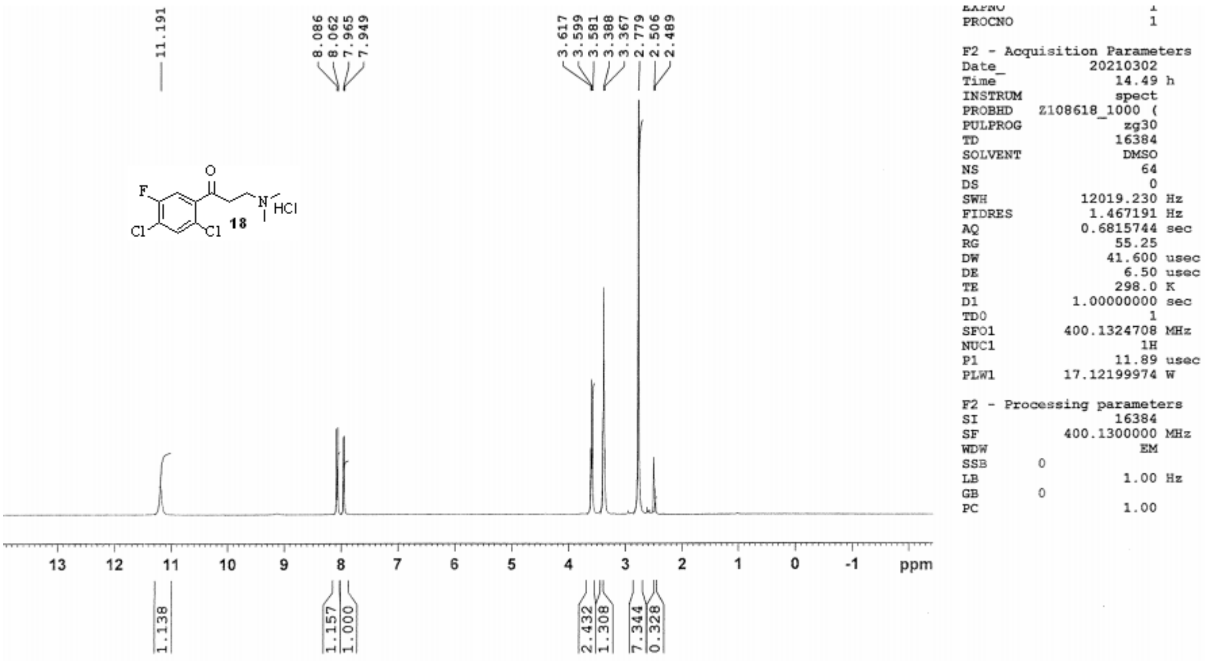

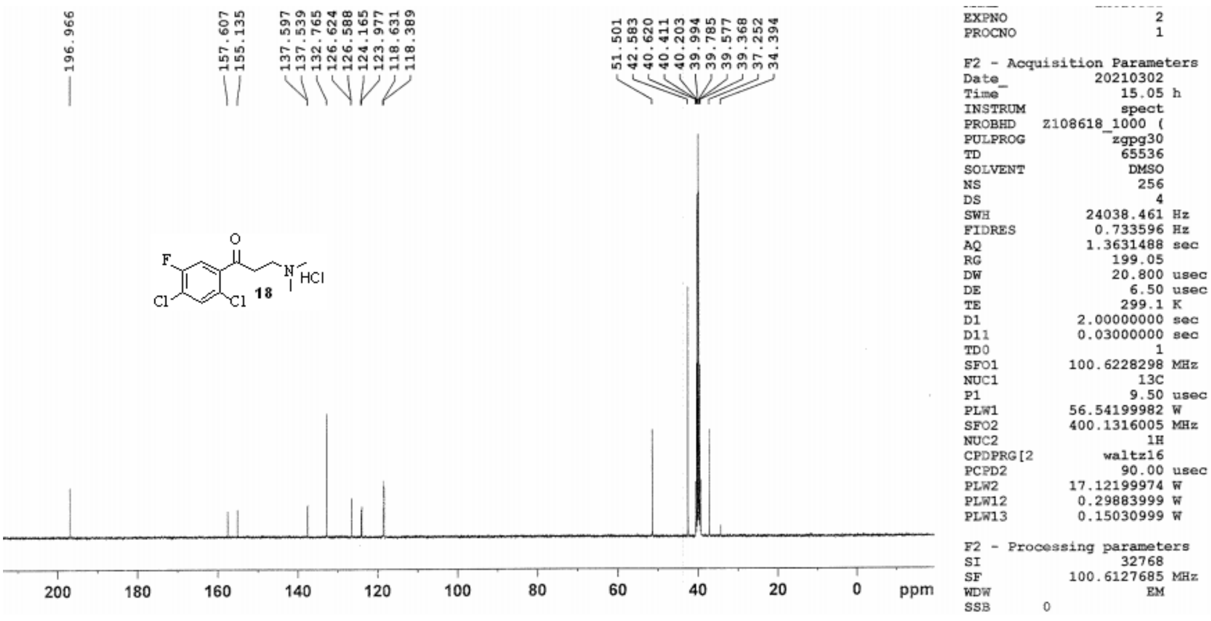

1-(2,4-Dichloro-5-fluorophenyl)propenone **(31)**

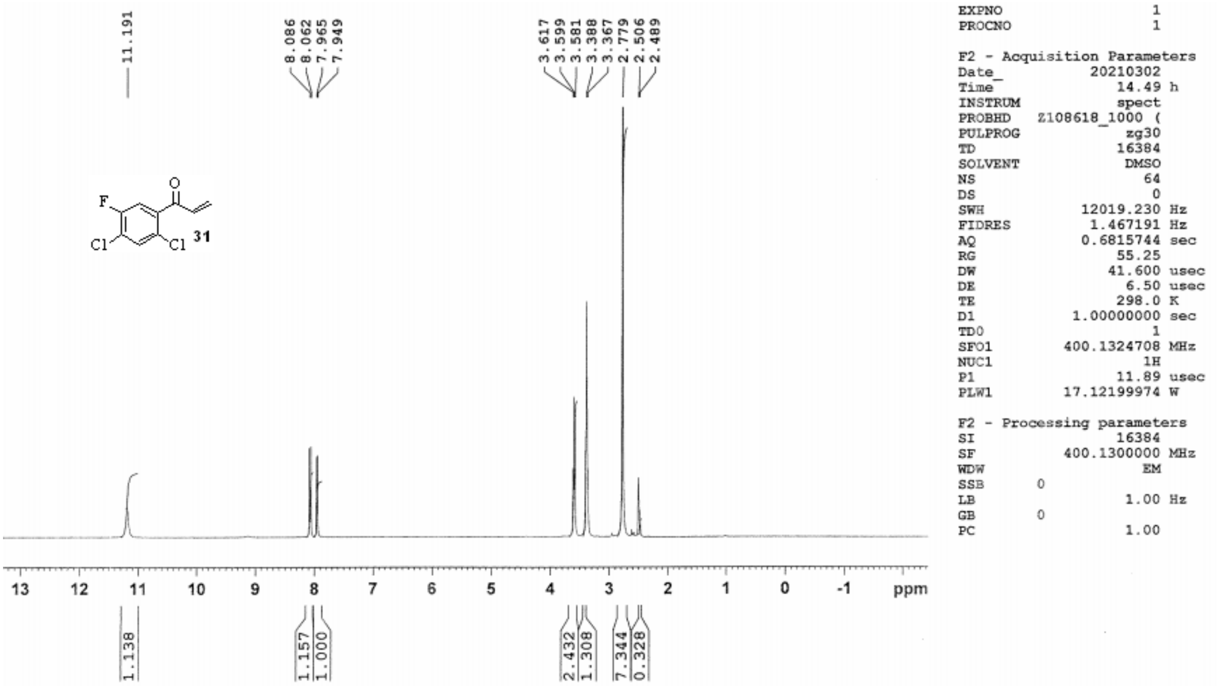

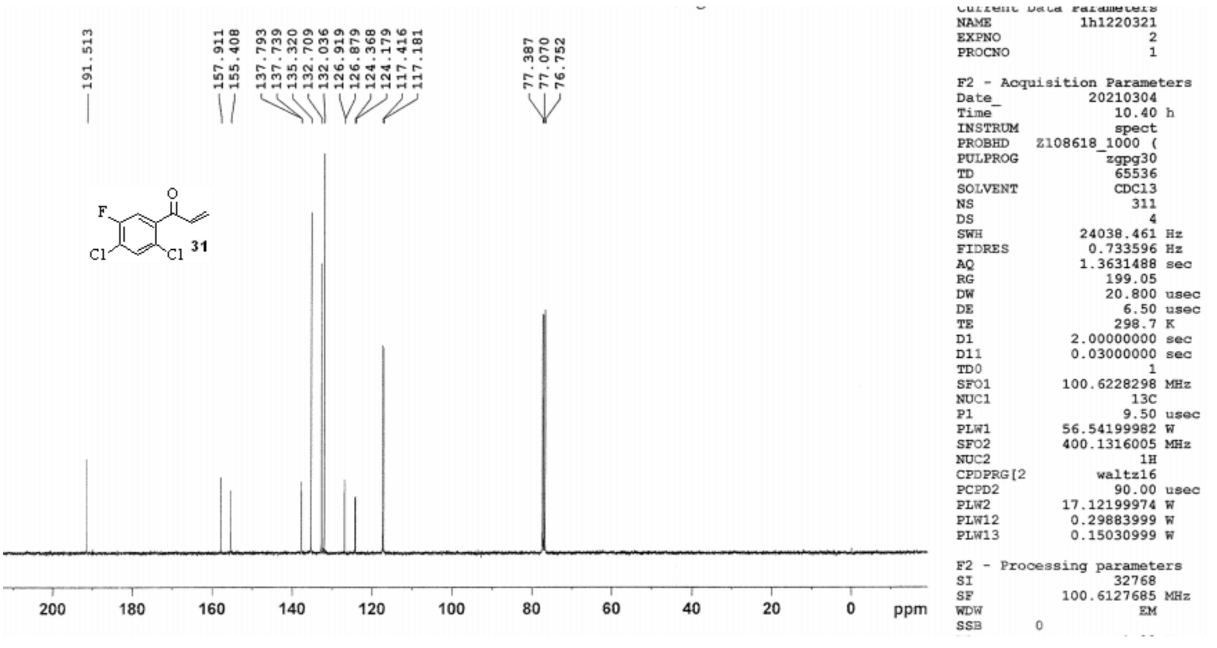

3-Dimethylamino-1-thiophen-2-yl-propan-1-one hydrochloride **(19)**

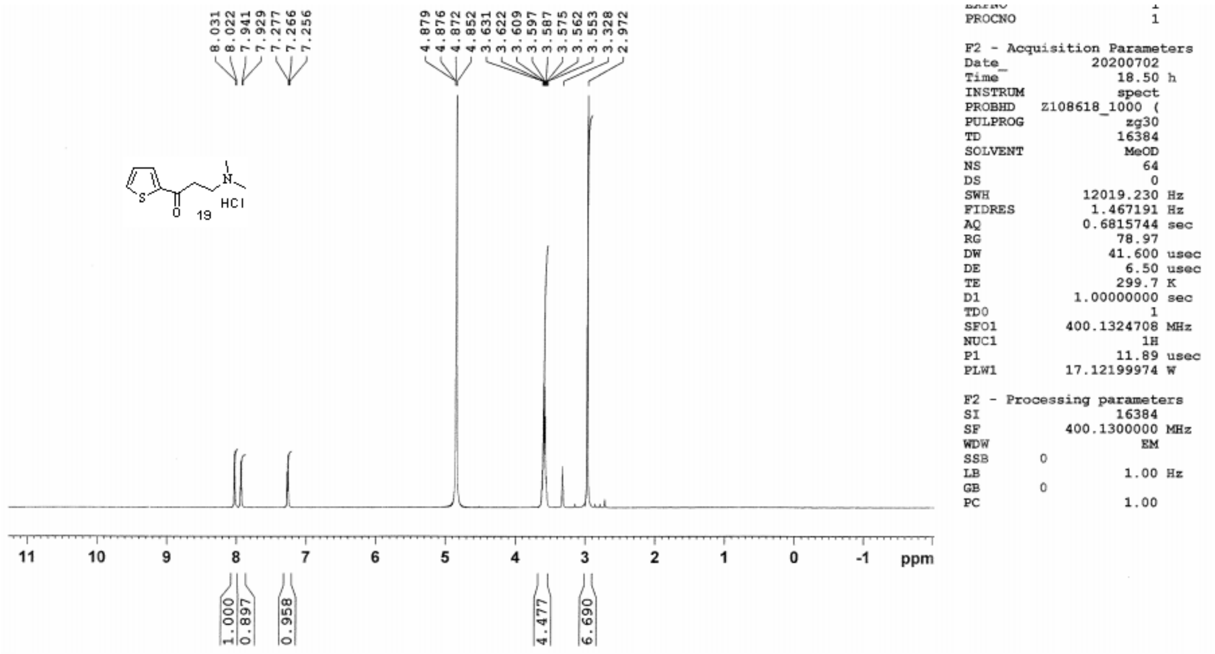

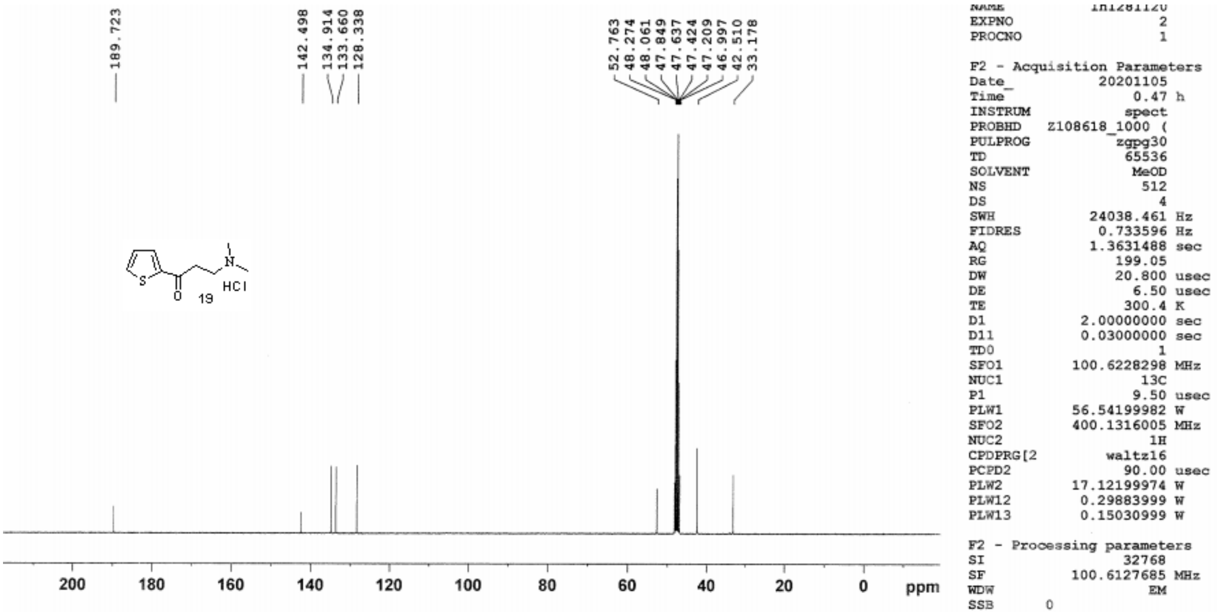

1-Thiophen-2-yl-propenone **(32)**

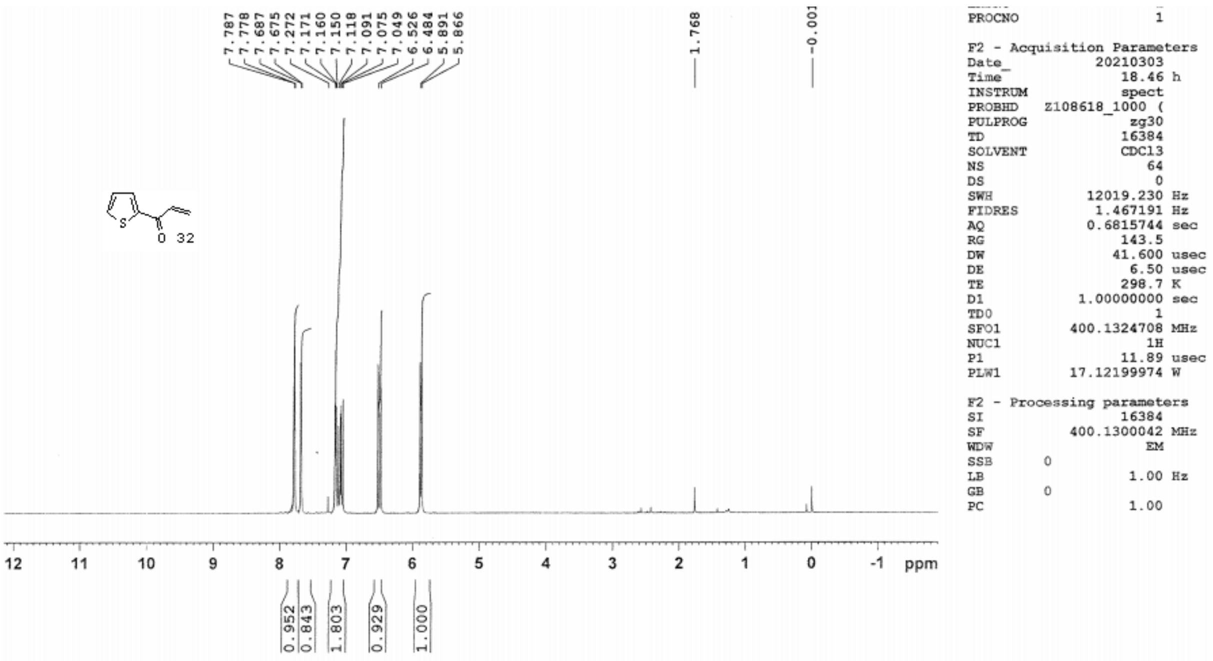

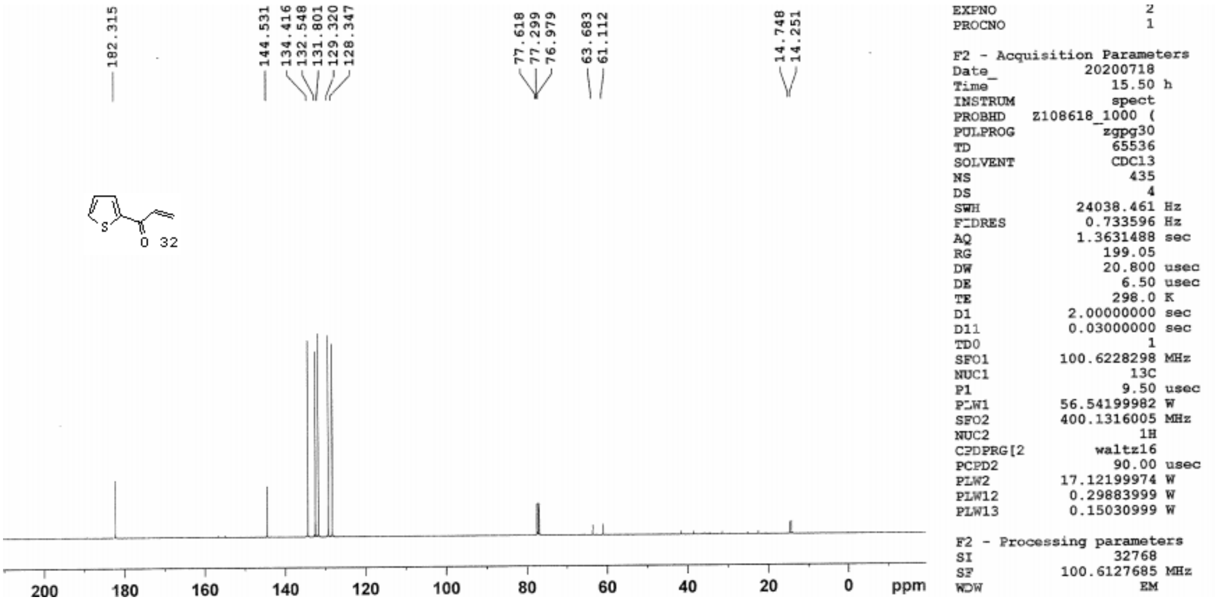

3-Dimethylamino-1-furan-2-yl-propan-1-one hydrochloride **(20)**

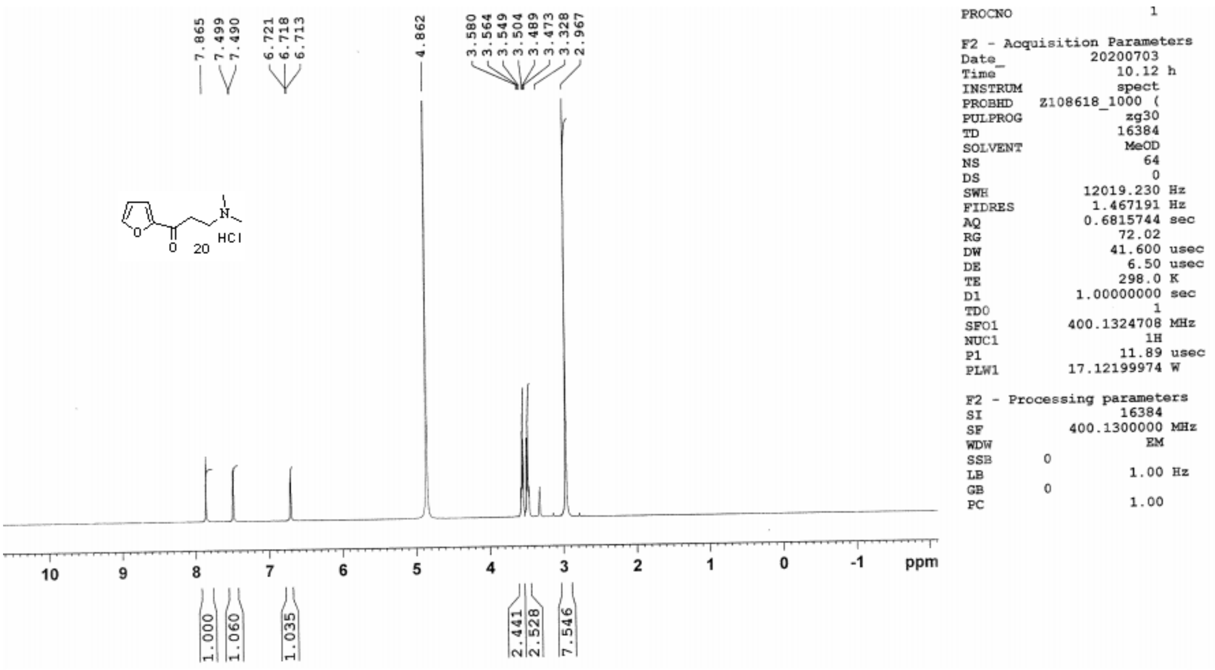

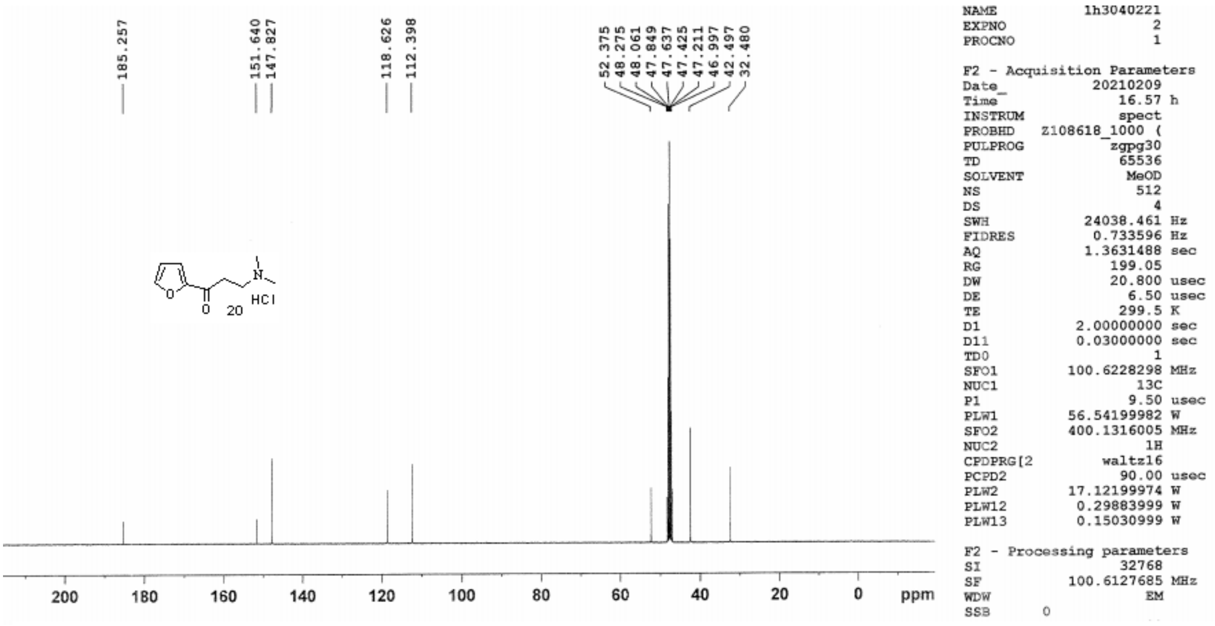

1-Furan-2-yl-propenone **(33)**

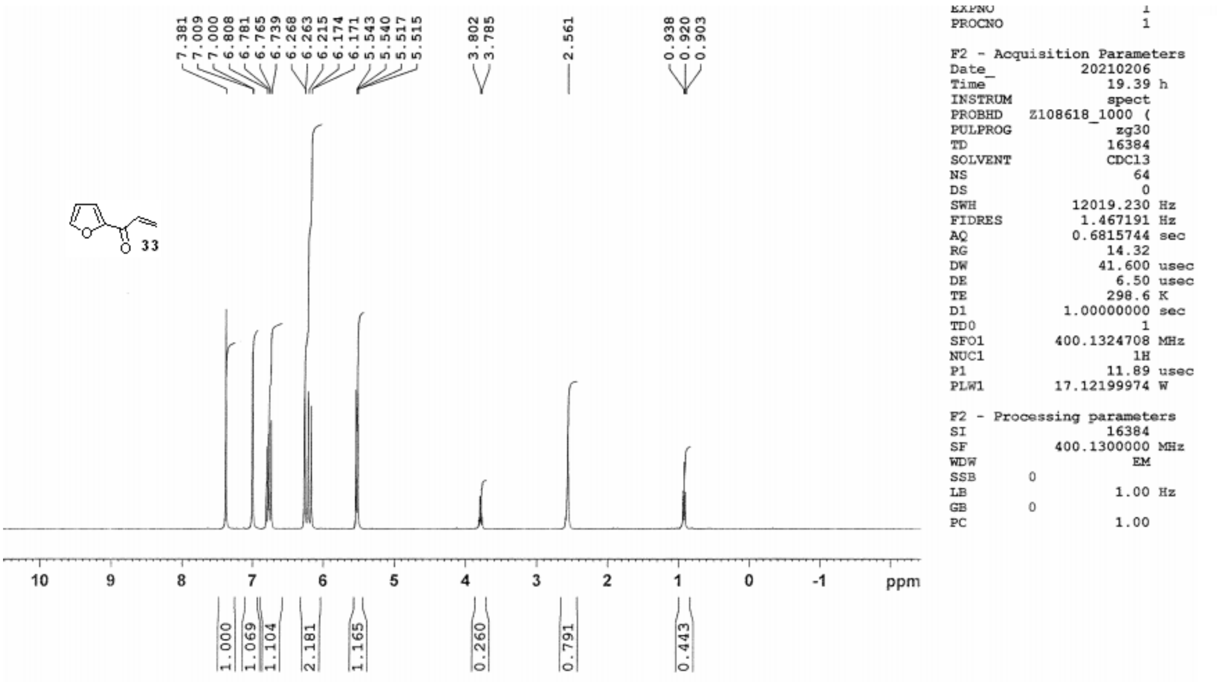

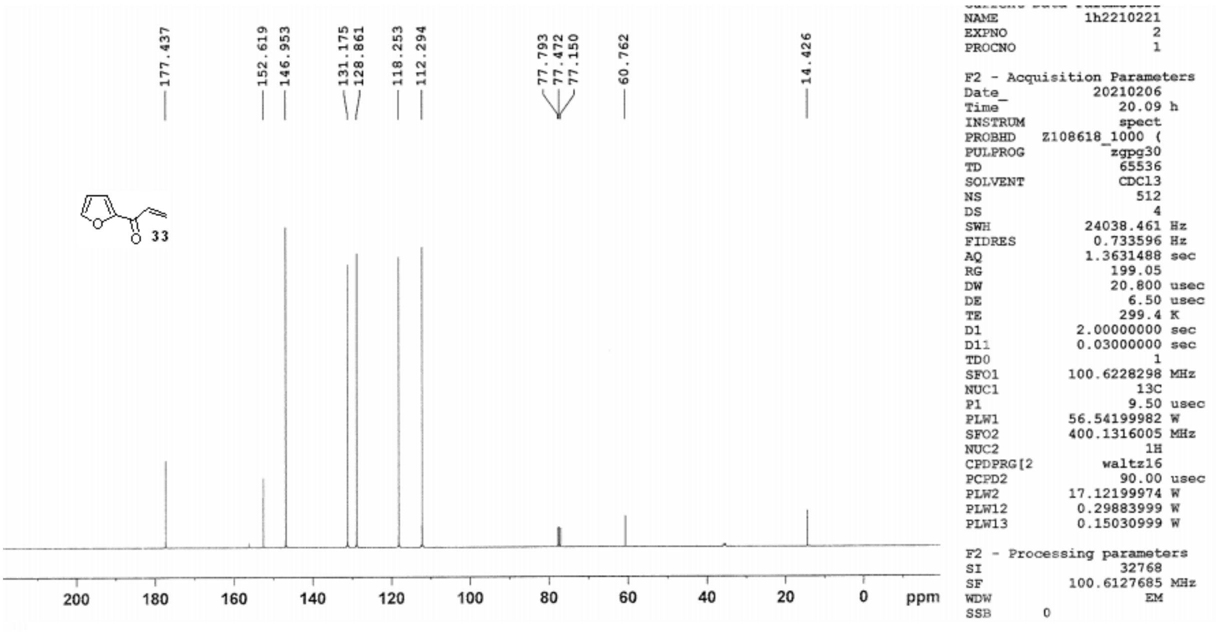

3-Dimethylamino-1-(4-phenoxyphenyl)-propan-1-one hydrochloride **(21)**

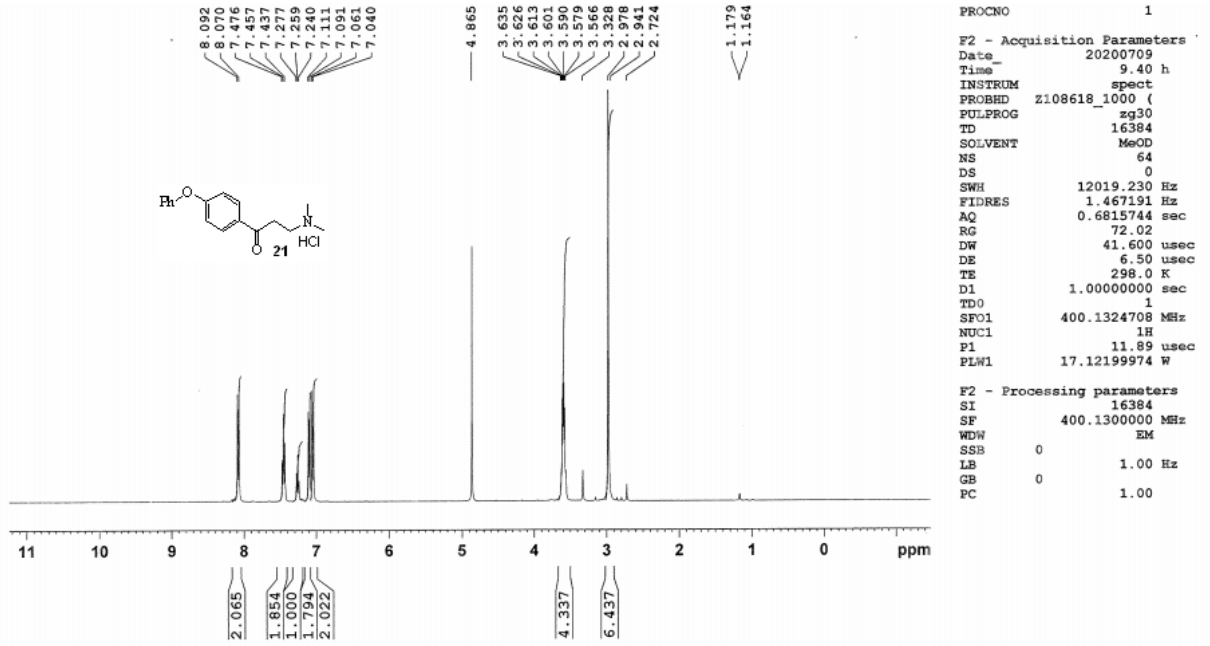

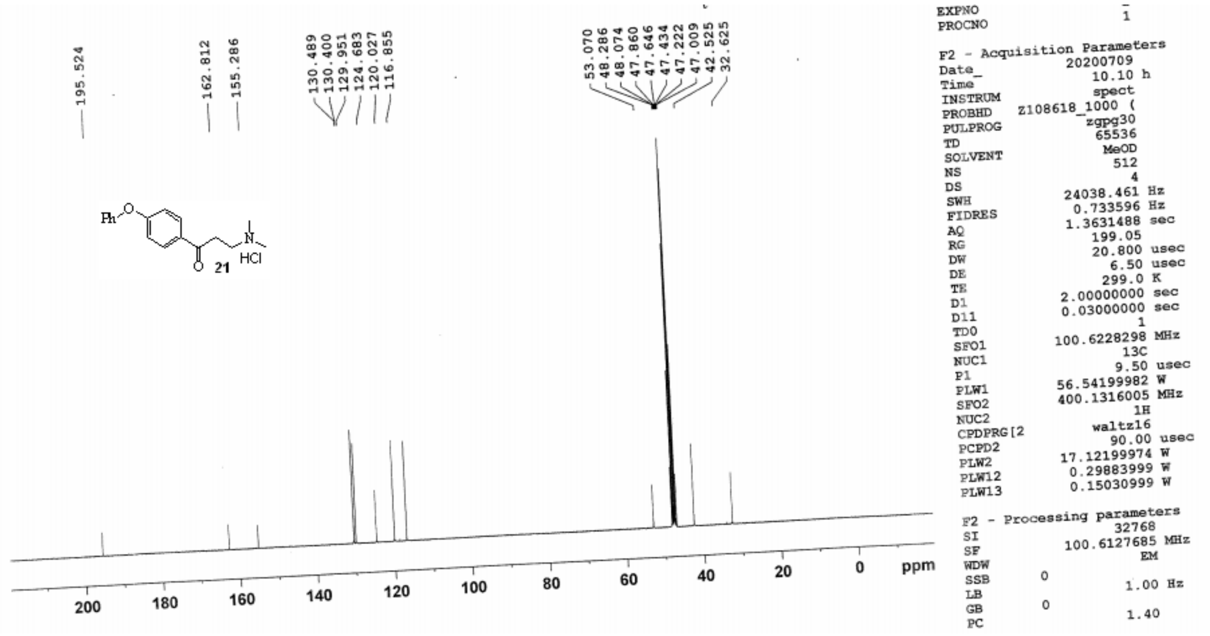

1-(4-Phenoxy-phenyl)-propenone **(34)**

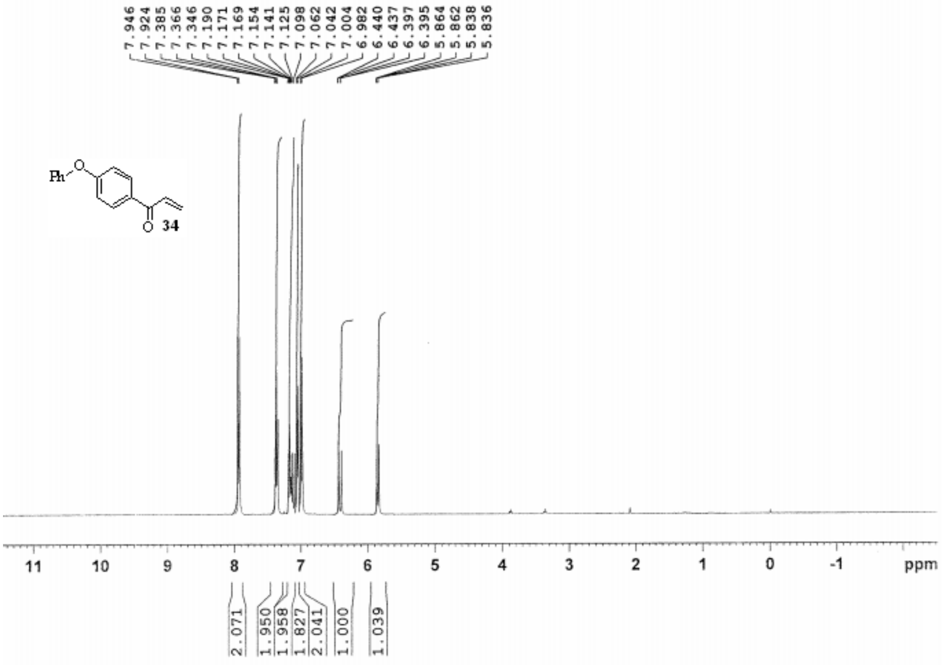

PROCNO 1

F2 - Acquisition Parameters
Date_ 20210227
Time 14.00 h
INSTRUM spect
PROBHD Z108618_1000 (
PULPROG zg30
TD 16384
SOLVENT CDCl3
NS 64
DS 0
SWH 12019.230 Hz
FIDRES 1.467191 Hz
AQ 0.6815744 sec
RG 24.42
DW 41.600 usec
DE 6.50 usec
TE 298.6 K
D1 1.00000000 sec
TD0 1
SFO1 400.1324708 MHz
NUC1 1H
P1 11.89 usec
PLW1 17.12199974 W

F2 - Processing parameters
SI 16384
SF 400.1300101 MHz
WDW EM
SSB 0
LB 1.00 Hz
GB 0
PC 1.00

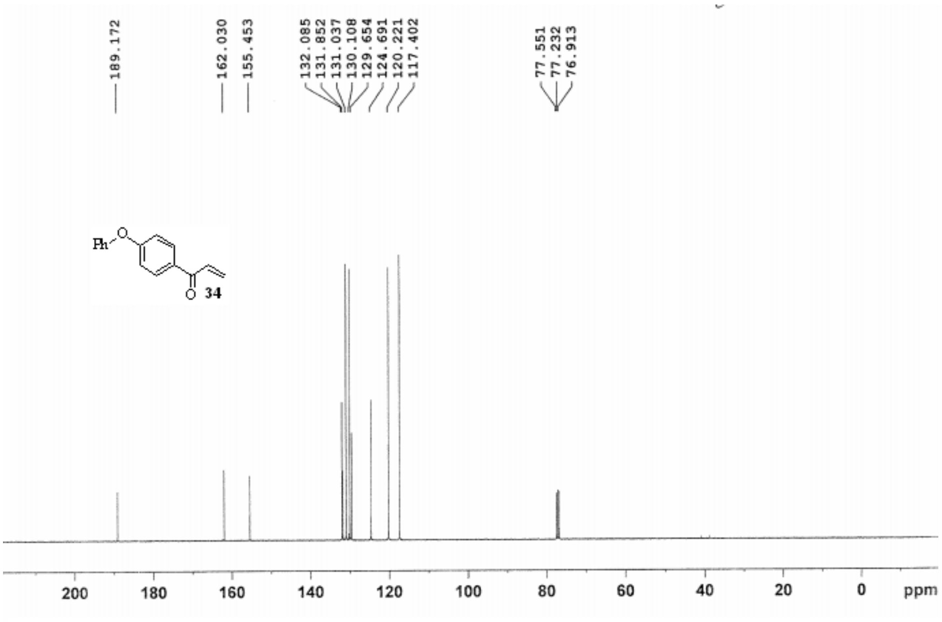

NAME 1h10230221
EXPNO 2
PROCNO 1

F2 - Acquisition Parameters
Date_ 20210227
Time 14.08 h
INSTRUM spect
PROBHD Z108618_1000 (
PULPROG zgpg30
TD 65536
SOLVENT CDCl3
NS 128
DS 4
SWH 24038.461 Hz
FIDRES 0.733596 Hz
AQ 1.3631488 sec
RG 199.05
DW 20.800 usec
DE 6.50 usec
TE 298.5 K
D1 2.00000000 sec
D11 0.03000000 sec
TD0 1
SFO1 100.6228298 MHz
NUC1 13C
P1 9.50 usec
PLW1 56.54199982 W
SFO2 400.1316005 MHz
NUC2 1H
CPDPRG[2 waltz16
PCPD2 90.00 usec
PLW2 17.12199974 W
PLW12 0.29883999 W
PLW13 0.15030999 W

F2 - Processing parameters
SI 32768
SF 100.6127685 MHz
WDW EM
SSB 0

3-Dimethylamino-1-(2-hydroxy-phenyl)-propan-1-one hydrochloride **(22)**

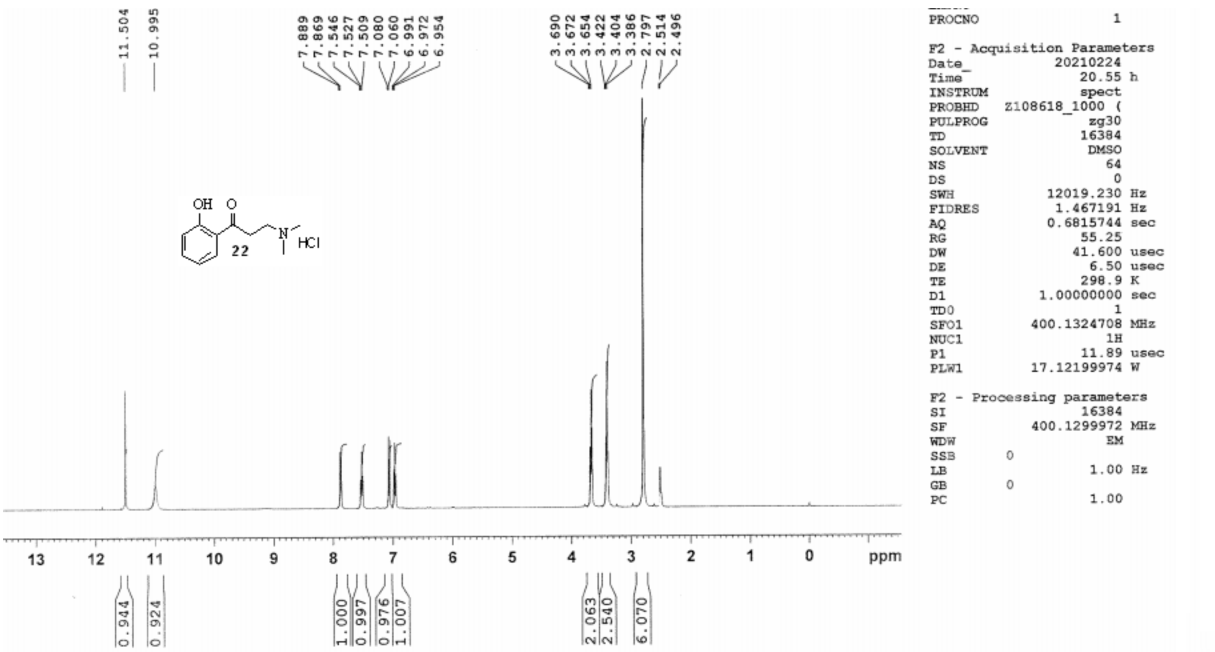

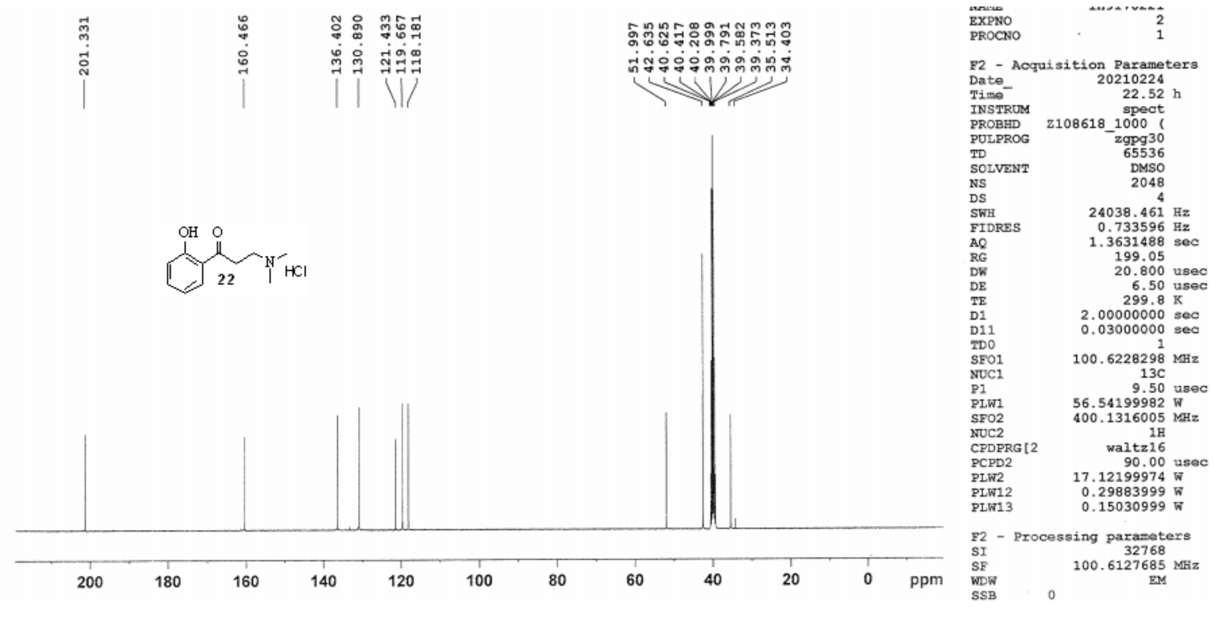

1-(2-hydroxyphenyl)prop-2-en-1-one **(35).**

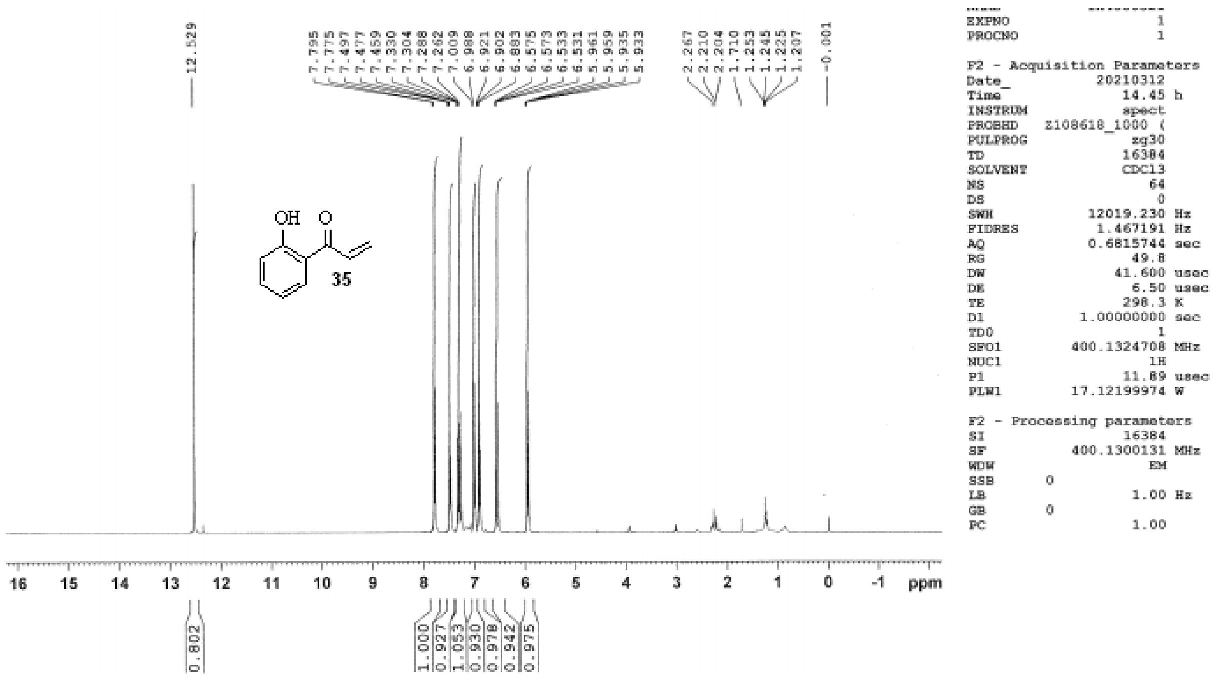

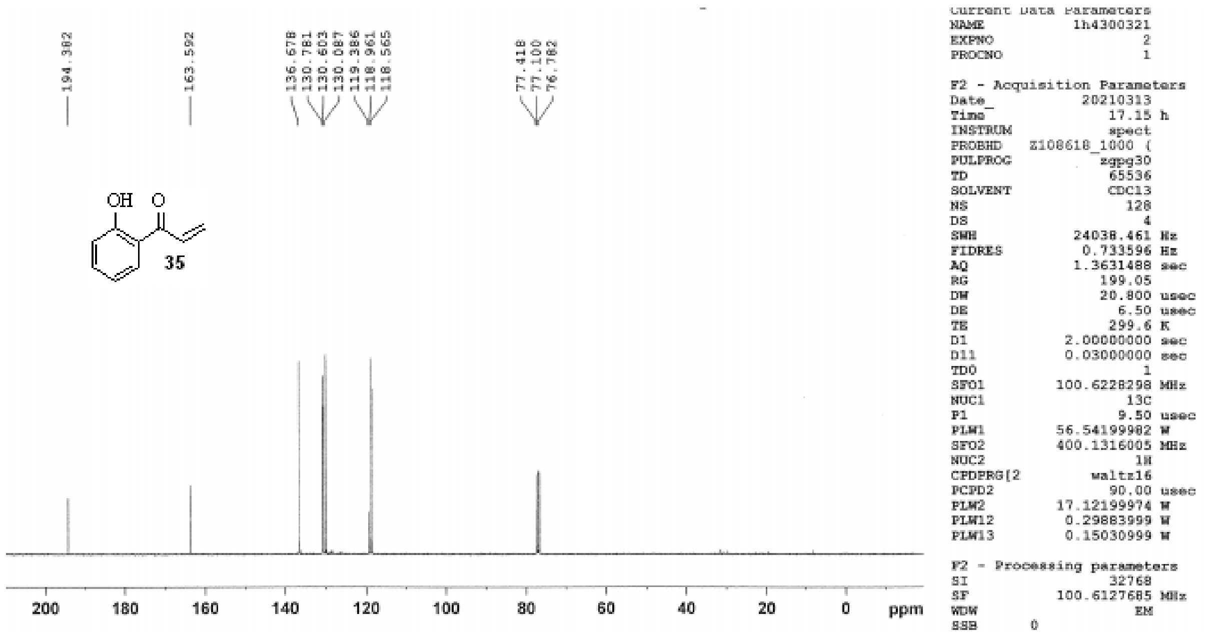

3-Dimethylamino-1-naphthalen-2-yl-propan-1-one hydrochloride **(23)**

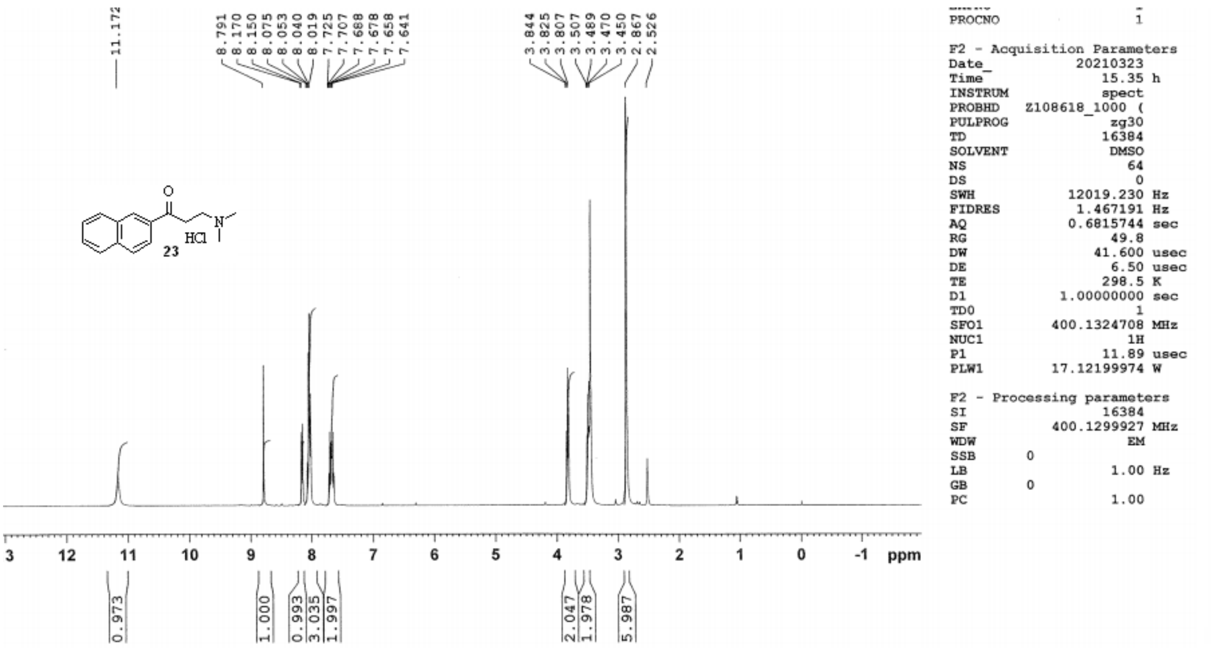

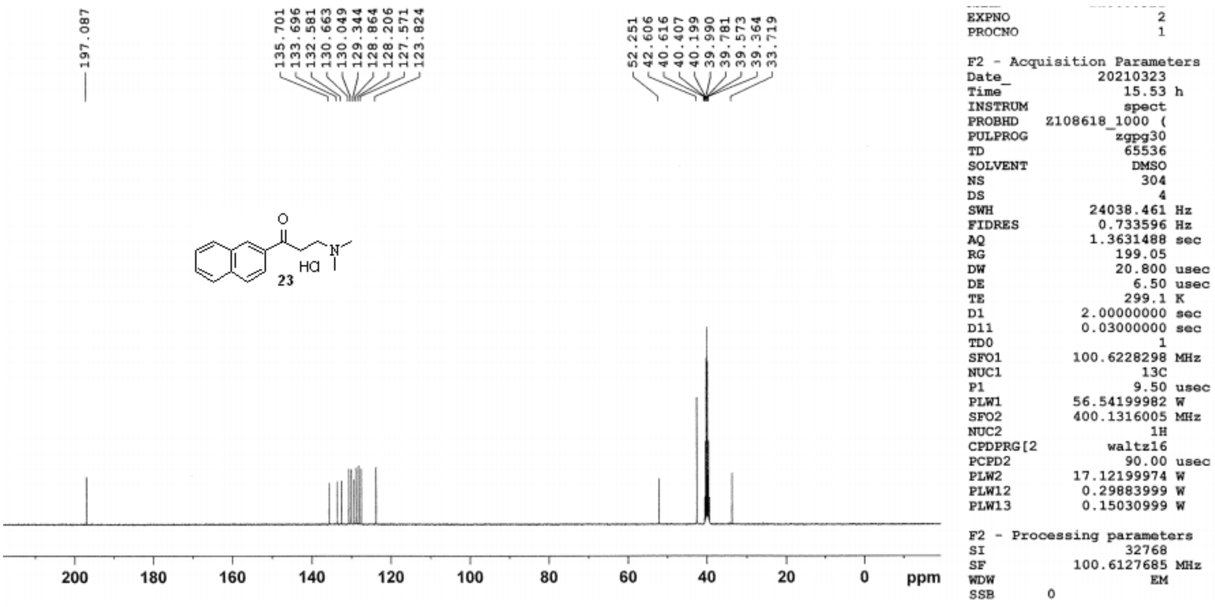

1-Naphthalen-2-yl-propenone **(36)**

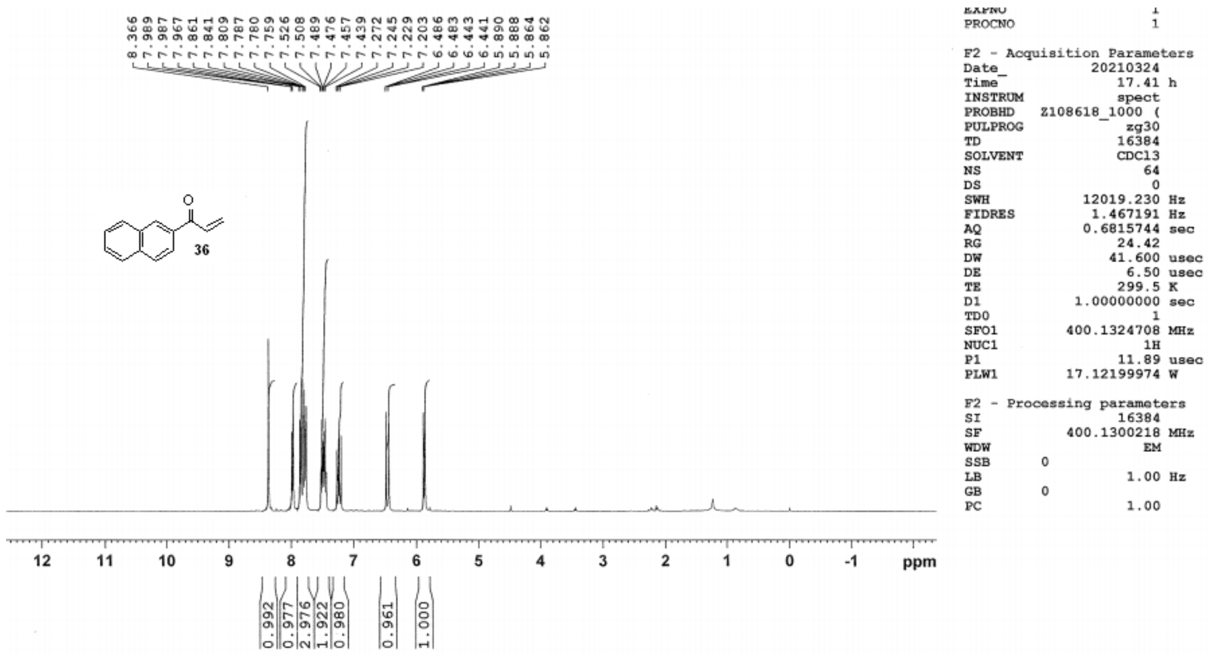

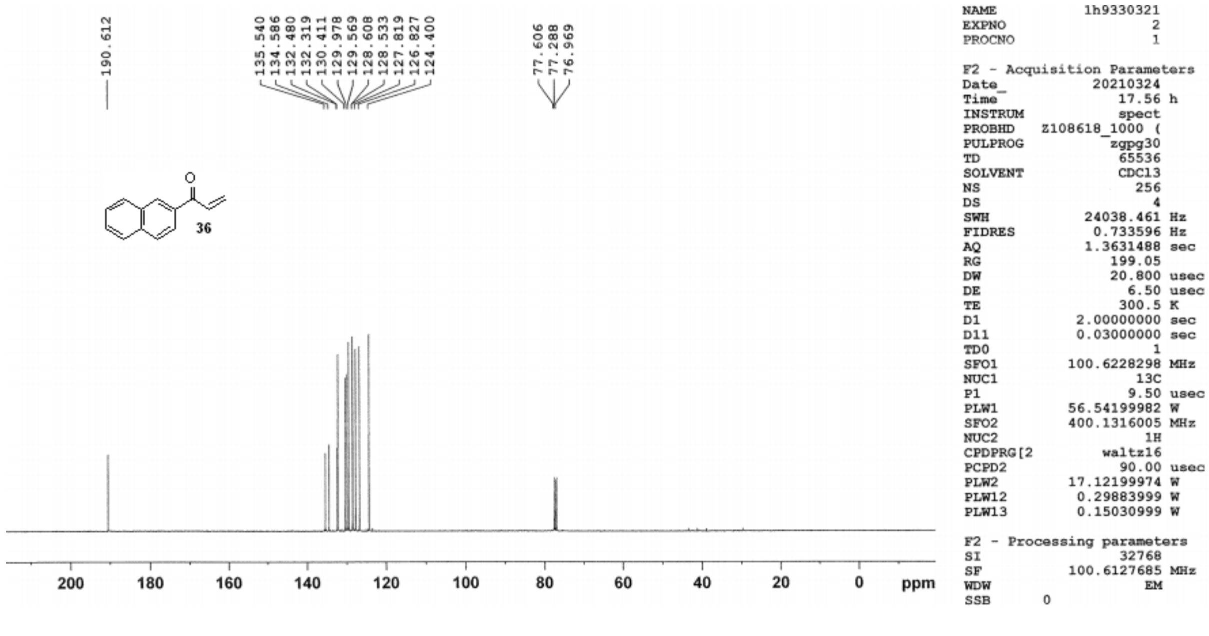

3-Dimethylamino-1-naphthalen-1-yl-propan-1-one hydrochloride **(24)**

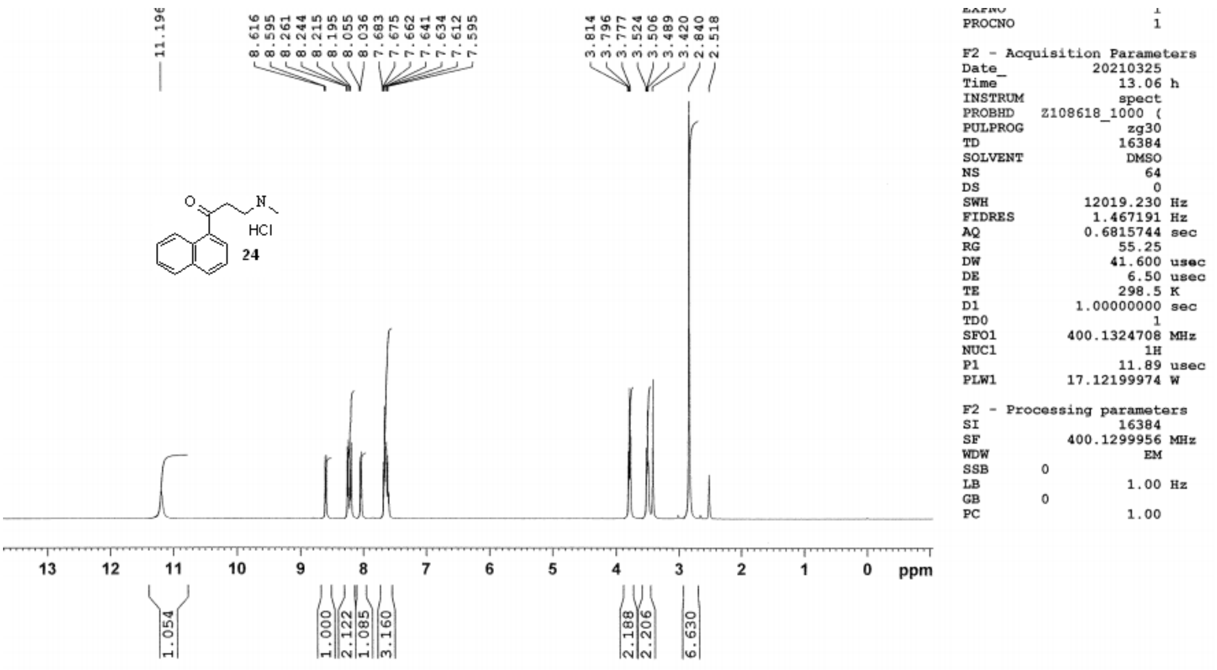

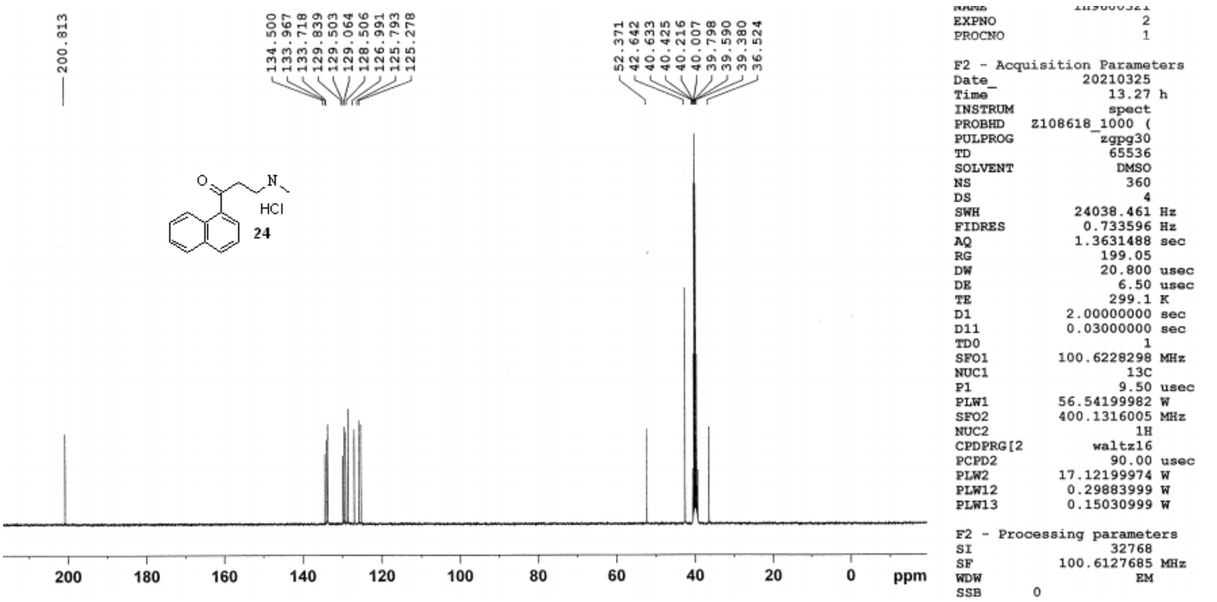

1-Naphthalen-1-yl-propenone **(37)**

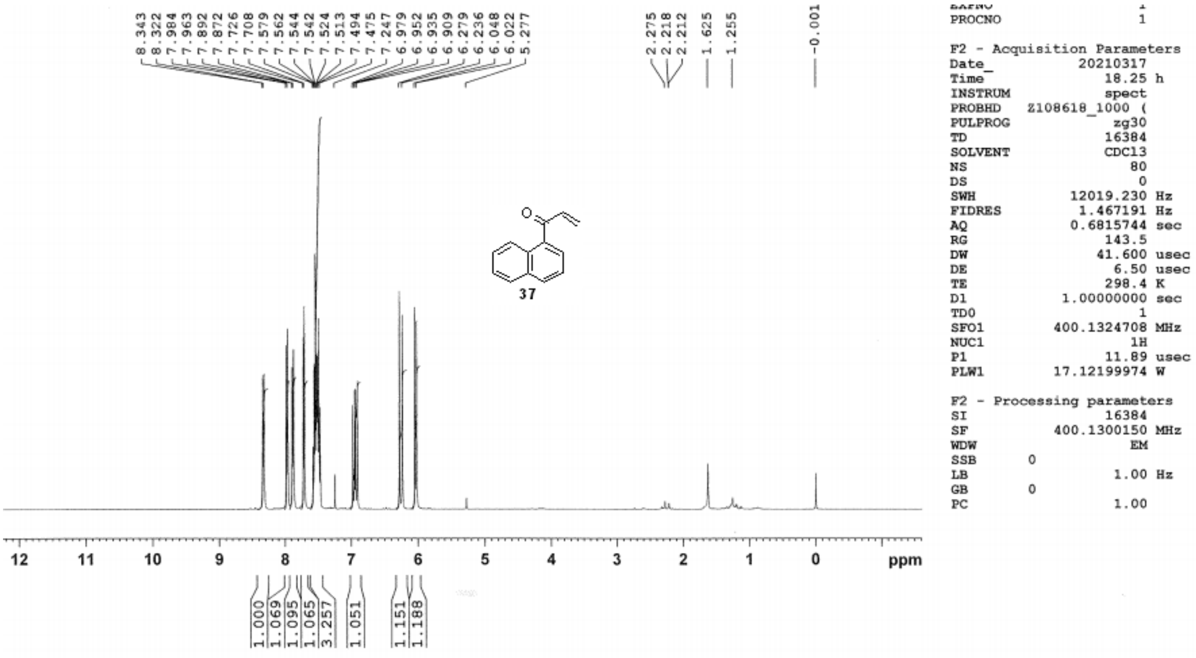

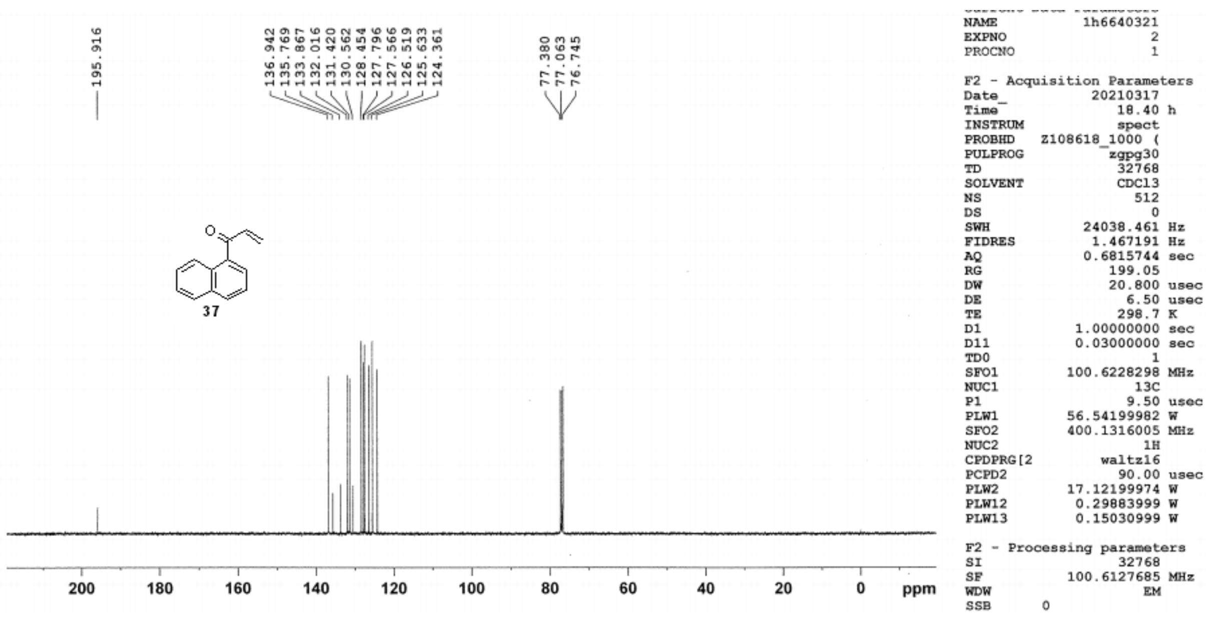

3-(dimethylamino)-1-(p-tolyl)propan-1-one hydrochloride **(25)**

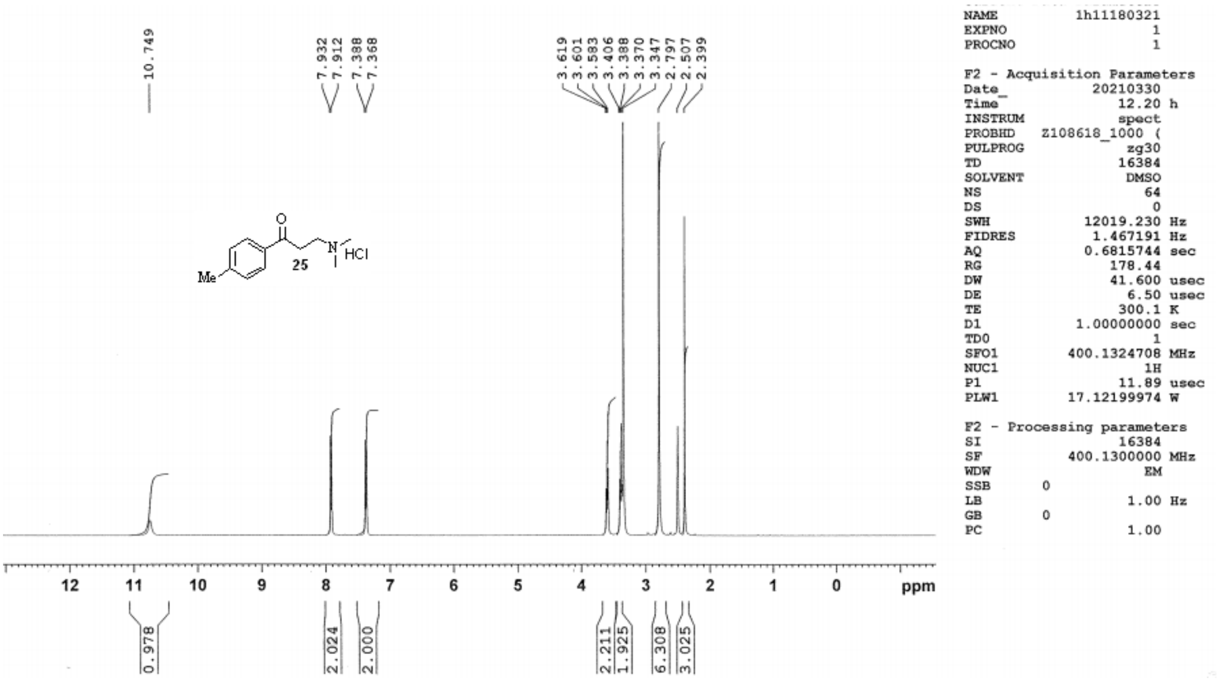

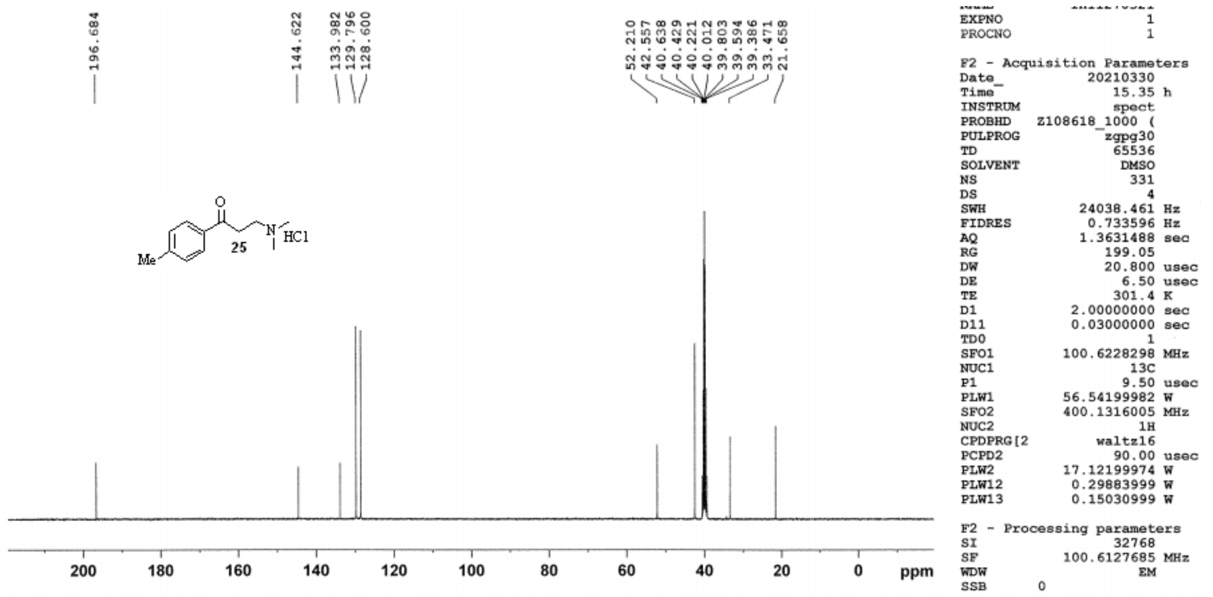

1-(p-tolyl)prop-2-en-1-one **(38)**

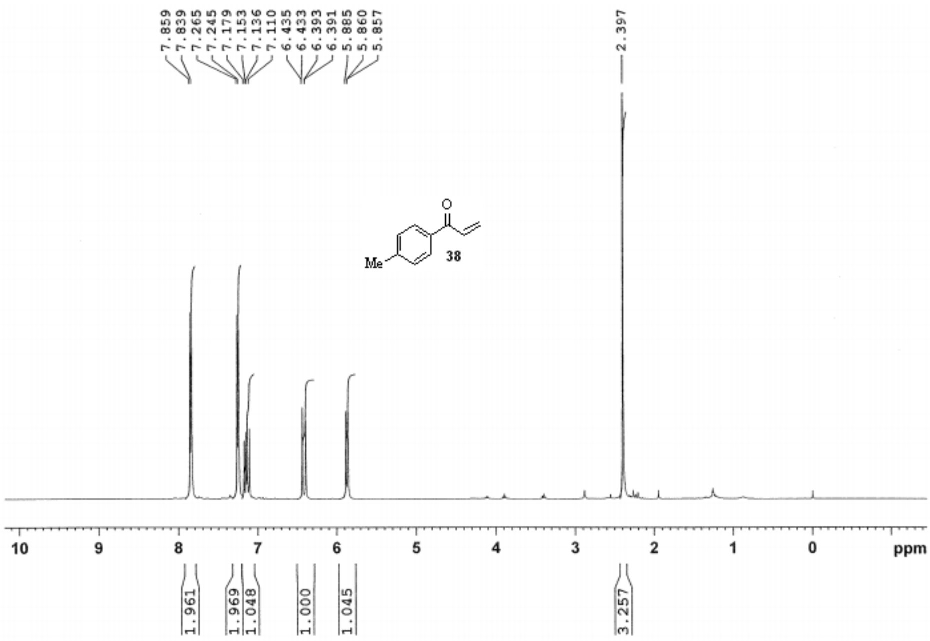


```
PROCNO              1

F2 - Acquisition Parameters
Date_          20210330
Time              18.15 h
INSTRUM           spect
PROBHD   Z108618_1000 (
PULPROG            zg30
TD                16384
SOLVENT           CDCl3
NS                   64
DS                    0
SWH           12019.230 Hz
FIDRES         1.467191 Hz
AQ            0.6815744 sec
RG                39.68
DW               41.600 usec
DE                 6.50 usec
TE                301.2 K
D1           1.00000000 sec
TD0                   1
SFO1        400.1324708 MHz
NUC1                 1H
P1                11.89 usec
PLW1        17.12199974 W

F2 - Processing parameters
SI                16384
SF          400.1300067 MHz
WDW                  EM
SSB      0
LB                 1.00 Hz
GB       0
PC                 1.00
```

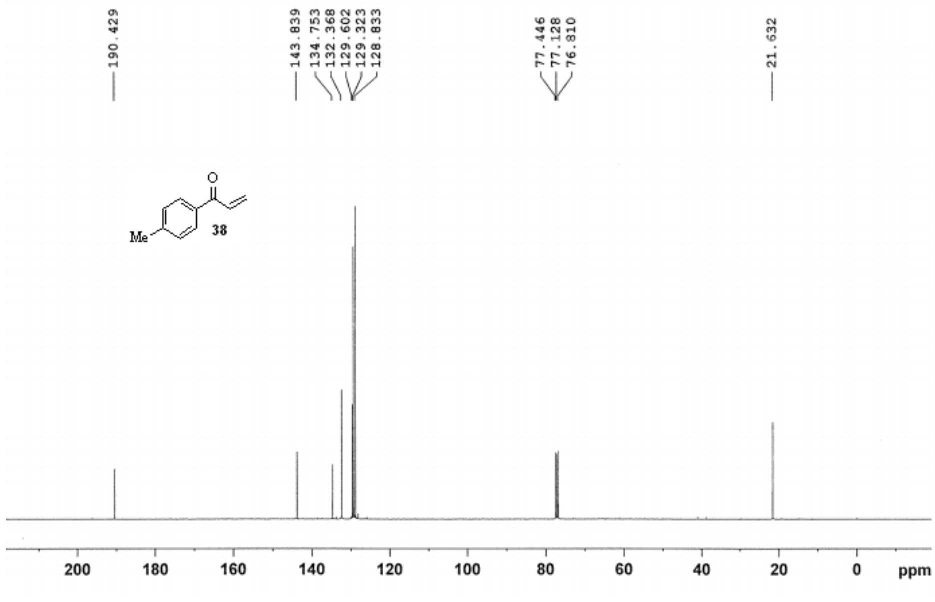


```
NAME          1h11400321
EXPNO                 2
PROCNO                1

F2 - Acquisition Parameters
Date_          20210330
Time              18.45 h
INSTRUM           spect
PROBHD   Z108618_1000 (
PULPROG          zgpg30
TD                65536
SOLVENT           CDCl3
NS                  512
DS                    4
SWH           24038.461 Hz
FIDRES         0.733596 Hz
AQ            1.3631488 sec
RG               199.05
DW               20.800 usec
DE                 6.50 usec
TE                300.6 K
D1           2.00000000 sec
D11          0.03000000 sec
TD0                   1
SFO1        100.6228298 MHz
NUC1                13C
P1                 9.50 usec
PLW1        56.54199982 W
SFO2        400.1316005 MHz
NUC2                 1H
CPDPRG[2        waltz16
PCPD2             90.00 usec
PLW2        17.12199974 W
PLW12        0.29883999 W
PLW13        0.15030999 W

F2 - Processing parameters
SI                32768
SF          100.6127685 MHz
WDW                  EM
SSB      0
```

1-(4-Chloro-phenyl)-3-dimethylamino-propan-1-one hydrochloride **(26).**

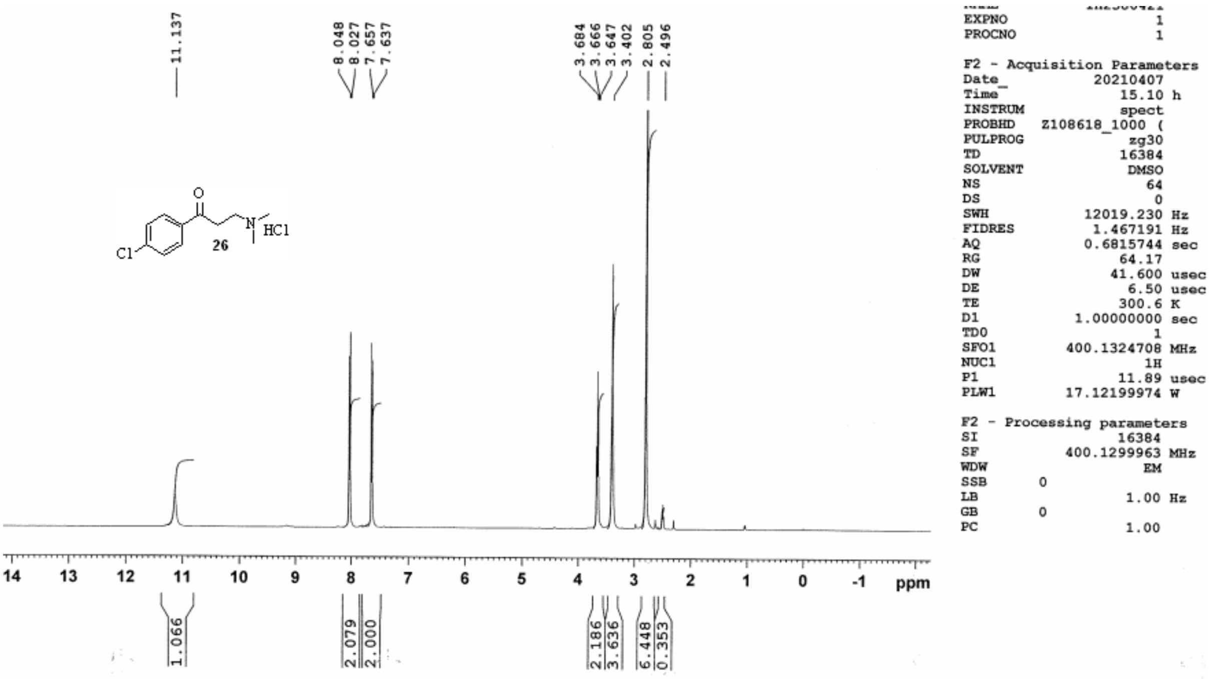

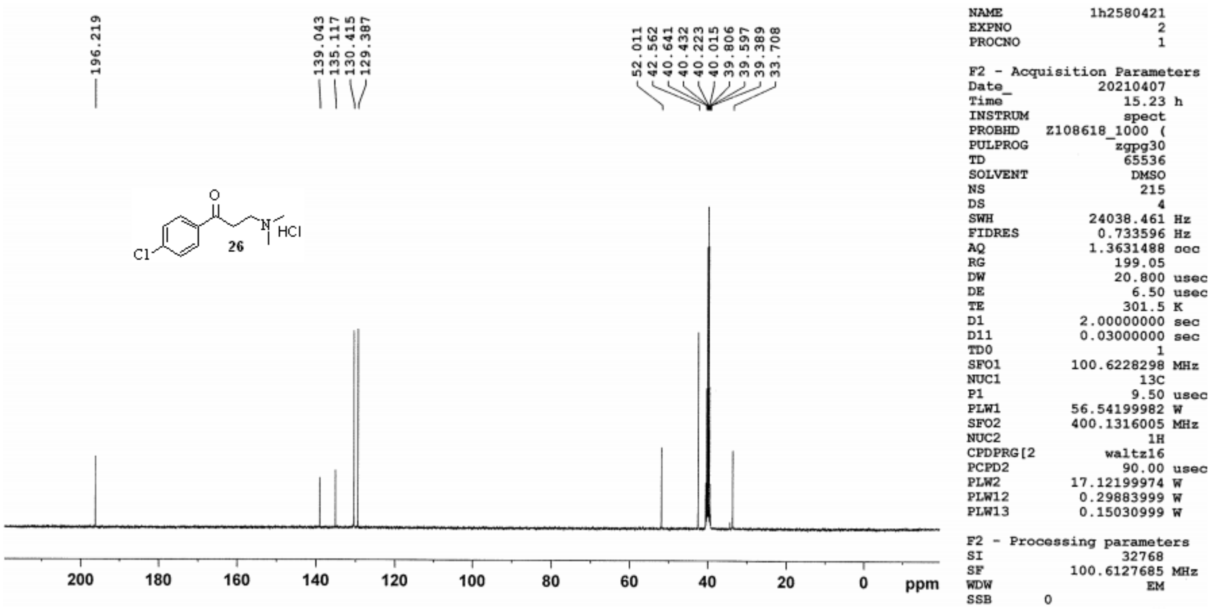

1-(4-Chloro-phenyl)-propenone **(39)**

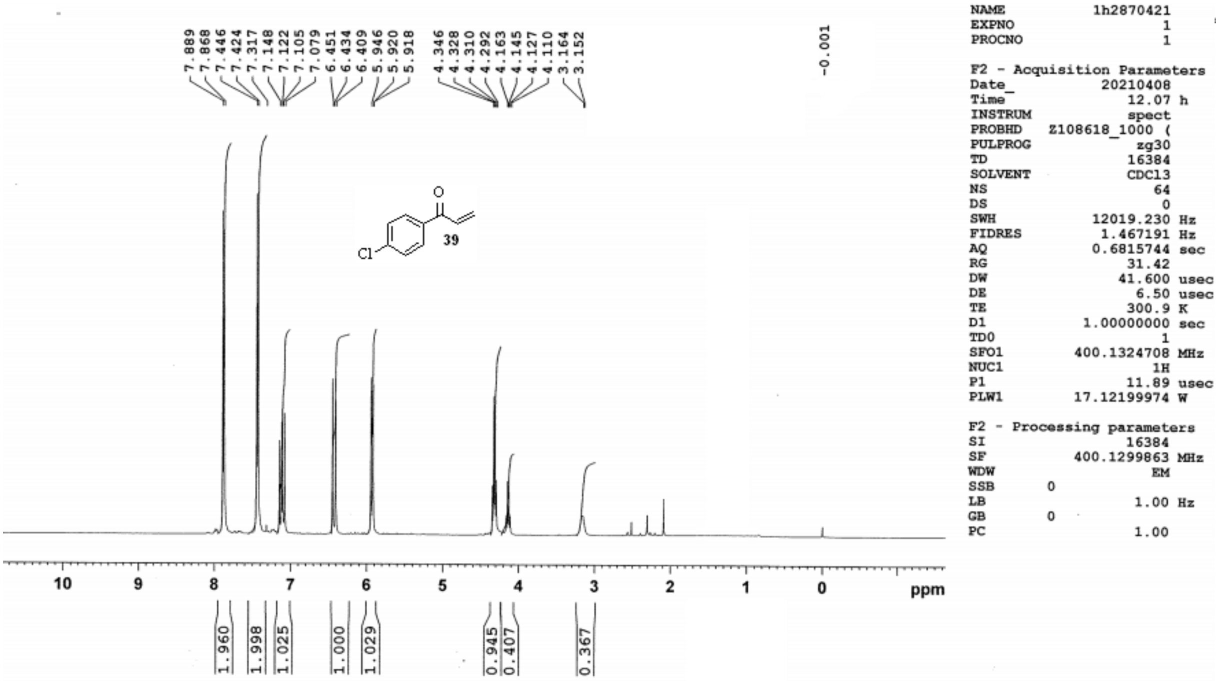

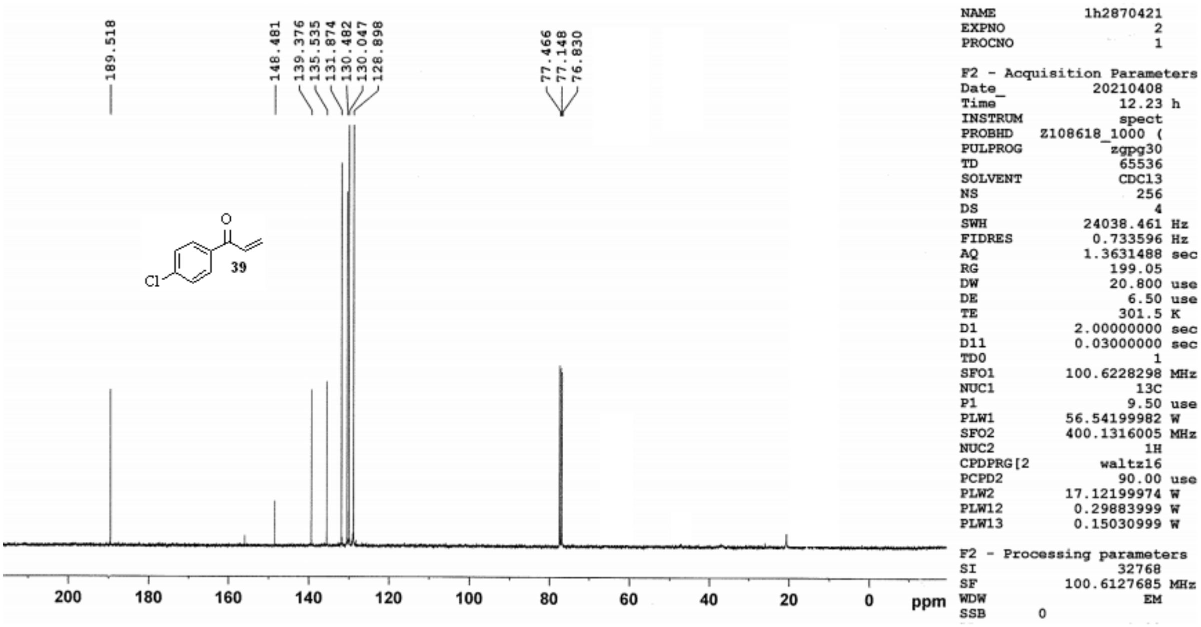

1-Methyl-2,3-dihydro-1H-quinolin-4-one **(40)**

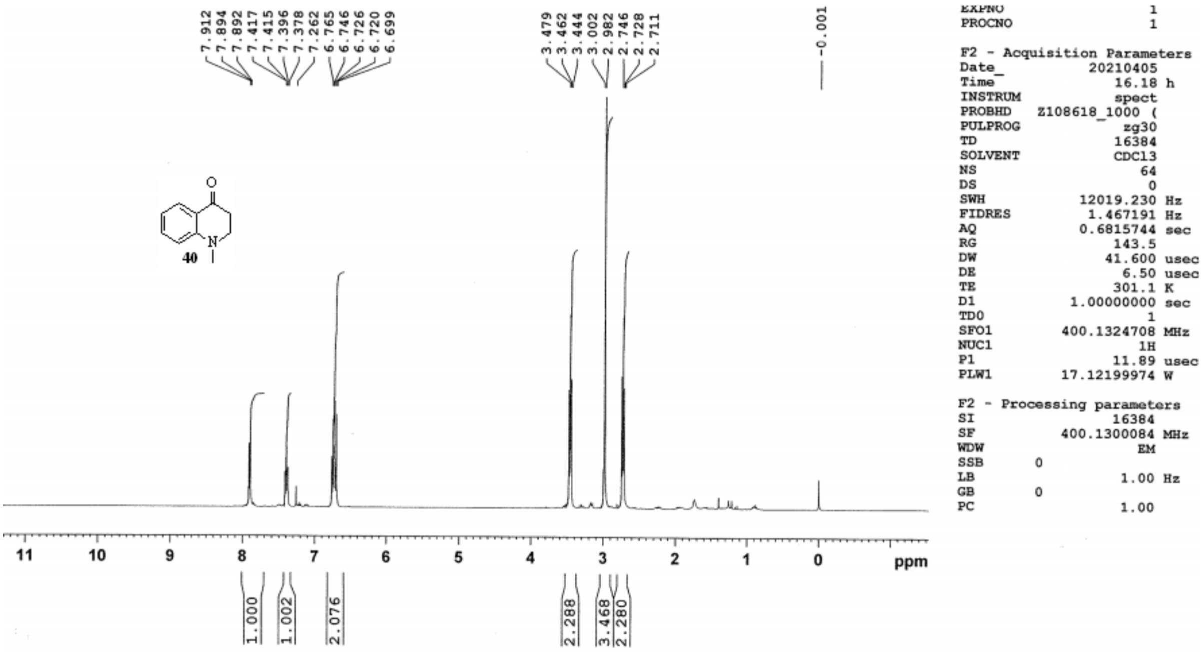

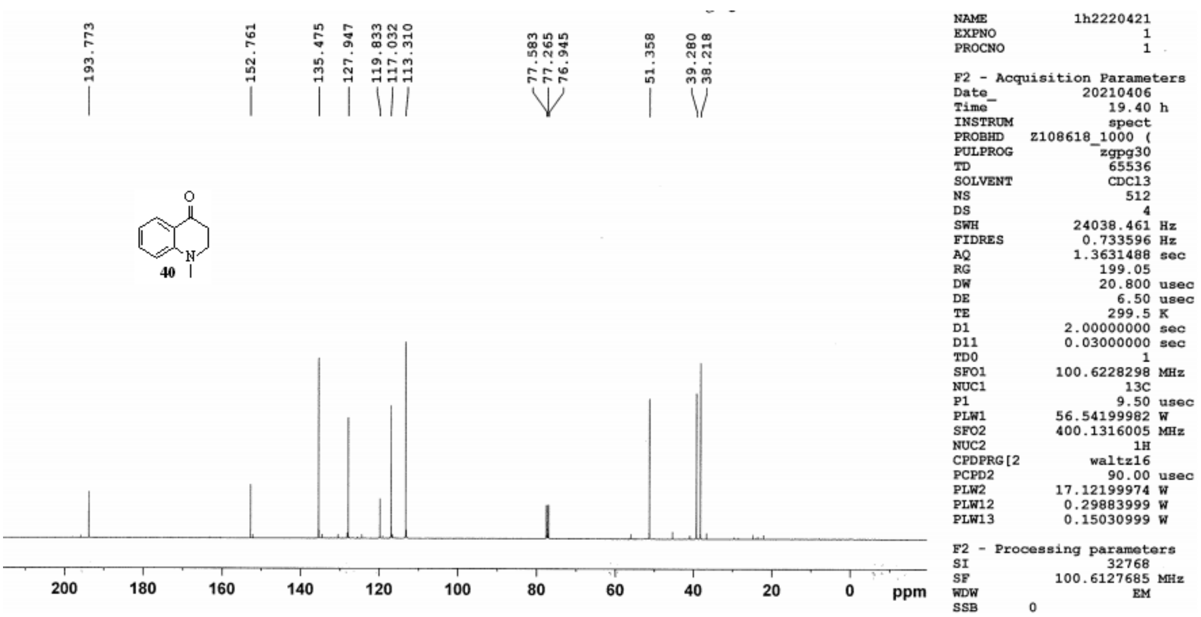

1-Benzyl-2,3-dihydro-1H-quinolin-4-one **(42)**

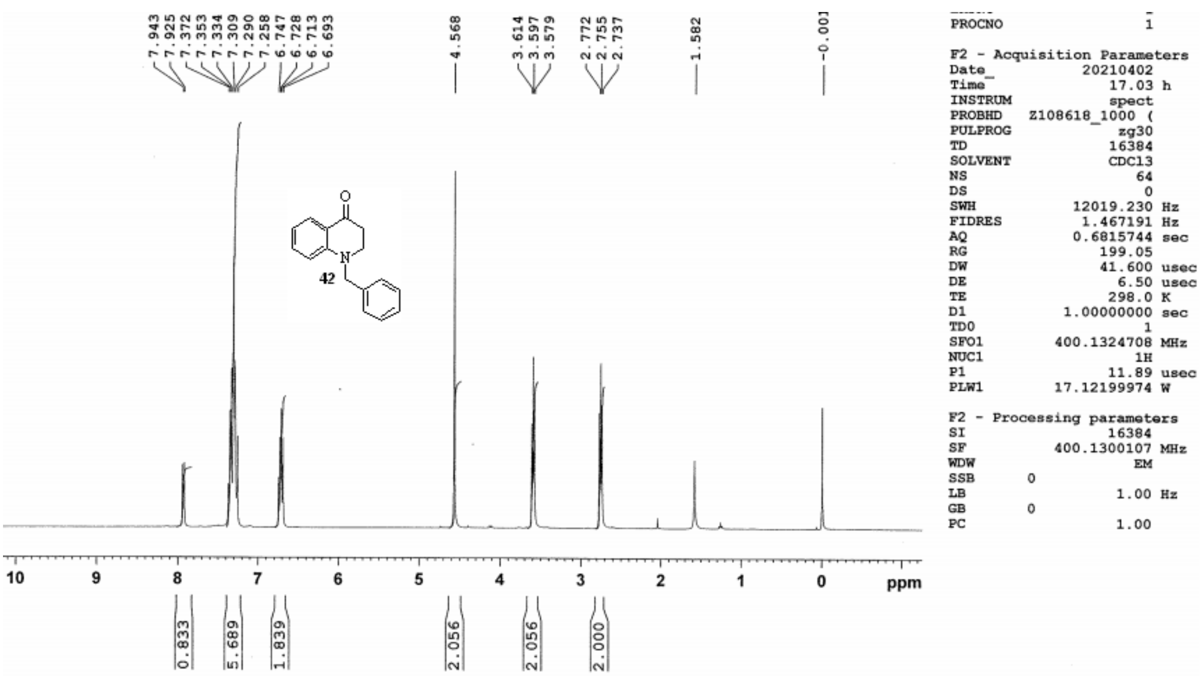

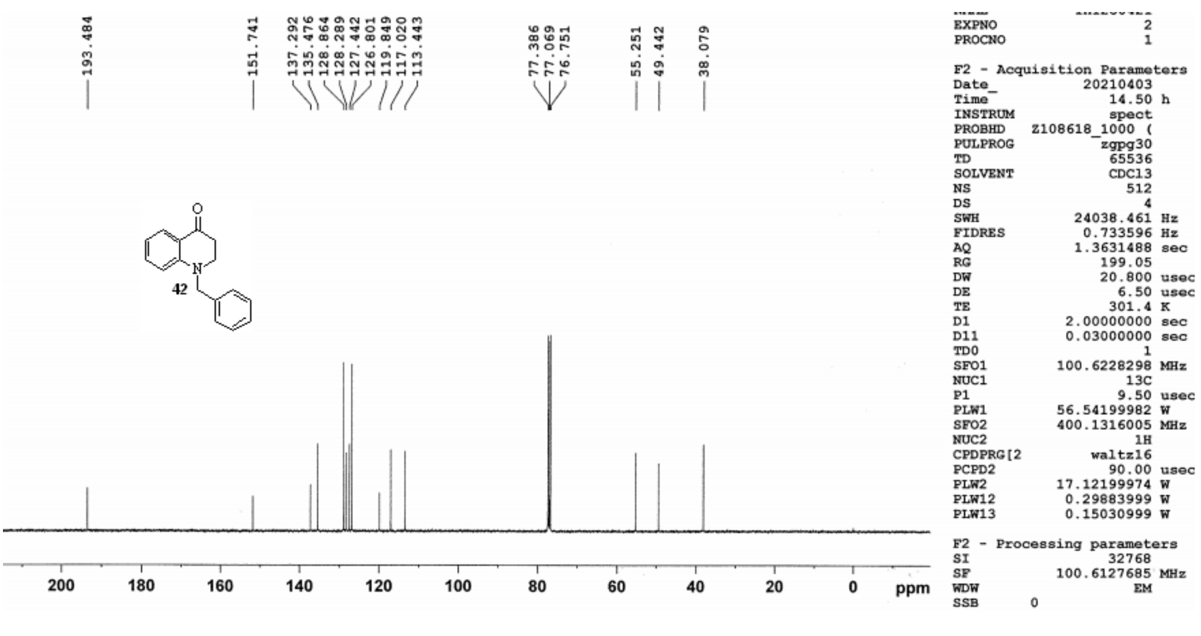

3-Dimethylamino-1-phenyl-propan-1-ol **(47)**

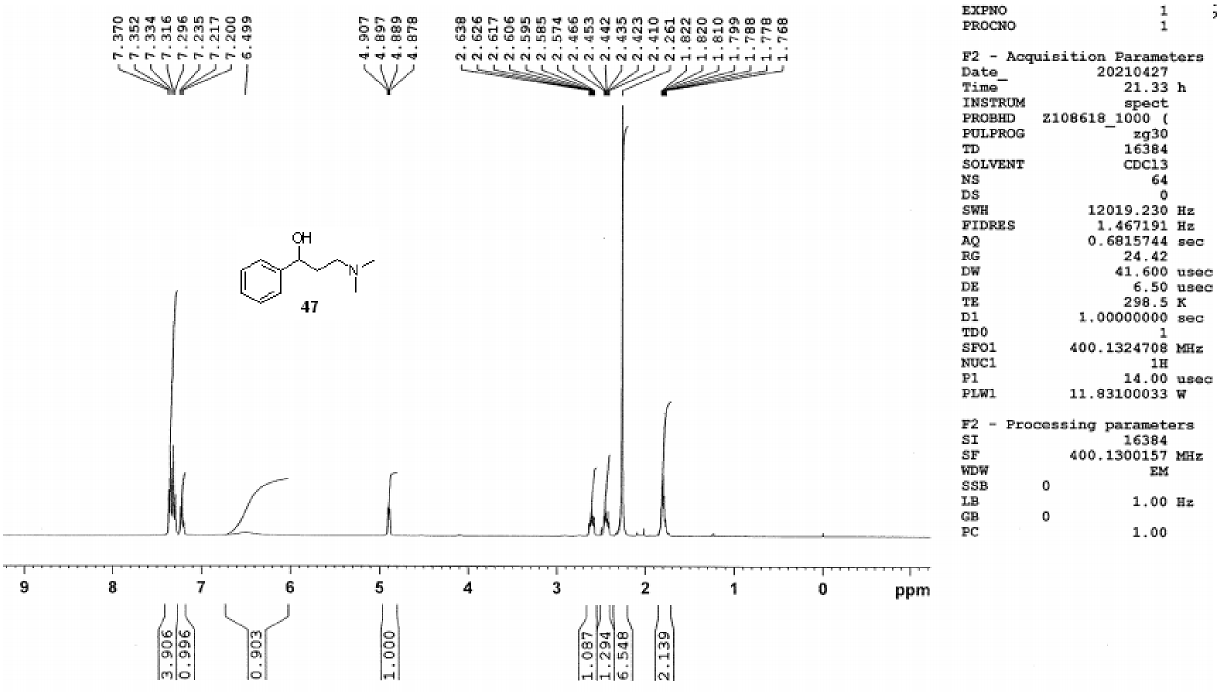

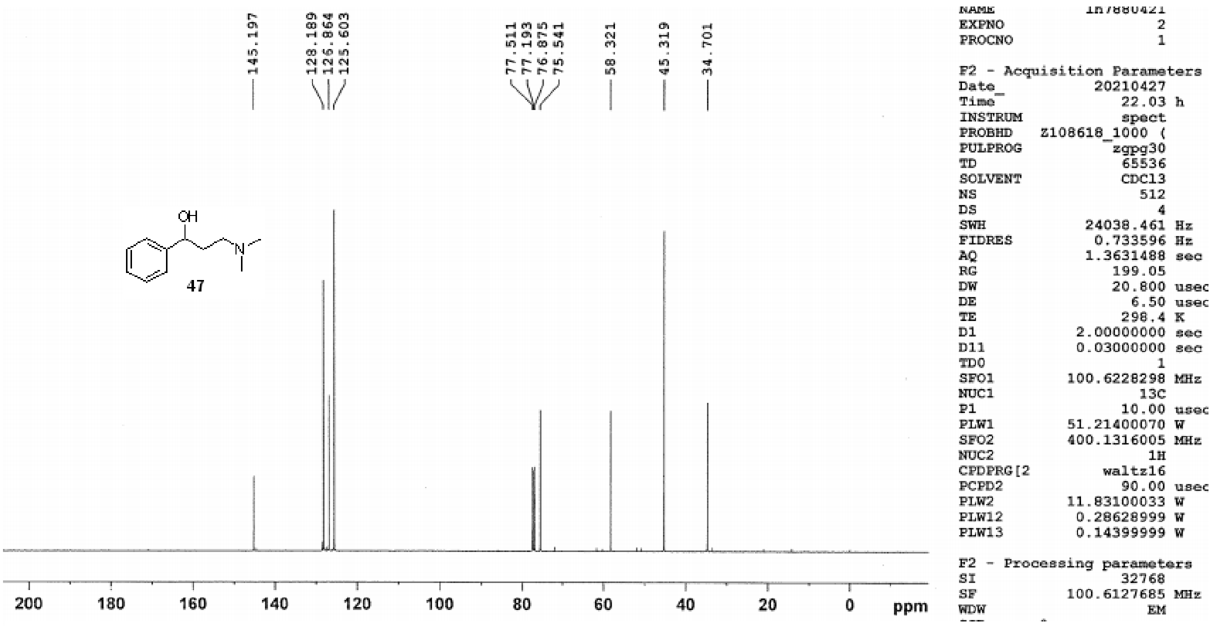

(3-Chloro-3-phenyl-propyl)-dimethyl-amine **(48)**

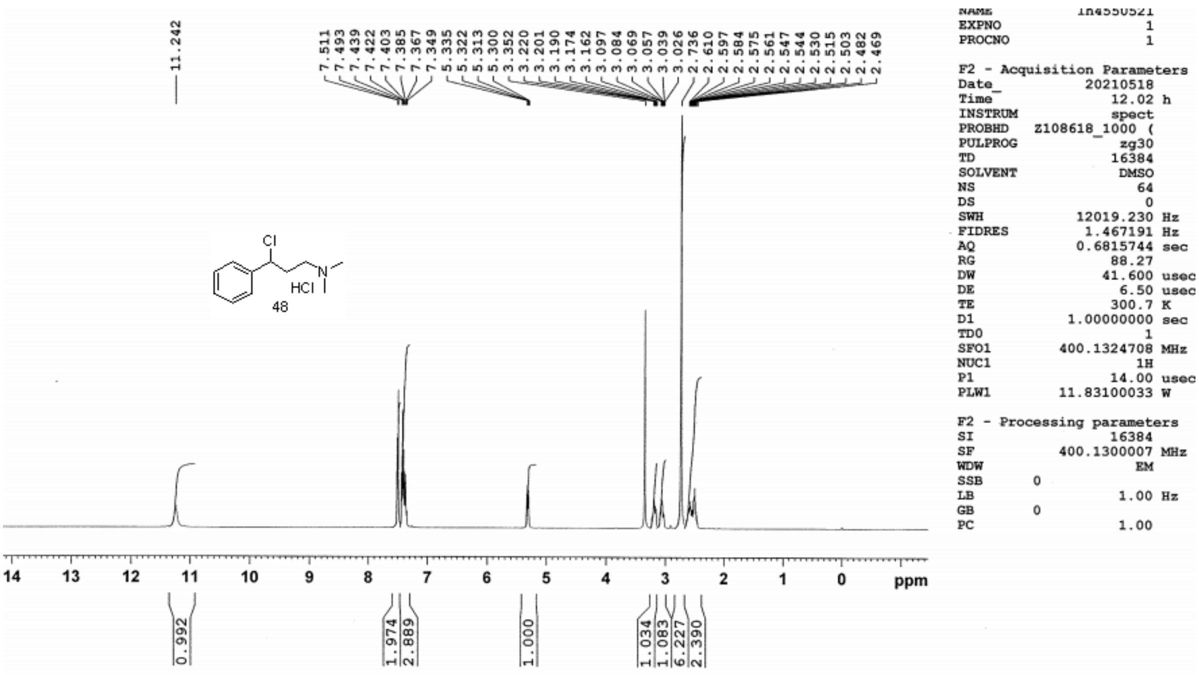

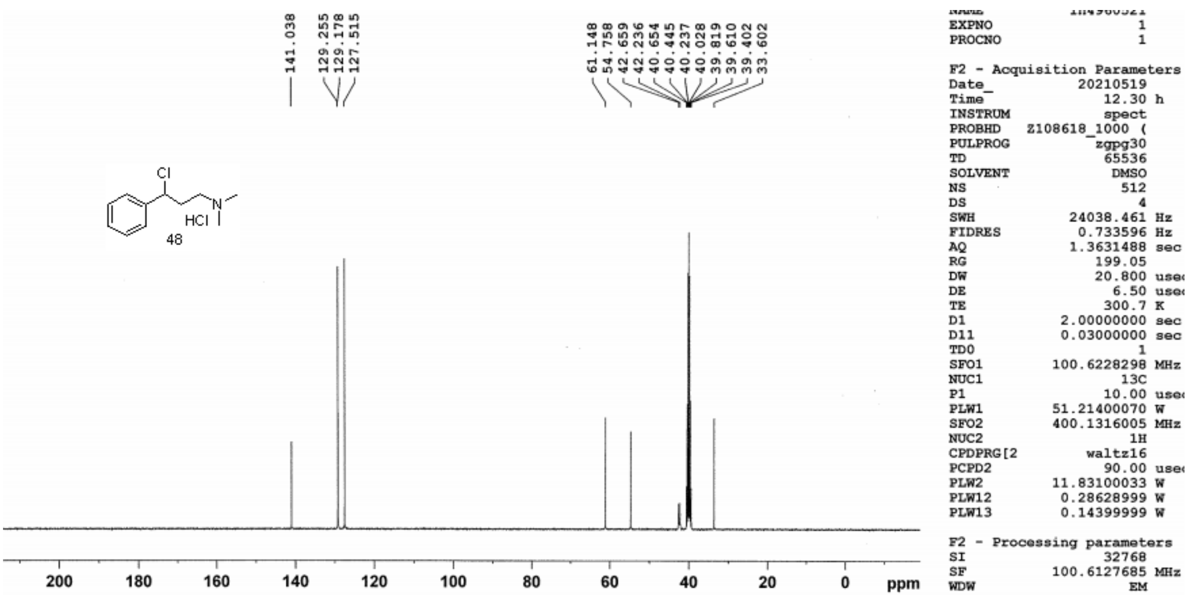

2,5-Dimethoxy-phenol **(46)**

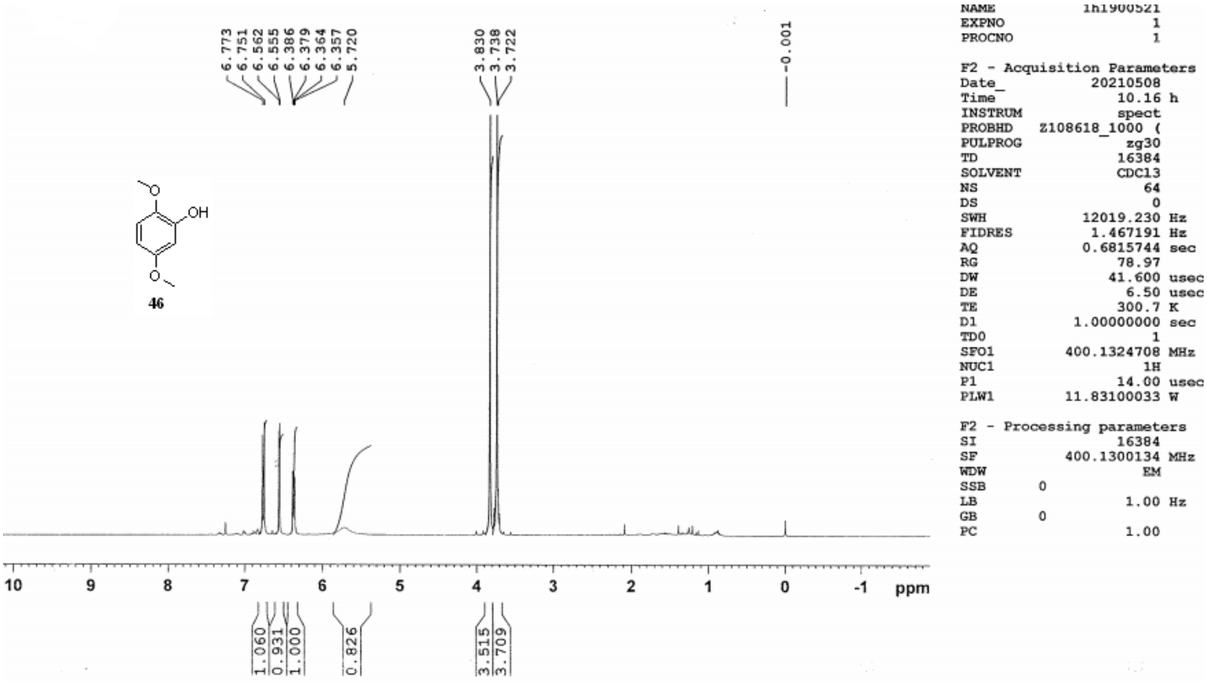

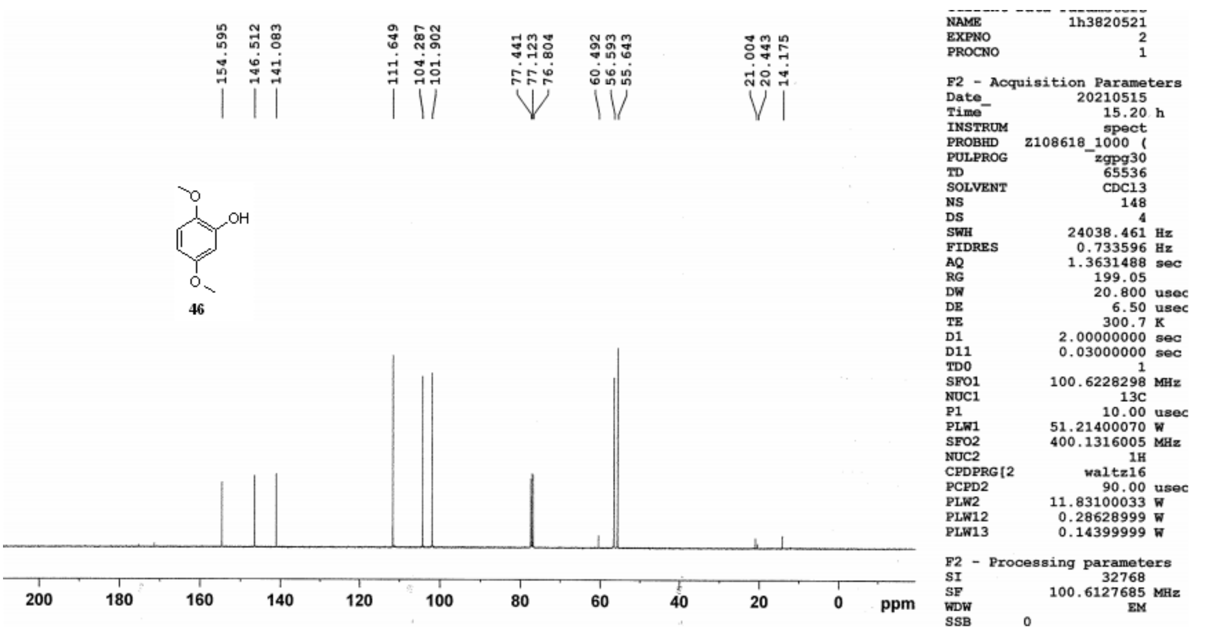

[3-(2,5-Dimethoxyphenoxy)-3-phenylpropyl]dimethylamine **(49)**

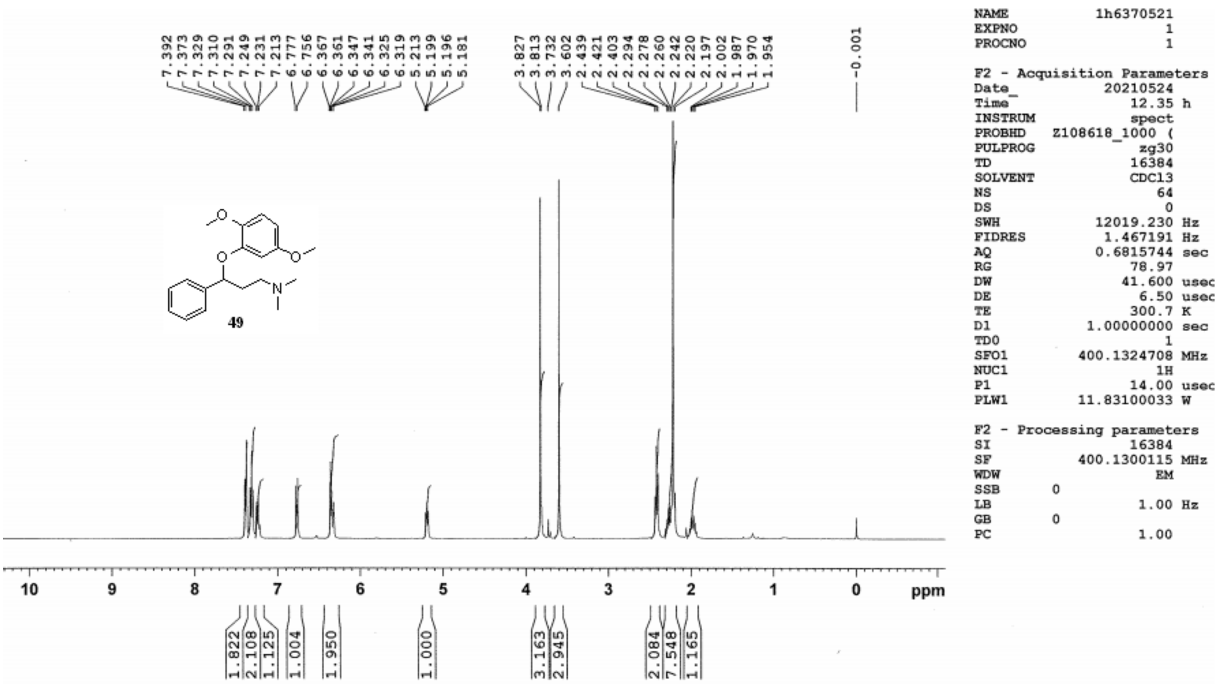

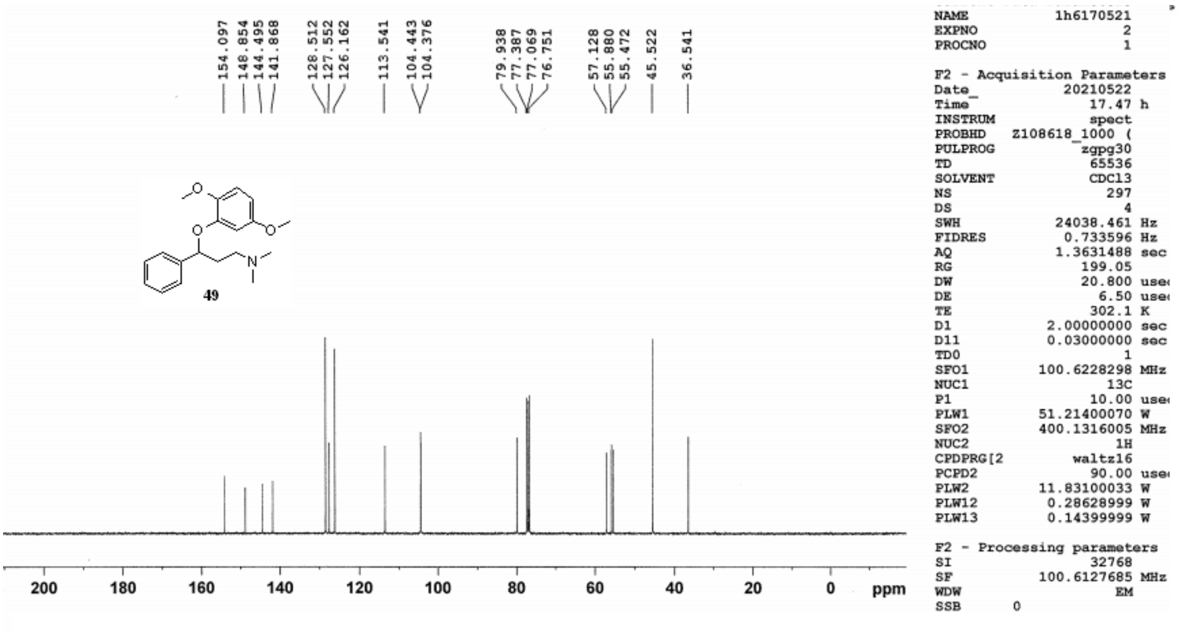

2-(3-Dimethylamino-1-phenylpropyl)-3,6-dimethoxyphenol (**50**)

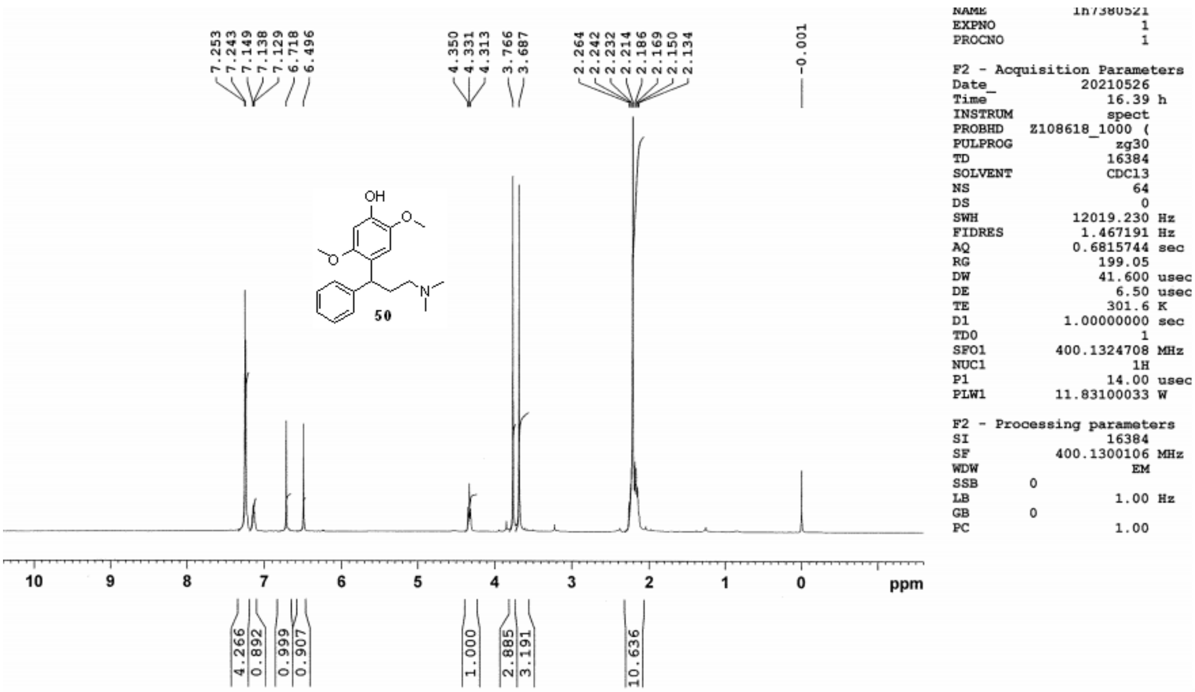

Mimosifoliol (52)

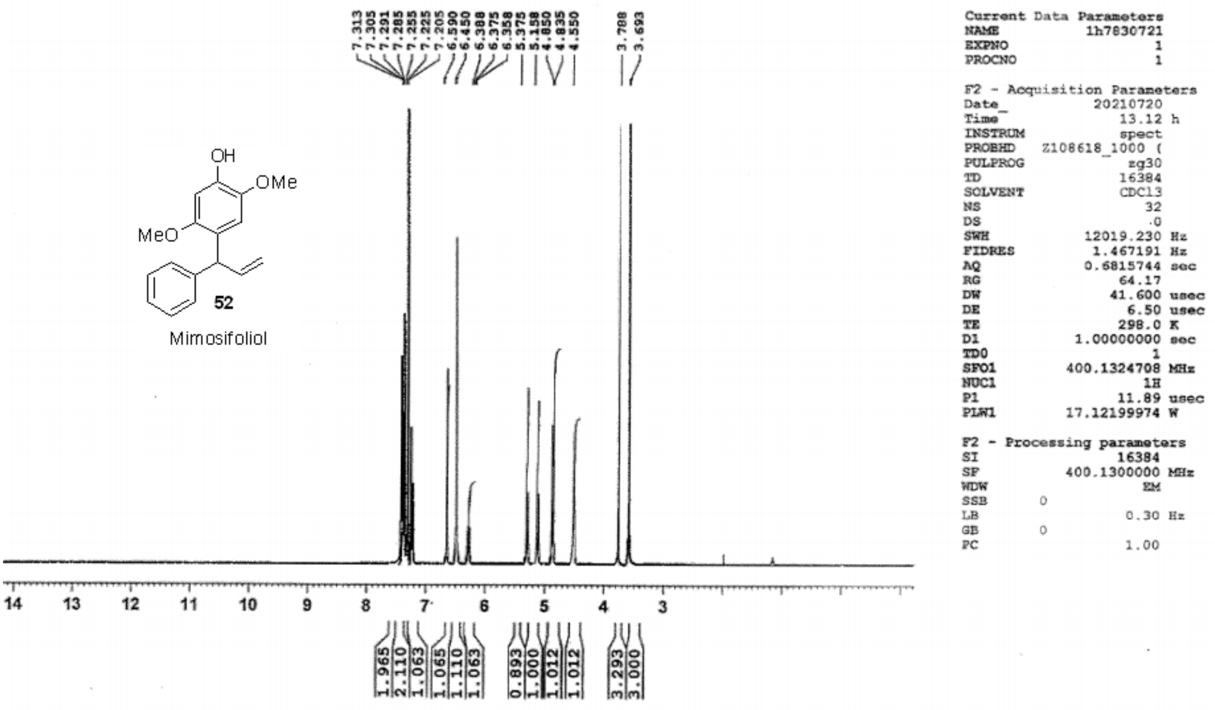

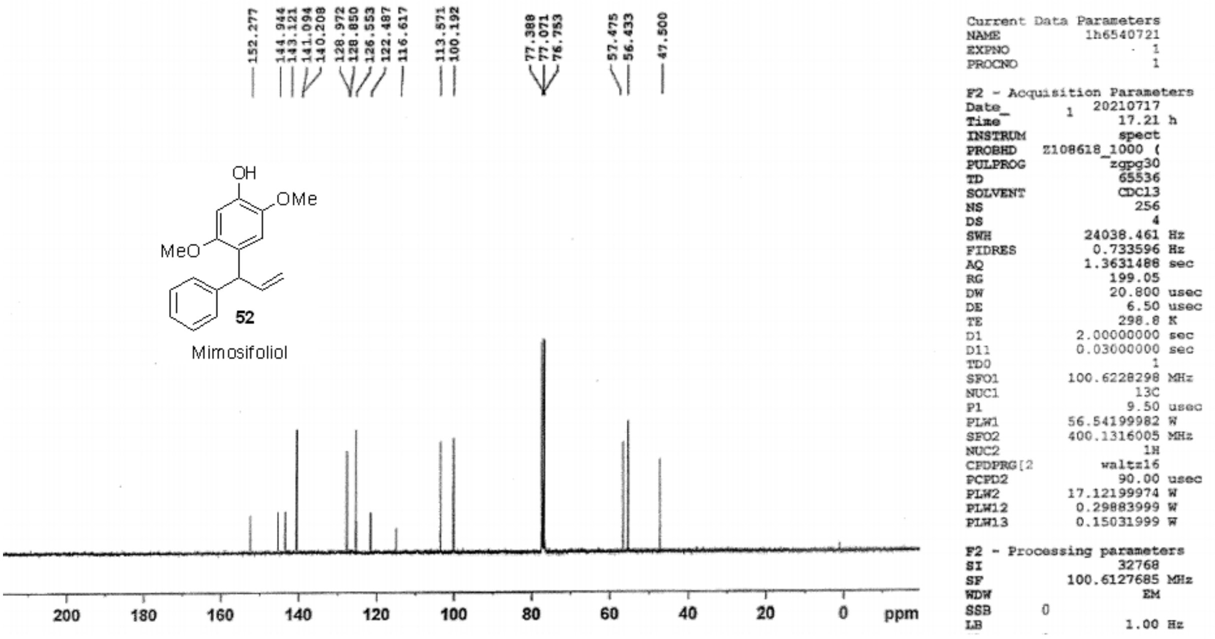

Display Report

Analysis Info

Analysis Name	D:\Data\2022\EXTERNAL\DEC\B7.d	Acquisition Date	12/3/2022 10:56:00 PM
Method	tune_low.m	Operator	UOH-Chemistry
Sample Name	B7-DCM-MEOH	Instrument	maXis 10138
Comment			

Acquisition Parameter

Source Type	ESI	Ion Polarity	Positive	Set Nebulizer	0.4 Bar
Focus	Not active	Set Capillary	3000 V	Set Dry Heater	200 °C
Scan Begin	50 m/z	Set End Plate Offset	-500 V	Set Dry Gas	6.0 l/min
Scan End	1800 m/z	Set Collision Cell RF	350.0 Vpp	Set Divert Valve	Waste

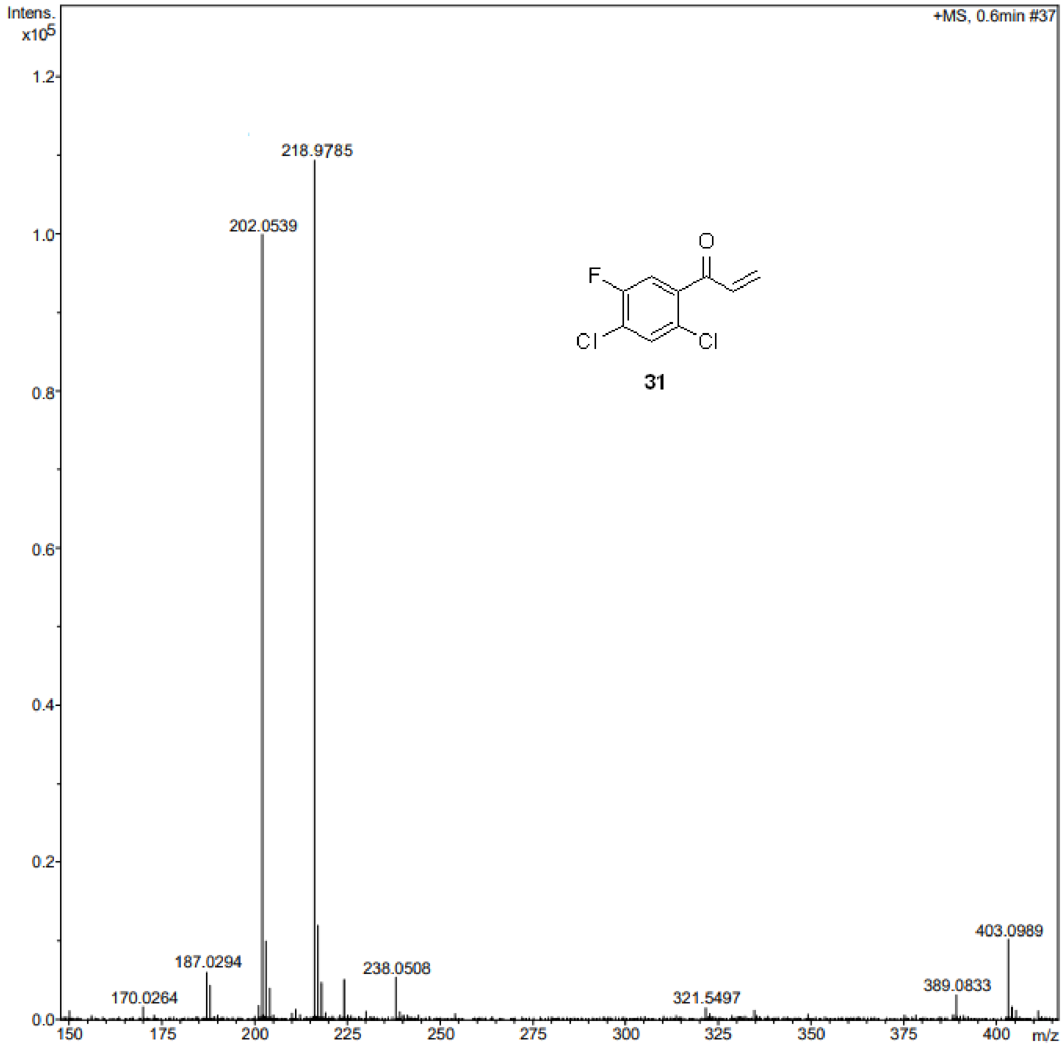

Bruker Compass DataAnalysis 4.0 printed: 12/3/2022 10:58:17 PM Page 1 of 1

Display Report

Analysis Info

Analysis Name	D:\Data\2022\EXTERNAL\DEC\B18.d	Acquisition Date	12/4/2022 11:59:46 PM
Method	tune_low.m	Operator	UOH-Chemistry
Sample Name	B18-DCM-MEOH	Instrument	maXis 10138
Comment			

Acquisition Parameter

Source Type	ESI	Ion Polarity	Positive	Set Nebulizer	0.4 Bar
Focus	Not active	Set Capillary	4200 V	Set Dry Heater	200 °C
Scan Begin	50 m/z	Set End Plate Offset	-500 V	Set Dry Gas	6.0 l/min
Scan End	1800 m/z	Set Collision Cell RF	350.0 Vpp	Set Divert Valve	Waste

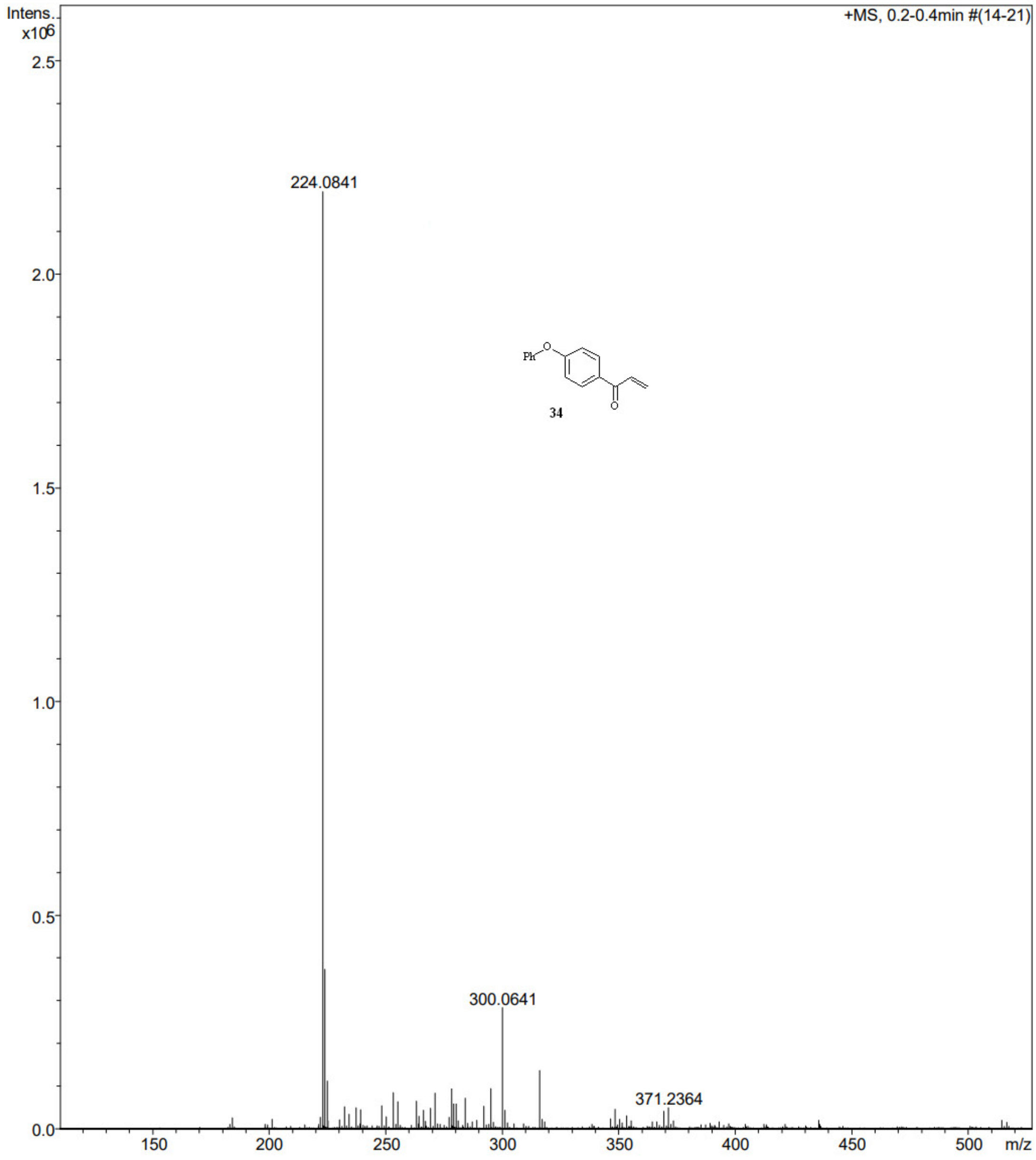

Bruker Compass DataAnalysis 4.0 printed: 12/5/2022 12:04:04 AM Page 1 of 1

Display Report

Analysis Info

Analysis Name	D:\Data\2022\EXTERNAL\DEC\B11.d	Acquisition Date	12/3/2022 11:30:45 PM
Method	tune_low_Pos.m	Operator	UOH-Chemistry
Sample Name	B11-DCM-MEOH	Instrument	maXis 10138
Comment			

Acquisition Parameter

Source Type	ESI	Ion Polarity	Positive	Set Nebulizer	0.3 Bar
Focus	Not active	Set Capillary	4200 V	Set Dry Heater	180 °C
Scan Begin	50 m/z	Set End Plate Offset	-500 V	Set Dry Gas	6.0 l/min
Scan End	1500 m/z	Set Collision Cell RF	350.0 Vpp	Set Divert Valve	Waste

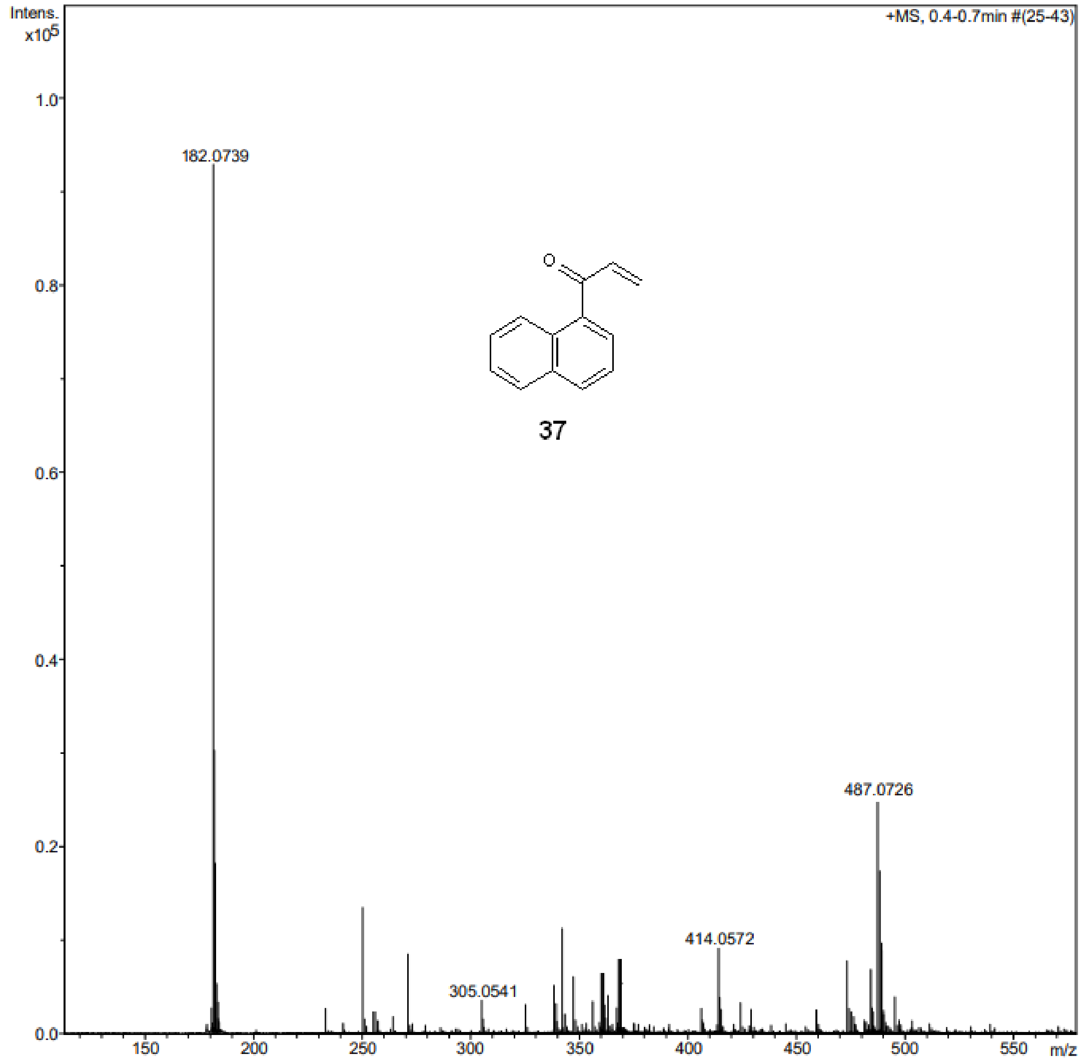

Display Report

Analysis Info

Analysis Name	D:\Data\2022\EXTERNAL\DEC\B19.d	Acquisition Date	12/5/2022 12:46:40 AM
Method	tune_low_Pos.m	Operator	UOH-Chemistry
Sample Name	B19-DCM-MEOH	Instrument	maXis 10138
Comment			

Acquisition Parameter

Source Type	ESI	Ion Polarity	Positive	Set Nebulizer	0.3 Bar
Focus	Not active	Set Capillary	4200 V	Set Dry Heater	180 °C
Scan Begin	50 m/z	Set End Plate Offset	-500 V	Set Dry Gas	6.0 l/min
Scan End	1500 m/z	Set Collision Cell RF	350.0 Vpp	Set Divert Valve	Waste

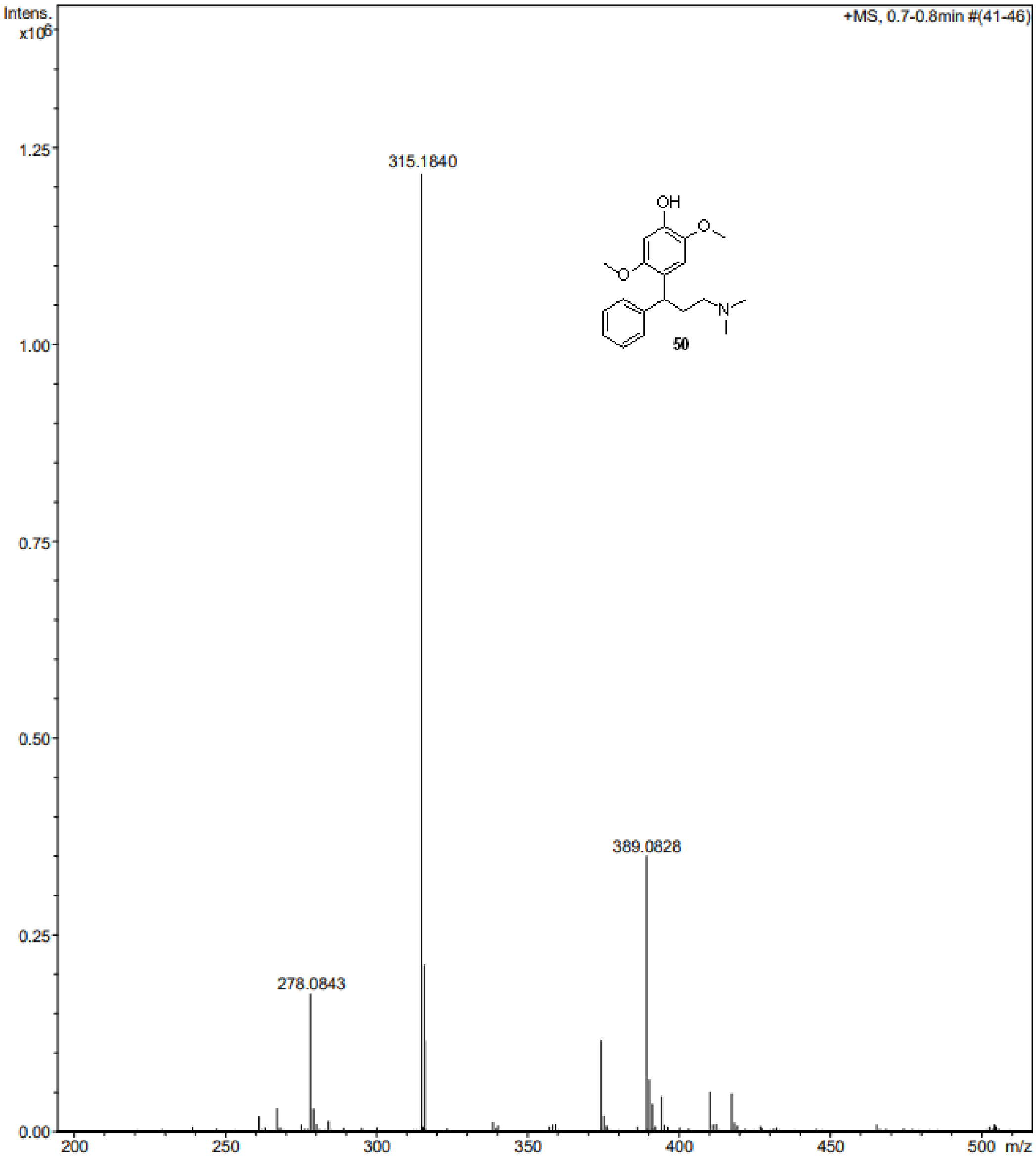

Display Report

**Analysis Info**		Acquisition Date	12/8/2021 11:59:46 PM
Analysis Name	D:\Data\2019\EXTERNAL\DEC\B18.d		
Method	tune_low.m	Operator	UOH-Chemistry
Sample Name	B18-DCM-MEOH	Instrument	maXis 10138
Comment			

Acquisition Parameter

Source Type	ESI	Ion Polarity	Positive	Set Nebulizer	0.4 Bar
Focus	Not active	Set Capillary	4200 V	Set Dry Heater	200 °C
Scan Begin	50 m/z	Set End Plate Offset	-500 V	Set Dry Gas	6.0 l/min
Scan End	1800 m/z	Set Collision Cell RF	350.0 Vpp	Set Divert Valve	Waste

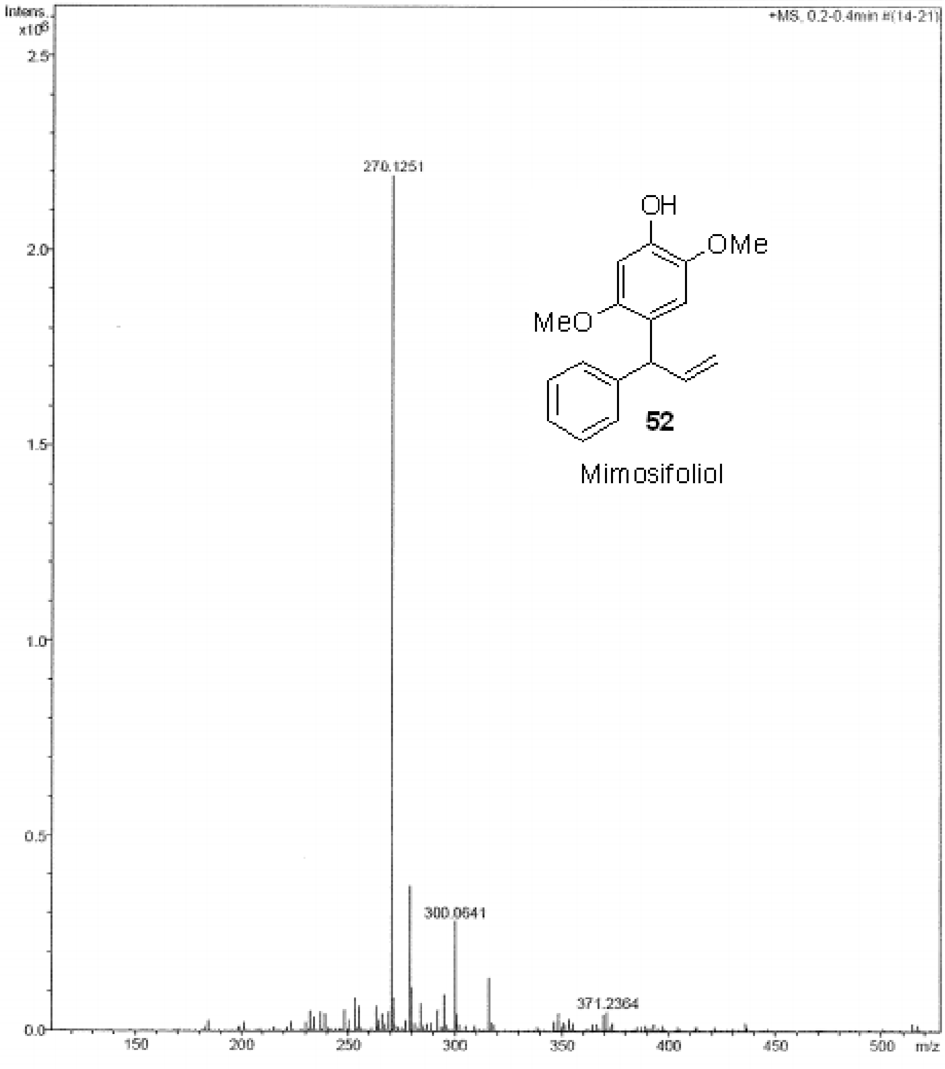

Table S1. Observed and calculated ^{13}C -NMR spectral data (δ in ppm) of compound 27-31, 35, 38 and 39

	27		28		29		30		31		35		38		39	
	Theory	Exp[a]	Theory	Exp[a]	Theory	Exp[a]	Theory	Exp[a]	Theory	Exp[a]	Theory	Exp[a]	Theory	Exp[a]	Theory	Exp[a]
12-C	193.87	190.87	199.09	194.25	194.08	189.34	197.5	192.86	196.32	191.51	196.05	194.38	192.7	190.43	192.27	189.52
1-C	142.94	137.24	147.31	138.15	133.05	126.25	145.16	135.79	146.32	155.40	163.35	163.59	140.04	134.75	141.03	139.38
6-C	134.21	-	145.83	136.16	171.67	162.44	146.47	136.47	140.57	137.79	119.95	118.57	134.39	129.32	135.14	
14-C	136.05	130.07	140.87	132.02	140.98	159.92	140.31	132.42	139.77	135.73	142	136.68	135.5	129.6	135.12	130.48
4-C	139.75	132.98	137.62	131.53	140.57	135.47	151.94	137.08	137.6	126.91	124.98	118.96	153.18	143.84	154.79	148.48
2-C	134.98	128.61	136.93	131.3	138	134.14	137.92	130.42	124.28	117.18	132.53	119.39	134.78	129.32	136.56	
16-C	138.39	132.34	136.41	130.28	137.7	130.84	137.19	130.18	138.35	132.03	134.83	130.09	137.61	132.37	139.16	135.53
5-C	132.89	128.41	134.92	129.31	120.78	116.56	134.46	127.18	136.93	124.36	139.73	130.781	133.13	128.83	133.02	128.9
3-C	133.82	-	132.06	126.73	129.45	124.42	132.29	130.18	165.05	157.91	138.14	130.6	134.39	129.32	134.28	130.05
9-C	--	--	--	--	--		--	--	--		--	--	21.84	21.63	--	--
**RMSD**	**5.61**		**6.67**		**8.46**		**8.53**		**7.7**		**6.68**		**5.31**		**4.13**	

[a]This work GIAO/B3LYP/6-311+G(2d,p) Ref to TMS

Table S2. Observed and calculated ^{13}H -NMR spectral data (δ in ppm) of compound 27-31, 35, 38 and 39

		27		28		29		30		31		35		38		39	
		Theory	Exp[a]	Theory	Exp[a]	Theory	Exp[a]	Theory	Exp[a]	Theory	Exp[a]	Theory	Exp[a]	Theory	Exp[a]	Theory	Exp[a]
7-H		8.54	7.95	7.69	7.42	8.1	7.74	7.6	7.44	7.46	7.24	8.00	12.52	8.45	7.85	8.43	7.44
11-H		8.2	--	--	--	--	--	--	--	--	--	--	--	8.1	--	8.05	--
9-H		7.83	7.59	7.66	7.42	7.78	7.51	--	--	--	---	7.6	7.49	--	--	--	--
10-H		7.69	7.49	7.56	7.42	7.33	7.03	7.49	7.38	7.67	7.51	6.81	6.92	7.47	6.43	7.54	7.14
8-H		7.75	--	7.53	7.34	7.47	7.13	7.44	7.33	--	--	7.16	7.33	7.64	7.15	7.64	--
15-H		7.71	7.19	7.27	6.79	7.51	7.22	7.22	6.78	7.23	6.79	7.77	7.79	7.66	7.26	7.57	7.44
17-H		7.07	6.46	7.01	6.15	7.06	6.38	7.03	6.17	7.07	6.21	6.94	7.00	7.02	5.88	7.09	6.43
18-H		6.22	5.94	6.11	6.03	6.16	5.9	6.14	6.07	6.2	6.11	6.02	6.57	6.18	--	6.24	5.94
19-H		--	--	--	--	--	--	--	--	--	--	4.8	5.96	2.57	2.39	--	
														2.09	--	--	
														2.6	--	--	
**RMSD**		**0.43**		**0.41**		**0.38**		**0.4**		**0.45**		**1.66**		**0.73**		**0.58**	

[a]This work GIAO/B3LYP/6-311+G(2d,p) Ref to TMS

Table S3. Observed and calculated ^{13}C -NMR spectral data (δ in ppm) of compound 32& 33

	32		33	
	Theory	Exp[a]	Theory	Exp[a]
9-C	184.29	182.31	179.22	177.43
1-C	158.54	144.53	159.89	152.61
4-C	148.7	134.42	155.59	146.95
11-C	135.62	129.32	136.52	131.17
13-C	136.66	131.8	135.75	128.86
2-C	137.05	132.55	125.4	118.25
3-C	131.64	128.35	117.02	112.29
**RMSD**	**8.43**		**6.33**	

[a]This work GIAO/B3LYP/6-311+G(2d,p) Ref to TMS

Table S4. Observed and calculated ^{1}H -NMR spectral data (δ in ppm) of compound 32& 33

	32		33	
	Theory	Exp[a]	Theory	Exp[a]
8-H	7.92	7.78	7.89	7.38
6-H	7.85	7.68	7.39	7.00
12-H	7.41	7.17	7.17	6.80
7-H	7.24	7.11	6.96	6.26
14-H	6.97	6.52	6.69	6.21
15-H	6.13	5.89	6.09	5.54
**RMSD**	**0.24**		**0.51**	

This work GIAO/B3LYP/6-311+G(2d,p) Ref to TMS

Table S5. Observed and calculated ^{13}C -NMR spectral data (δ in ppm) of compound 36& 37

	36		37	
	Theory	Exp[a]	Theory	Exp[a]
18-C/7-C	194.48	190.61	198.16	195.91
4-C	140.23	135.44	139.58	133.86
15-C	139.64	134.58	134.14	130.56
22-C	138.14	132.48	137.91	132.01
3-C	137.41	132.31	136.86	131.42
10-C	137.02	130.41	142.21	136.94
20-C	136.75	129.97	140.78	135.76
2-C	136.28	129.56	131.72	126.51
6-C	133.88	128.60	131.06	125.63
11-C	132.74	128.53	139.34	132.01
5-C	132.63	127.81	133.82	128.45
1-C	131.77	126.82	133.03	127.56
14-C	129.21	124.4	128.22	124.36
**RMSD**	**5.36**		**4.8**	

[a]This work GIAO/B3LYP/6-311+G(2d,p) Ref to TMS

Table S6. Observed and calculated ^{1}H -NMR spectral data (δ in ppm) of compound 36& 37

	36		37	
	Theory	Exp[a]	Theory	Exp[a]
7-H/18H	9.24	8.36	8.20	7.89
9-H	8.33	7.98	9.21	8.34
16-H	8.28	7.86	8.37	7.98
12-H	8.24	--	8.12	7.72
17-H	8.24	--	7.77	7.57
13-H	7.8	7.52	7.78	--
21-H	7.79	--	7.55	6.97
8-H	7.74	7.27	7.89	--
23-H	7.15	6.48	7.15	6.27
24-H	6.28	5.89	6.28	6.04
**RMSD**	**0.53**		**0.54**	

[a]This work GIAO/B3LYP/6-311+G(2d,p) Ref to TMS
